# Supplementary material for: Smooth doubly curved origami shells with reprogrammable rigidity
Source: Nat Commun. 2026 Feb 13;17:2729. doi: 10.1038/s41467-026-69562-2 (PMC13013821; doi:10.1038/s41467-026-69562-2)
Supplement: Supplementary file 1 — Supplementary Information [file 41467_2026_69562_MOESM1_ESM.pdf]

Supplementary Information for

Smooth doubly curved origami shells with reprogrammable rigidity

Morad Mirzajanzadeh[1] and Damiano Pasini[1]

[1] Department of Mechanical Engineering, McGill University; Montreal, Canada.

Corresponding author: damiano.pasini@mcgill.ca

The PDF file includes:

Supplementary Notes
Supplementary Figures 1 to 23
Supplementary References

Other Supplementary Files for this manuscript include the following:

Supplementary Movies 1 to 2
Supplementary Dataset

Supplementary Notes List

1 Curved Crease Folding **3**
1.1 Basic Definitions 3
1.2 Ruled Surfaces 3
1.3 Unfolded Pattern, Folded State and Smooth Folding 4
1.4 Smooth Developable Surfaces and Parametrizations 4

2 Construction of the '*Lens-Box*' Pattern **5**
2.1 Characterization of the '*Lens Unit*' 6
2.1.1 Smooth Folding Condition 9
2.1.2 Folded Geometry: Forward Problem 10
2.2 Characterization of the '*Waterbomb Connector*' 18
2.2.1 Flat-Foldability 19

3 Rigid-foldability **23**
3.1 Isolated Single-Vertex Fold 25
3.2 Conditions for Multi-Vertex Fold Pattern 27
3.3 Configuration Space of the Waterbomb Connector 28
3.3.1 Folding Kinematics upon Panel Contact 31
3.3.2 Kinematic Origin of Folding Difficulty 34
3.4 A Single Lens-Box Unit 35
3.5 Tessellated Lens-Box Crease Pattern 37

4 Inverse Problem **40**
4.1 Smooth Tessellation Condition 42

5 Tessellation Generation for Target Surface Mapping **44**
5.1 Generalized Cylindrical Surfaces 45
5.1.1 Constant-Curvature Tessellations 45
5.1.2 Smooth Variable-Curvature Tessellations 46
5.1.3 A Single Unit for Smooth Curvature Transition 51
5.1.4 Not- C^1 Periodic Tessellations of Smooth Unit-Cells 53
5.2 Double-Curvature Surfaces 56

6 Numerical Optimization **60**
6.1 Constraints 61
6.2 Objective Function 62
6.3 Numerical Optimization Approach 62

7 Rigidity Analysis **63**
7.1 Using Tendons to Impart Rigidity 64
7.2 Tensegrity Notions to Tune Stiffness 66

8 Experiments **66**
8.1 Fabrication and Testing Methods 66
8.2 A Gear-Based Mechanism for Adjustment of Pre-Tension in Tendons 67
8.3 Three-Point Bending Experiment 70

1 Curved Crease Folding

We formulate the folding geometry of our pattern by reviewing and expanding the collective understanding of curved-crease origami [1-5], including the analysis of the degree of curvature smoothness (e.g., C^1 and C^2) used to describe the Huffman's lens tessellation pattern [2]. We begin by defining the key terminology and notation used throughout this work, as well as outlining the underlying assumptions.

1.1 Basic Definitions

Mathematical surfaces that resemble a bent sheet of paper and can be flattened onto a plane without distortion are known as *developable surfaces*. These are smooth, tangent-continuous surfaces that belong to the class of *ruled surfaces*, and can be generated by sweeping a straight line – namely a *rule line* or *ruling* – along a space curve, the *directrix*. Since a ruled surface is curved in one direction, that of the directrix, and straight in the other, that of the ruling, they maintain a constant tangent plane along the rulings. The relative orientation between the ruling and the directrix determines the type of ruled surface, for example, a conical surface. The curved crease patterns examined in this work form ruled surfaces upon folding and are assumed to have vanishing thickness.

While developable surfaces can theoretically extend infinitely, practical applications typically involve only a finite portion. Therefore, in this work, any reference to a smooth surface should be understood as such as finite portion. The flattened version of this surface is called its *developed state*.

1.2 Ruled Surfaces

A *smooth ruled surface* is generated by a continuous one-parameter family of straight lines, known as *rulings* or *rule lines/segments*. Its discrete counterpart, referred to here as a *discrete ruled surface*, consists of a finite set of such lines. These surfaces often correspond to strips composed of triangles or planar quadrilaterals, which can similarly be unfolded without stretching or tearing. We denote their internal edges as *rulings* or *rule segments*.

We define both a *smooth* and *discrete surface* as a mapping from a subset of $\mathbb{R}^2$, the domain $T \times \mathbb{R}$, into $\mathbb{R}^3$, where T is a continuous subset of $\mathbb{R}$ for smooth surfaces, and a discrete subset of $\mathbb{R}$ for discrete ones.

1.3 Unfolded Pattern, Folded State and Smooth Folding

By drawing from the existing literature [2, 4, 6], we start to define the mathematical properties of an unfolded *piece of paper* described as an open 2-manifold embedded in $\mathbb{R}^2$. A *crease* ℓ ($\ell(s) : (0, \varphi) \to \mathbb{R}^2$ with $\varphi \in \mathbb{R}_{>0}$) is a C^2 2D curve contained in a piece of paper and not self-intersecting. A *crease point* is a point $\chi(s)$ on the relative interior of the crease that is not an endpoint; the endpoints of a crease are the *vertices*. A *crease pattern* is an embedded planar graph, where each edge forms a crease. A *face* (*facet*) is a maximal open region of the piece of paper not intersecting any creases or vertices.

3D (folded) notions. A *proper folding* of a crease pattern is a piecewise-C^2 isometric mapping of a piece of paper into a 3D form that is C^1 on every face but of course 'not-C^1' at every point and every vertex of a crease. Here, isometric means that mapping does not alter the intrinsic path lengths, and piecewise-C^2 means that the folded image can be decomposed into a finite complex of C^2 open regions joined by points and C^2 curves. We also use the terms *folded crease*, *folded vertex*, and *folded face* to denote the isometric folding mapping of creases, vertices, and faces. Thus, each folded face subdivides into a finite complex of C^2 open regions joined by points called folded semivertices and C^2 curves called folded semicreases. Each folded crease can be subdivided into a finite sequence of C^2 curves joined by C^1 points namely semikinks and by not-C^1 points, namely kinks.

Smooth folding. A *smoothly folded crease* is a folded crease that is C^1, i.e., kink-free. Demaine et al. [2] showed that a folded crease cannot have semikinks, and thus a smoothly folded crease is also C^2. A *smooth folding* of a crease pattern is a folding where every crease is smoothly folded [2].

1.4 Smooth Developable Surfaces and Parametrizations

While smooth and discrete developable surfaces can take many forms, our focus here lies on smooth surfaces and their discrete analogs, rather than on irregular shapes resembling a crumpled

paper. Smooth developable surfaces have been widely studied in classical differential geometry [7-9] and, more recently, in the context of curved-crease origami [2, 6]. According to Gauss's Theorema Egregium [10], any C^2 surface that can be flattened into a plane without distortion must have zero Gaussian curvature. At such points of vanishing Gaussian curvature, the surface is locally either flat or parabolic in nature [9]. This property allows for parametrizations using ruled surfaces with constant tangent planes along their rulings.

For the geometric construction and parametrization of our ruled surfaces, we assume that the curved crease (directrix) is everywhere properly curved with no segments of vanishing curvature. We draw inspiration from prior works – such as that of Fuchs and Tabachnikov [5], and Demaine et al. [2] – which use principles of differential geometry to derive the properties of the directrix and the adjacent developable surfaces joined along it. For a more comprehensive mathematical treatment of general curved-crease folding problems, we refer interested readers to the PhD thesis of Klara Mundilova [11].

2 Construction of the '*Lens-Box*' Pattern

As discussed in the main manuscript, our lens-box unit integrates two folding patterns: a '*lens-unit*' and a variation of the '*waterbomb*' (Fig. S1a). Its geometry consists of one lens unit and two symmetrically arranged '*waterbomb connectors'*. To examine the overall folding and kinematics, we first study them separately starting with the lens unit. To do so, it suffices to assume that during folding all free boundaries (edges) of the lens unit remain undeformed (rigid) and that the straight creases of the waterbomb connectors never bend.

In the following, we first tackle the forward problem: predict the geometry of the folded curve crease lens unit as a function of the degrees of freedom (DOFs) of its initial 2D crease pattern and design the flat-folded state of the connector unit. Later, in section S4, we solve the inverse problem, i.e., determine the geometry of the 2D patterns that can fold into predefined 3D surfaces with prescribed curvature. To address the former, we need first to characterize the folding of the lens unit and its rulings as described in the next section.

2.1 Characterization of the '*Lens Unit*'

The lens units with two curved creases in Fig. S1a and Fig. 1a are assumed to have all (straight) edge boundaries non-deformable, i.e., rigid. This idealization excludes the effect of the free-boundary panels, such as those apparent in Fig. S1b and c, which deform differently. With reference to previously established theories [2-4], the ruling segmentation and insights gained from our earlier work [1], we first prove the existence of the ruling pattern assumed for the generalized non-symmetric lens unit in Fig. 1b. Then we use differential geometry to derive the mathematical relations that can predict the spatial geometric trajectory that a crease pattern made of two initially flat C^2 arcs takes upon folding.

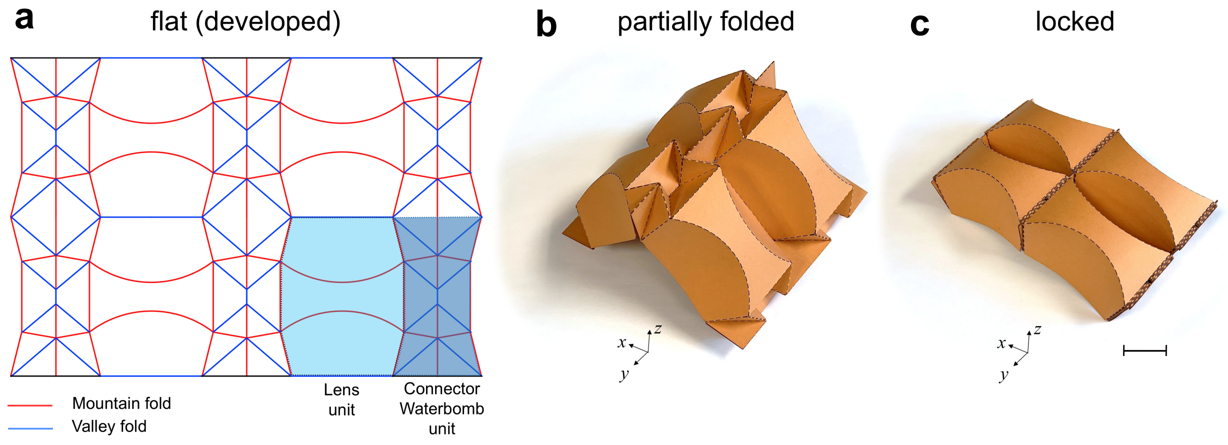

Fig. S1. Construction of a symmetric lens-box tessellation. (a) Four by four lens box tessellation pattern with identification of its mountain and valley folds and specification of the '*lens unit*' and its connector, the '*waterbomb unit*'. Paper prototype in a partially folded configuration, namely a minimum energy state at rest, where the total energy of the system, comprising the bending energy of the curved panels and the energy stored in the rotational hinges (creases), attains the minimum value (b) and upon locking (c). Panel contact of waterbomb unit impedes further folding from lock state. In (c), glue is applied to hold the lock configuration for demonstrative purposes.

Fig. S2a shows the geometry of our '*lens unit*' consisting of an upper *leg panel U*, a lower *leg panel L* and a middle *lens panel M*. The *lens unit* is assumed to fold symmetrically with respect to the symmetry plane of the curved *M* face that passes through the *x*-axis, making *U* a reflection of *L*. With this assumption, the folding of our lens-unit can be inferred by the change in distance from the straight edges of *U* to *L* occurring between two given states, e.g., transition and initial.

To construct the folded geometry and analyze the folding kinematics, we adopt simplifying assumptions to enable a tractable mathematical formulation. Specifically, we assume that the

ruling pattern remains invariant throughout the folding process – a condition referred to as *rigid-ruling foldability* [2] – and that the folding is symmetric. We also assume that the curved creases fold smoothly, which is the energetically preferred mode for developable surface, as explained in the main manuscript. Relaxing these assumptions would substantially increase the complexity of both the folded geometry and the associated folding kinematics, as the ruling pattern may otherwise evolve during folding to maintain developability. Attempting to tackle such complexity is unnecessary for the purpose of this work.

The validity of the assumption of *rigid-ruling foldability* is supported by the uniqueness of the locked configuration. While multiple ruling patterns may exist during folding, in the locked state, only a unique ruling pattern – and thus a single folded configuration – is possible. This configuration corresponds to the locked geometry and ruling pattern identified in Fig. 1b. If we assume that the connector units consist of rigid panels and the lens unit is discretized by rigid ruled segments, we can demonstrate through a structural rigidity analysis (see section S7) that the locked origami has zero mobility (DOFs). Any alternative ruling pattern would yield a dissimilar locked geometry, which is an unfeasible outcome given the system's zero DOFs. As a result, our assumptions and ruling pattern naturally emerge as the only valid constraints of the locked configuration.

Nevertheless, the existence of a unique locked state alone does not guarantee the rigid-foldability of the origami pattern. We must also ensure that all intermediate folding states exist continuously from the unfolded, flat state to the locked state. This, in turn, requires demonstrating that the pattern can fold continuously without altering the ruling pattern, a property guaranteed by rigid-ruling foldability.

We now use the qualitative properties of the rulings obtained for the generalized version of Huffman's *lens* motif by Demaine et al. [2] to reconstruct the rulings of our lens unit crease, which is shown in Fig. S2a and Fig. 1b. Through Corollary 26[1] of Theorem 25 [2], we realize that both curved creases must follow the same folding direction.

[1] "Consider two smoothly folded creases connected by a rule segment. If the rule segment is on the concave sides of both creases, or on the convex sides of both creases, then the creases must have the same direction (mountain or valley). If a rule segment is on the convex side of one crease and the concave side of the other crease, then the creases must have the opposite direction (one mountain and one valley)" [2]

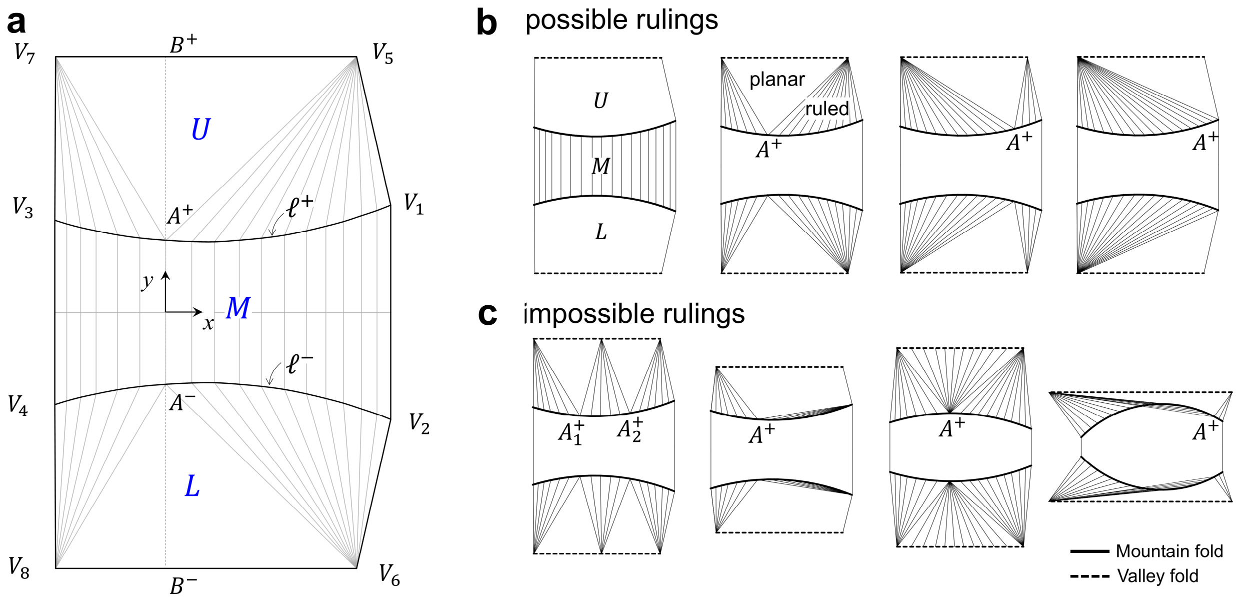

Fig. S2. Ruling conditions for the lens unit. (a) A valid ruling layout and the corresponding notation for the vertices. (b) From left to right: schematic of the symmetric lens unit comprising two leg panels and a lens panel with rulings, and other three feasible ruling patterns of the leg panels. (c) From left to right, four unfeasible cases of cone ruling: A cone apex located on one straight crease generating a kink on the rigid edge; A cone apex located at one vertex, i.e., an end, of the curve crease creating either a zero-length rule segment or a ruling tangent to the curve at the vertex; A cone apex located on the curve crease, causing bending on the assumed rigid edge of the leg panel; Intersecting rule segments crossing the curve crease.

To characterize the rulings in our lens unit, we first specify the notation. We define the vertices of the lens unit illustrated in Fig. S2a as the points of the form V_i with $i \in \mathbb{Z}$, and use capital *italic* letters with superscript $^+$ and $^-$ to refer to any crease points which are not a vertex on the upper half or lower half of the pattern, respectively, where two halves are identified by the symmetry plane of the general pattern. For example, A^+ refers to a crease point where two rule segments emanate from the upper curve crease (see Fig. S2). We also adopt a similar superscript $^+$ and $^-$ to refer to our two curve creases $\ell^- \equiv -\ell$ and $\ell^+ \equiv \ell$.

The lens panel M is bounded between two straight creases $\overline{V_1V_2}$ and $\overline{V_3V_4}$ and two curve creases ℓ^- and ℓ^+ through which the L and U leg panels join, as shown in Fig. S2a. The leg panels L and U are confined from other edges by three straight creases to avoid bending at those edges. This implies the existence of a planar region and cone rulings that impede the bending of the straight creases. As per Theorem 15 [2] in a smoothly folded crease that is C^1, i.e., kink-free, a cone ruling with an apex is not admissible on a curved crease since such a point is a kink. Similarly, the cone ruling apex on the straight creases can be proved to also yield a kink. As a result, in a

smoothly folded lens unit, the cone ruling cannot emanate from any point on the curve or on the straight creases (see Fig. 2c), and the cone ruling apices must lie at either one or more vertices. For example, a cone ruling apex in the upper leg panel U (Fig. 2a) can lie on either V_1, V_3, V_5, or V_7 or on more than one of these vertices concurrently.

To rule out the adoption of unfeasible ruling patterns, we now verify the existence of two properties that define the folding of a curve crease. The first is the bisection property of a curved crease (see [2] for proof). This property prevents from the emergence of irregular cases where a cone ruling apex locates at the start and end of a curved crease vertices, i.e., V_1 and V_3, as shown in Fig. S2c. This leaves only one possible ruling choice comprising two unique cone ruled surfaces with their cone apices located on the two other vertices, i.e., V_5 and V_7, separating a triangular planar region from the curve crease (Fig. S2a).

A second condition to ensure proper ruling is to guarantee the visibility (as defined below) of the vertices connected to the curved crease through rule segments [2]. A vertex on an unfolded curved crease is visible if the rule segment does not intersect the curved crease as illustrated with a counter example in Fig. S2c (right).

2.1.1 Smooth Folding Condition

To define the condition that guarantees smooth folding, we examine a 90-degree rotated version of the lower half of our lens unit with its curved crease (red) and ruling pattern in the unfolded configuration (left) and in a folded 3D state (right) (Fig. S3). $\mathbf{t}$ and $\mathbf{T}$ denote the tangent vectors at a crease point A^- in the unfolded and folded state, respectively. For the former, the following angle relation holds $\angle(-\mathbf{t}, \mathbf{r}_1) + \angle(\mathbf{r}_1, \mathbf{r}_2) + \angle(\mathbf{r}_2, \mathbf{t}) = \angle(-\mathbf{t}, \mathbf{t}) = 180°$. In the folded configuration, the leg surface on the left side of the curved crease is C^2 and consists of two ruled cones and one triangular planar region, which must meet smoothly at the crease point A^- with a common surface normal $\mathbf{N}_P$. As a result, the tangent to the curve crease at A^- must lie on the planar region, implying $\mathbf{N}_P.\mathbf{T} = \mathbf{0}$, and the total angle between the backward and forward tangents of the folded curved crease, $\angle(-\mathbf{T}, \mathbf{T}) = 180°$, must equal the sum of the three angles of the surfaces at A^-, i.e., $\angle(-\mathbf{T}, \mathbf{R}_1) + \angle(\mathbf{R}_1, \mathbf{R}_2) + \angle(\mathbf{R}_2, \mathbf{T}) = \angle(-\mathbf{T}, \mathbf{T}) = 180°$ (inset in Fig. S3)[2]. This shows that the $180°$ angle between the backward and forward tangents is preserved during folding.

[2] In a C^1 surface, geodesic (2D) angles equal Euclidean (3D) angles [2].

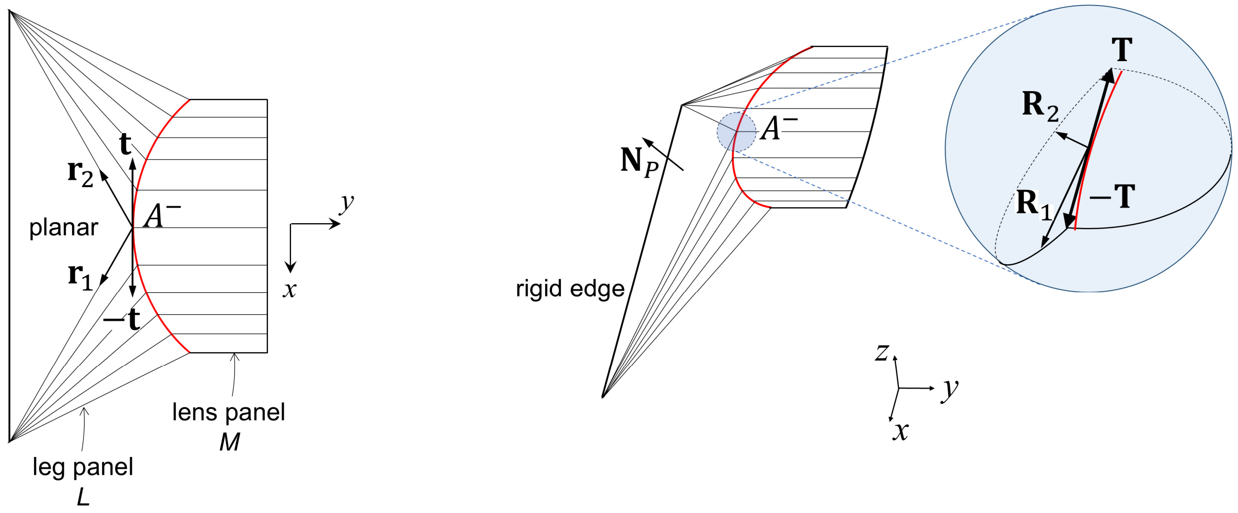

Fig. S3. Lower half of the lens-unit with its ruling pattern. Left: unfolded lens unit with leg panel L described by two ruled surfaces (cone rulings) bounding a planar triangular region; Right: smoothly folded lens unit with zoom inset magnifying the condition at the crease point A^- shared by the three surfaces.

The above has focused on the ruling pattern of the unfolded lens unit without specifying the location of the apex A^- or A^+, a crease point demarcating the connection of the two cone ruled surfaces on the curved crease. In the next section, we examine a curved crease lens unit that is smoothly folded and demonstrate that the location of A^- or A^+ is governed by the geometry of the folded lens unit.

2.1.2 Folded Geometry: Forward Problem

Let us first define the curve crease using a general non-intersecting concave or convex 2D C^2 function (Fig. S4a), which we subdivide through the y-axis passing through the apices A^+ and A^- of the triangular planar regions into a *right* and a *left* portion, each denoted with the superscript ^{L} and ^{R} respectively. We also mirror the left-curve with respect to the y-axis, and denote the curve crease with a parametrized function $\ell(s)\colon [0, u_c] \to [0, \infty)$, with $\ell(0) = \ell_o$ and $\ell(u_c) = \ell_c$. The upper crease is described by $\ell^+ = \ell(s)$, and the lower crease by $\ell^- = -\ell(s)$.

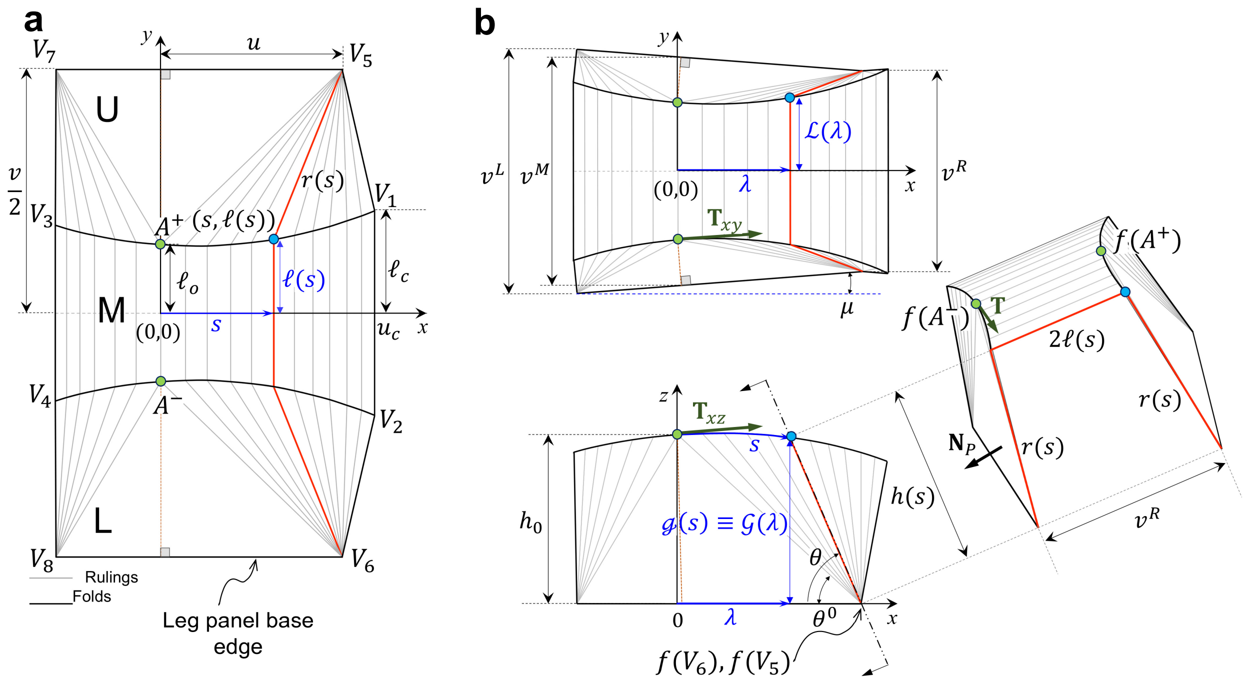

Fig. S4. Geometric construction of the lens unit. (a) Unfolded pattern construction illustrating the mountain and valley fold lines, and the rulings for the curved crease lens unit. (b) Folded pattern illustrating the projection of the folded unit on *xy*-plane (top), *xz*-plane (bottom) and a plane sliced from the highlighted red rule segments (right) denoting the variables we use in the formulation.

The continuity and smoothness of the general curved crease comprising a right-curve and a left-curve, yields $\ell^L(0) = \ell^R(0) = \ell_o$ and $\left.\frac{d\ell^L}{ds}\right|_{s=0} = -\left.\frac{d\ell^R}{ds}\right|_{s=0}$. We assume that our folding mapping, f, is isometric, i.e., length preserving, and thus, the folding $f(M)$ is a cylindrical surface with parallel rulings. We introduce the initial unfolded geometrical parameters $v = \|B^+B^-\| = \|V_5V_6\| = \|V_7V_8\|$, $u^R = \|B^+V_5\| = \|B^-V_6\|$, $u^L = \|f(B^+)f(V_7)\| = \|f(B^-)f(V_8)\|$, $v^R = \|f(V_5)f(V_6)\|$, $v^L = \|f(V_7)f(V_8)\|$ and $v^M = \|f(B^+)f(B^-)\|$ with $0 < v^R < v$ and $0 < v^M < v$. We also define the *front side* of a paper as the side where the curve creases are mountain folds. With this notation, our lens-box is convex if the front side has positive curvature, and concave if it has negative curvature, e.g., the folded lens-box unit in Fig. S3 is concave as opposed to the pattern in Fig. S1a and Fig. S4. This is equivalent to state that our lens-box is convex whenever we have concave curves in 2D, and concave when the 2D curves are convex. The relations we present hereafter are general and cover both concave and convex lens-boxes. In our analysis, when we use plus-minus sign (i.e., $\pm$ or $\mp$), the upper sign choice refers to the convex lens-box, e.g., see Fig. S4, and the lower sign choice to the concave case.

We first state the main assumptions of our analysis. First, M is ruled with segments parallel to the y axis and parametrized by s in the form of $(s, \ell(s))$. Second, U has one cone rulings between V_5 and $(s, \ell^R(s))$ and another one between V_7 and $(s, \ell^L(s))$, separated by a triangular planer region $A^+V_5V_7$. Similarly, L has one cone rulings between V_6 and $(s, -\ell^R(s))$ and another one between V_8 and $(s, -\ell^L(s))$, separated by a planer triangular region $A^-V_6V_8$. We orient our pattern such that its ruling directions in M are parallel to the y-axis.

We also assume symmetry of the folded pattern with respect to the xz-plane and orient it such that $\overline{V_5V_7}$ and $\overline{V_6V_8}$ edges (later we show they are creases) stay in the xy-plane during the entire folding. As a result, the orthogonal projection of $f(M)$ to the xz-plane is a curve $\mathcal{g}$, and a ruling at s on M corresponds to a point on $\mathcal{g}(s)$ with s being the arc-length parameter. We also depict in Fig. S4 the orthogonal projection of $f(M)$ to the xz-plane using the parametric curve $\mathcal{G}(\lambda)$ and to the xy-plane using the parametric curve $\mathcal{L}(\lambda)$ with λ being the projection of the curve $\mathcal{g}(s)$ on the x-axis. Since the arc-length s is a function of λ, i.e., $s = s(\lambda)$, we can write $\mathcal{G}(\lambda) = \mathcal{g}(s(\lambda)) = g \circ \lambda$ and $\mathcal{L}(\lambda) = \ell(s(\lambda)) = \ell \circ \lambda$.

With the assumptions above, we can now assess the necessary condition ($\mathbf{N}_P . \mathbf{T} = \mathbf{0}$) that satisfies the existence of a smoothly folded lens unit. To obtain the normal vector $\mathbf{N}_P$, we refer to Fig. S5 which shows the folded geometry of the lens unit in the Cartesian coordinate system defined by the unit vectors $(\mathbf{e}_1, \mathbf{e}_2, \mathbf{e}_3)$. Let us also denote the angle between the positive direction of the y-axis and $\overrightarrow{f(B^-)f(A^-)}$ with ς and the angle between the positive direction of the x-axis and $\overrightarrow{f(V_8)f(V_6)}$ with μ (measured as positive in the counterclockwise direction, hence $\mu > 0$ if $v^M > v^R$, and $\mu < 0$ if $v^M < v^R$). If the normal unit vector of the triangular planar region $f(V_8)f(A^-)f(V_6)$ is denoted as $\mathbf{N}_P = \hat{m} \times \hat{n}$, where $\hat{m} = \overrightarrow{f(V_8)f(V_6)} / \left\| \overrightarrow{f(V_8)f(V_6)} \right\|$ and $\hat{n} = \overrightarrow{f(B^-)f(A^-)} / \left\| \overrightarrow{f(B^-)f(A^-)} \right\|$, then we can write $\hat{m}.\mathbf{e}_1 = \cos\mu$ and $\hat{n}.\mathbf{e}_2 = \cos\varsigma$ which directly gives $\hat{m} = (\cos\mu, \sin\mu, 0)$. Upon defining the vector $\overrightarrow{f(B^-)f(A^-)}$ components as $\overrightarrow{f(B^-)f(A^-)} = (b_1, b_2, b_3)$ we can rewrite $\hat{n} = \frac{1}{\left\| \overrightarrow{f(B^-)f(A^-)} \right\|}(b_1, b_2, b_3)$. Applying the relations $\hat{m}.\hat{n} = 0$ (since $\hat{m} \perp \hat{n}$) and $\cos\varsigma = \frac{(\frac{v^M}{2} - \ell_o)}{(v/2 - \ell_o)}$ with $\left\| \overrightarrow{f(B^-)f(A^-)} \right\| = \left(\frac{v}{2} - \ell_o\right)$, gives vector $\hat{n}$ as $\hat{n} = \frac{1}{(v/2-\ell_o)}\left(-(\frac{v^M}{2} - \ell_o)\tan\mu, \frac{v^M}{2} - \ell_o, \sqrt{\left(\frac{v}{2} - \ell_o\right)^2 - \left(\frac{v^M}{2} - \ell_o\right)^2 / \cos^2\mu}\right)$.

To obtain the tangent vector $\mathbf{T}$, we recall the isometry of our folding mapping, which entails a linear transformation that is length preserving. This makes the projection of the tangent vector to the folded 3D curved crease to remain always tangent to its projected 2D curve. We write the components of the tangent vectors to the folded (lower) curved crease as $\mathbf{T} = (t_1, t_2, t_3)$, and use the geometrical parameters in Fig. S5b to obtain its *xy*-plane projection as $\mathbf{T}_{xy} = \|\mathbf{T}_{xy}\|(\cos\gamma^*, \sin\gamma^*, 0)$ and *xz*-plane projection as $\mathbf{T}_{xz} = \|\mathbf{T}_{xz}\|(\cos\gamma^{**}, 0, \sin\gamma^{**})$, where $\gamma^* = \tan^{-1}(-\mathcal{L}'(0))$ and $\gamma^{**} = \tan^{-1}(\mathcal{G}'(0))$ with $\mathcal{L}'(0) \equiv \frac{d\mathcal{L}(\lambda)}{d\lambda}\Big|_{\lambda=0}$ and $\mathcal{G}'(0) \equiv \frac{d\mathcal{G}(\lambda)}{d\lambda}\Big|_{\lambda=0}$. Upon projecting the tangent vector $\mathbf{T}$ onto *xy*-plane and *xz*-plane, we obtain the component of $\mathbf{T}$ as $t_1 = \|\mathbf{T}_{xy}\|\cos\gamma^*$, $t_2 = \|\mathbf{T}_{xy}\|\sin\gamma^*$, and $t_3 = \|\mathbf{T}_{xz}\|\sin\gamma^{**}$, where $\|\mathbf{T}_{xz}\| = \|\mathbf{T}_{xy}\|\frac{\cos\gamma^*}{\cos\gamma^{**}}$. This allows to write the tangent vector $\mathbf{T} = \|\mathbf{T}_{xy}\|(\cos\gamma^*, \sin\gamma^*, \cos\gamma^*\tan\gamma^{**})$ in terms of $\mathcal{L}$ and $\mathcal{G}$ as

$$\mathbf{T} = \frac{\|\mathbf{T}_{xy}\|}{\sqrt{1+\mathcal{L}'(0)^2}}(1, -\mathcal{L}'(0), \mathcal{G}'(0)). \tag{1}$$

The relation $\mathbf{N}_P.\mathbf{T} = 0$ can be also rewritten as $\mathbf{N}_P.\hat{n} = \mathbf{0}$, and upon replacing $\mathbf{N}_P = \hat{m} \times \mathbf{T}$, the relation becomes $(\hat{m} \times \mathbf{T}).\hat{n} = 0$. Substituting $\hat{m}$, $\hat{n}$ and $\mathbf{T}$ in the previous equation gives the smooth folding condition for our lens unit in terms of $\mathcal{L}'(0)$ and $\mathcal{G}'(0)$ as

$$\mathcal{L}'(0) + \frac{\frac{v^M}{2} - \ell_o}{\cos\mu\sqrt{\cos^2\mu\left(\frac{v}{2} - \ell_o\right)^2 - \left(\frac{v^M}{2} - \ell_o\right)^2}}\mathcal{G}'(0) + \tan\mu = 0. \tag{2}$$

From relation (2), we now derive the function $\mathcal{L}$ with respect to the unfolded curve ℓ by leveraging the isometric property of the folding mapping (Fig. S4). Since the lengths are preserved during folding, we can write $\mathcal{L}(\lambda) = \ell(s)$ where s is a function of λ, $s(\lambda)$, and can write $\mathcal{L}(\lambda)$ as a composite function $\mathcal{L}(\lambda) = \ell(s(\lambda)) = \ell \circ s$. As a result, the first derivative of $\mathcal{L}(\lambda)$ with respect to λ can be calculated through the chain rule as $\frac{d\mathcal{L}(\lambda)}{d\lambda} = \frac{d\ell(s(\lambda))}{d\lambda} = \frac{d\ell(s)}{ds}.\frac{ds(\lambda)}{d\lambda}$. Similarly, we can write $\mathcal{G}(\lambda) = g(s(\lambda)) = g \circ s$ and obtain the relation $\frac{d\mathcal{G}(\lambda)}{d\lambda} = \frac{dg(s(\lambda))}{d\lambda} = \frac{dg(s)}{ds}.\frac{ds(\lambda)}{d\lambda}$. Given $s(\lambda)$

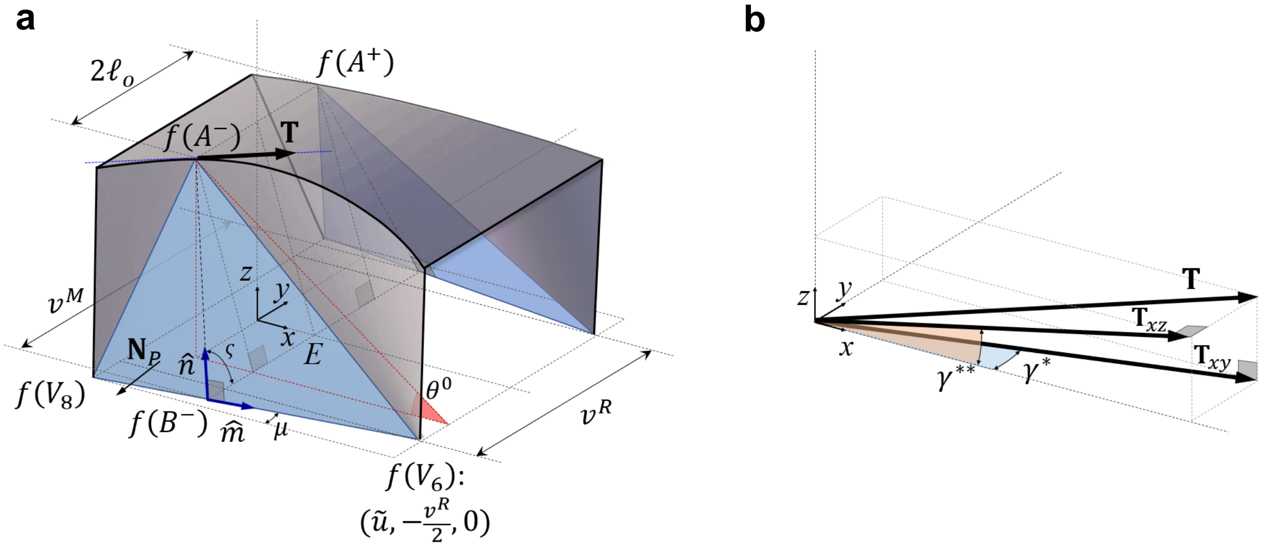

Fig. S5. Geometric construction of folded lens unit. (a) 3D folded lens unit illustrating the geometrical parameters below the curved crease in the planar region upon folding. (b) Representation of the tangent vector **T** and its projection onto the *xy*-plane and *xz*-plane which are shown here respectively with $\mathbf{T}_{xy}$ and $\mathbf{T}_{xz}$.

is the arc-length of the function $\mathcal{G}(\lambda)$, we obtain $\frac{ds(\lambda)}{d\lambda}=\sqrt{1+\left(\frac{d\mathcal{G}(\lambda)}{d\lambda}\right)^2}=\sqrt{\frac{1}{1-\left(\frac{d\mathcal{g}(s)}{ds}\right)^2}}$, which substituted in Eq. (2) yields the following constraint equation for smooth folding:

$$\frac{1}{\sqrt{1-\left(\frac{d\mathcal{g}(s)}{ds}\right)^2}}\left(\frac{d\ell(s)}{ds}+\frac{\frac{v^M}{2}-\ell_o}{\cos^2\mu\sqrt{\left(\frac{v}{2}-\ell_o\right)^2-\frac{\left(\frac{v^M}{2}-\ell_o\right)^2}{\cos^2\mu}}}\frac{d\mathcal{g}(s)}{ds}\right)+\tan\mu=0, \qquad (3)$$

which must be satisfied at $s=0$ with $v^M=v^R-2u\sin\mu$. Relations (3) and (2) are valid only for folded configurations where $v^M\neq v$. Relation (3) plays a key role for determining the mobility (DOF) of the equivalent rigid-ruling foldable system of our curved-crease lens unit.

In the following, we derive the closed form relations that enable to express $\mathcal{g}(s)$ in terms of the unfolded curved crease $\ell(s)$ and other geometrical parameters. We consider the set of rule segments of *U*, *M* and *L* for the curve parameter *s* and their folded states illustrated with the thick

red line in Fig. S4b. Due to symmetry, the rule segments form a planar polyline which together with segment $\overline{f(V_5)f(V_6)}$ form an isosceles trapezoid with the base length v^R and the top length $2\ell(s)$. The trapezoid legs are the length of the rule segments, which can be calculated for the right-curve as

$$r(s) = \sqrt{(u - s)^2 + (\frac{v}{2} - \ell(s))^2}. \tag{4}$$

The height of the trapezoid $h(s)$ can be calculated as

$$h(s) = \sqrt{(u - s)^2 + (v - v^R)(\frac{v + v^R}{4} - \ell(s))}. \tag{5}$$

Since the rulings in Fig. S4a are isometric during folding, and the pattern is symmetric relative to the *zx*-plane, we can assess the existence of the folded configuration in the constructed folded crease $f(\ell(s))$ (Lemmas 29 and 30 of [2]). This requires the distance between $f(\ell^{R+}(s))$ and vertex V_5, and also $f(\ell^{R-}(s))$ and vertex V_6 to be always $r(s)$, and the distance between $f(\ell(s))$ and *zx*-plane to be always $\ell(s)$.

The projection of the folded curve crease on the *xz*-plane can be represented in polar coordinates $(\theta(s), h(s))$ with $(\theta \in \mathbb{R})$, so as to satisfy two conditions: (*i*) the curve has arclength *s*, and (*ii*) $\theta(s)$ is a monotonic function (i.e., $\frac{d\theta(s)}{ds} > 0$) avoiding self-intersection. Using condition (*i*) we can write the following differential equation

$$(ds)^2 = (dh(s))^2 + (h(s)d\theta(s))^2, \tag{6}$$

which reduces to

$$1 = \left(\frac{dh(s)}{ds}\right)^2 + h^2\left(\frac{d\theta(s)}{ds}\right)^2. \tag{7}$$

Using condition (*ii*) and $h(s) > 0$, the above equation reduces to

$$\frac{d\theta(s)}{ds} = \frac{1}{h(s)}\sqrt{1 - \left(\frac{dh(s)}{ds}\right)^2}, \tag{8}$$

with solution

$$\theta(s) = \theta^0 + \int_0^s \frac{1}{h(s)}\sqrt{1 - \left(\frac{dh(s)}{ds}\right)^2}\, ds, \tag{9}$$

if and only if $\left(\frac{dh(s)}{ds}\right)^2 \leq 1$ for $s \in (0, u_c)$. This condition, combined with condition (*ii*), leads to $\left(\frac{dh(s)}{ds}\right)^2 < 1$, which can be expanded as

$$\left(\frac{dh(s)}{ds}\right)^2 = \frac{\left((u-s) - \frac{1}{2}(v - v^R)\ell'(s)\right)^2}{(u-s)^2 + (v - v^R)\left(\frac{v + v^R}{4} - \ell(s)\right)} < 1, \tag{10}$$

where $\ell'(s) = \frac{d\ell(s)}{ds}$. The inequality above can be rearranged as

$$-\frac{1}{4}(v - v^R)(1 + \ell'(s)^2) + \left(\frac{v}{2} - \big(\ell(s) + \ell'(s)(u-s)\big)\right) > 0. \tag{11}$$

The term $\ell(s) + \ell'(s)(u-s)$ represents the *y*-coordinate of the intersection between the tangent line to the upper curved crease at s and the vertical line passing through the vertex V_5 (see Fig. S4a). Therefore, we have $\ell(s) + \ell'(s)(u-s) < \frac{v}{2}$, and the expression $\frac{v}{2} - \big(\ell(s) + \ell'(s)(u-s)\big)$ is always positive. Since $(1 + \ell'(s)^2)$ is also strictly positive, the inequality (11) implies

$$v - v^R < \frac{2v - 4\big(\ell(s) + \ell'(s)(u-s)\big)}{1 + \ell'(s)^2}. \tag{12}$$

Introducing the folding limit $v^R_{min} < v$ as

$$v - v^R_{min} = \frac{2v - 4\big(\ell(s) + \ell'(s)(u-s)\big)}{1 + \ell'(s)^2}. \tag{13}$$

We obtain the valid range $v^R \in (v^R_{min}, v)$ that ensures the existence of a continuous solution. Since relation (13) depends on the parameter s, the folding limit can be expressed more compactly as

$$v^R_{min} = \max_{s\in(0,u_c)} \left(v - \frac{2v - 4\left(\ell(s) + \frac{d\ell(s)}{ds}(u-s)\right)}{1 + \left(\frac{d\ell(s)}{ds}\right)^2} \right). \tag{14}$$

If θ^0 (Fig. S4b) is the angle between the projection of the rule vector **r**(0) (extended from the curved crease towards the cone apex) onto the *xz*-plane and the positive direction of the *x*-axis, we can use relations (4), (5) and (9), to derive the equation for the *xz*-plane projection of the folded lens unit in the form of

$$g(s) = r(s)\sin\theta(s). \tag{15}$$

Since $\tan\theta^0 = b_3/\tilde{u}$, where $\tilde{u} = u\cos\mu + (\frac{v^M}{2} - \ell_o)\tan\mu$ denotes the *x*-coordinate of the vertex V_6 (and V_5) in the folded state of the lens unit, by introducing the parameter $h_0 = b_3 = \sqrt{\left(\frac{v}{2} - \ell_o\right)^2 - \left(\frac{v^M}{2} - \ell_o\right)^2 / \cos^2\mu}$ as the thickness of the folded lens-box (see Fig. S4b) we can obtain

$$\theta^0 = \tan^{-1}\left(\frac{h_0}{u\cos\mu + (\frac{v^M}{2} - \ell_o)\tan\mu}\right). \tag{16}$$

The folding parameter μ can be expressed as a function of v^R by substituting $g(s)$ in relation (3). Hence, for any given unfolded 2D curve crease ℓ, the *xz*-plane projection of the folded geometry, g, must be a function of only one independent folding variable v^R meaning that it has a single DOF. This result enables to describe our curved crease lens unit as a single-DOF mechanism, similar to previous observations [12]. The counterpart relation can be derived for the left-curve.

Upon assuming $\mu = 0$ for all folded states, if the magnitude of $g'(0) = 0$, relation (3) reduces to $\ell'(0) = 0$. This result does not depend on the folding parameter v^R; the left-hand

pattern becomes the reflection of the right-hand pattern, $h_0 = \sqrt{\left(\frac{v}{2} - \ell_o\right)^2 - \left(\frac{v^M}{2} - \ell_o\right)^2}$ and relation (16) reduces to

$$\theta^0 = \tan^{-1}\left(\frac{1}{u}\sqrt{\left(\frac{v}{2} - \ell_o\right)^2 - \left(\frac{v^M}{2} - \ell_o\right)^2}\right). \qquad (17)$$

Eqs. (4),(9),(15) and (17) addresses the forward problem, i.e., it establishes a relation between $\theta(s)$, the rule segment length $r(s)$ and the *xz*-plane projection of the folded geometry $g(s)$; they are instrumental to determine the folded geometry of the lens-unit from a predefined function ℓ of the planar curve crease and given geometric parameters, ℓ_o, u, u_c, and v. They refer to the lens-unit only, one constituent of our lens-box pattern. In the next section we examine the folded geometry of the second constituent, the waterbomb connector.

2.2 Characterization of the '*Waterbomb Connector*'

The 'waterbomb connector' in our lens box pattern plays a twofold role. First, it contributes to the overall pattern folding by acting as a rigid-foldable mechanism; second, it interfaces two lens units. If we consider two waterbomb connectors one on the right hand side of the lens unit and the other on left hand side of the lens unit (Fig. S1), we obtain the governing mechanism of the lens-box pattern which possesses a lock configuration, beyond which no further folding is possible due to the contact of the waterbomb panels; at this state the waterbomb connector is flat-folded. Since the lens-box unit must be developable, rigid-ruling foldable, and lockable, we need to guarantee geometrical congruence between the lens unit and the adjacent waterbomb connectors across the entire rigid folding, from the unfolded flat state to the lock configuration. Fig. S6a illustrates the incremental transition of a typical 2D unfolded waterbomb connector unit to its final 2D flat-folded state.

It has been shown that the general waterbomb pattern consisting of a series of degree-6 vertices (where six creases meet), is rigidly foldable with multiple DOFs, which can be reduced to one if the pattern folds symmetrically with respect to its bisecting *yz*-plane (Fig. S6a) [13]. In contrast, our waterbomb pattern consists of a single cell only which is not laterally confined by

other waterbomb units. Hence upon assuming symmetric folding as discussed later in section S5 rigid-foldability, our connectors have 2-DOF.

2.2.1 Flat-Foldability

The flat-foldability of the waterbomb connector ensures the existence of the lock state for our lens-box origami, and guarantees the smooth connection between two adjacent lens-box units. To assess the local flat-foldability of a single vertex regardless of the mountain-valley assignment (*MV* assignment), we can resort to the *Kawasaki's theorem*, a useful tool to assess the flat-foldability of an origami vertex [14]. Kawasaki's theorem also implies the existence of an even number of creases in a flat-foldable origami, i.e., the parity of creases in a flat-foldable pattern.

Another useful tool to assess the flat-foldability of an origami crease pattern is the *Maekawa's Theorem*, which states that the difference between the number of mountains (*M*) and valleys (*V*) at every vertex equals to 2, i.e., $M - V = \pm 2$ [15, 16]. None of these theorems, however, provide sufficient information to assess the flat-foldability of a multi-vertex mountain-valley crease pattern. For example, a general rigid-foldable waterbomb pattern is not necessarily flat-foldable while satisfying both the conditions mentioned above, an example being a pattern with sector angle $\gamma = \pi/2$. Here, to attain a flat-foldable waterbomb connector, we use an inverse approach. We first assume the existence of a flat-folded state, and then obtain the constraint relations among the unfolded geometrical parameters that avoid penetration of panels or edges in the flat-folded state. We can show that if a flat-folded state exists, any intermediate folding states also exist as demonstrated in the next section. Fig. S6a shows a schematic of the folding process of a representative flat-foldable waterbomb crease with vertices specified in relation to the lens unit.

To derive the flat-foldability constrains of the waterbomb connector, we first define the pertinent notation. We use V_i to denote the vertices of the waterbomb connector, and $\hat{f}(.)$ to refer to the folding mapping of any attribute (.), such as vertices, crease points, and surfaces, in the '*lock state*' of the lens-box, occurring when the waterbomb connector reaches its final '*flat-folded state*'. The notation $f(.)$ is preserved for referring to all other intermediate folding configurations. To specify the folding parameters in the lock configuration, the superscript $\star$ is used. For example, in Fig. S6a, $\|f(V_5)f(V_6)\| = v^R$ for the partially folded configuration but in the flat-folded state

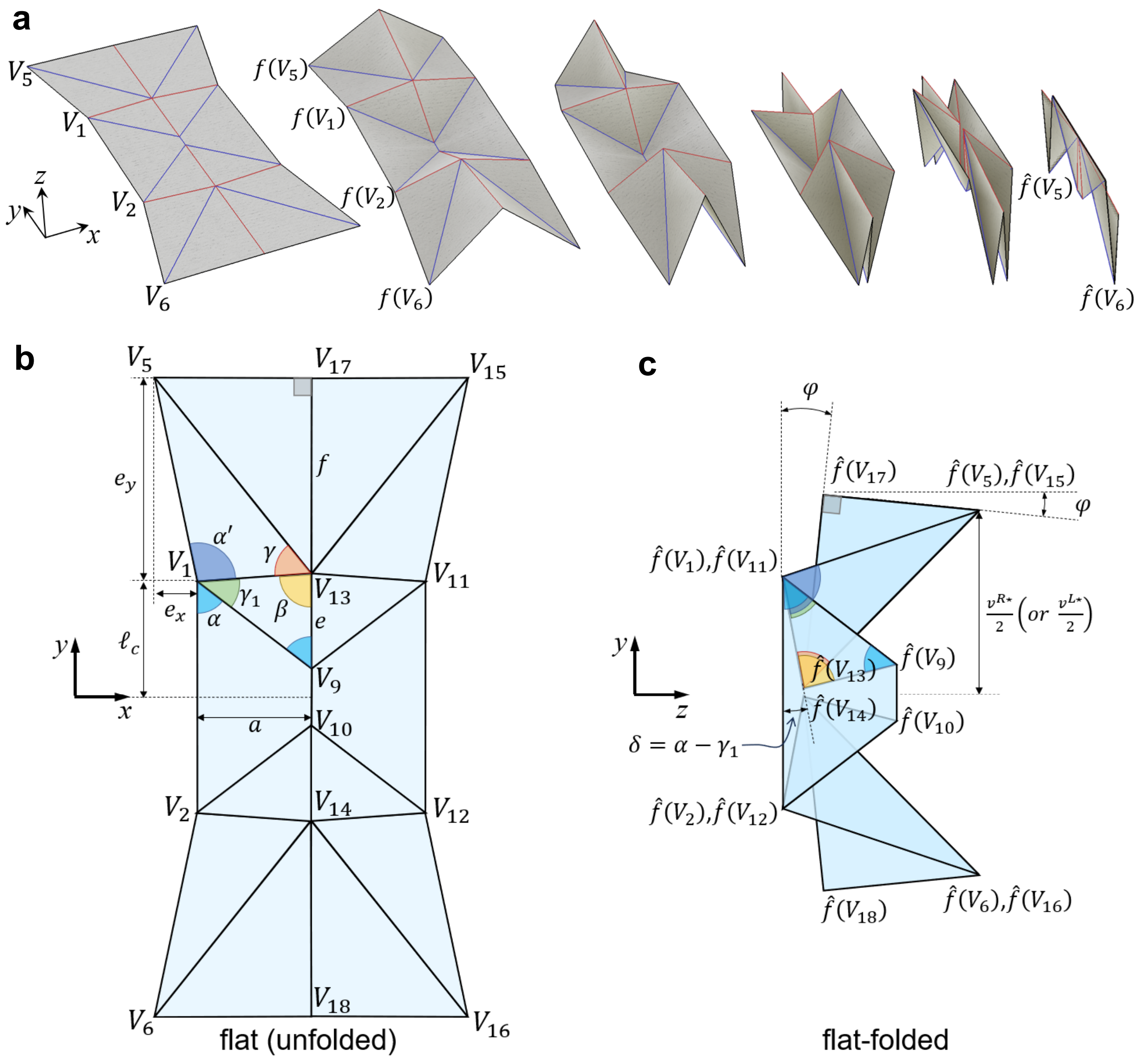

Fig. S6. Geometrical parameters for flat-foldability of waterbomb connector unit. (a) Sequential folding of waterbomb connector. Geometry of flat-foldable waterbomb in (b) unfolded, and (c) flat-folded state. $\hat{f}(.)$ refers to the folding mapping of any attribute (.) in the lock configuration. Superscript ^{R} refers to the geometrical parameters of the right-hand-side waterbomb connector and ^{L} to the left one.

$\|\hat{f}(V_5)\hat{f}(V_6)\| = v^{R\star}$. Additionally, we use κ_I to denote the first principal (global) curvature of the given surface (along the arc-length s in our lens-box pattern) and κ_{II} as its second principal (global) curvature. We also denote the radius of the first principal global curvature by R_{GI} and that of the second one with R_{GII}, and reserve the notation R_c to denote the local radius of curvature for the lens unit.

Now, consider the unfolded waterbomb connector with its geometric parameters in its unfolded state (Fig. S6b). We introduce the following geometrical parameters of the unfolded state. $f = \|V_{13}V_{17}\|$, a is the horizontal distance between V_1 and V_9, e_x is the horizontal distance between V_1 and V_5, and e_y the vertical distance between V_1 and V_5. We also introduce the angles: $\alpha = \angle V_2V_1V_9$, $\beta = \angle V_9V_{13}V_1$, $\gamma = \angle V_1V_{13}V_5$, $\gamma_1 = \angle V_9V_1V_{13}$, and $\alpha' = \angle V_{13}V_1V_5$. The two parameter e_x and e_y can be calculated using the geometry of the lens unit shown in Fig. S4 for the right waterbomb connector where $e_x^R = u_c - u$ and $e_y^R = \frac{v}{2} - \ell_c^R$. (We will later show that for our inversely designed units, the left connector unit has $e_x^L = u^*$ and $e_y^L = \frac{v}{2} - \ell_c^L$). To continue, we drop the superscript ^{R} and ^{L} to derive general relations. We also introduce the parameter δ to measure the angle $\delta = \alpha - \gamma_1$ and denote the angle that the vector $\overrightarrow{\hat{f}(V_{13})\hat{f}(V_{17})}$ makes with the positive y-axis in the flat-folded configuration with φ as shown in Fig. S6c. The sign of the angle φ determines the sign of the global curvature $\kappa_{II} = 1/R_{GII}$ (see Fig. S7): $\varphi > 0$ if the vector $\overrightarrow{\hat{f}(V_{13})\hat{f}(V_{17})}$ locates in the first quadrant of the 2D *zy*-coordinate, and $\varphi < 0$ if it locates in the second quadrant. We can use the geometry of the locked waterbomb connector and obtain a relation that gives φ for any prescribed curvature radius R_{GII} as $|\varphi| = \tan^{-1}\left(v^{R\star}/\left(2\sqrt{{R_{GII}}^2 - {\ell_0}^2} \mp 2h_0\right)\right)$. In this relation, the upper sign in ($\mp$) relates to the case when the second curvature is convex ($\kappa_{II} > 0$) and the lower sign when it is concave ($\kappa_{II} < 0$).

With reference to the geometry of the unfolded connector given in Fig. S6b, we can obtain the angle α' as $\alpha' = \frac{\pi}{2}\left(2 + \frac{e_x}{|e_x|}\right) - \tan^{-1}\left(\frac{e_y}{e_x}\right) - \gamma_1 - \alpha$, and relate the lock leg distance $v^{R\star}$ (or similarly $v^{L\star}$) to δ and α' through

$$v^{R\star} = 2\ell_c - 2\sqrt{{e_x}^2 + {e_y}^2}\cos(\alpha' + \delta). \quad (18)$$

Upon rearranging relation (18) and using our above-mentioned relation for the angle α', we can calculate γ_1 as a function of the geometric parameters of the lens unit in the form of

$$\gamma_1 = \frac{\pi}{4}\left(2 + \frac{e_x}{|e_x|}\right) - \frac{1}{2}\tan^{-1}\left(\frac{e_y}{e_x}\right) - \frac{1}{2}\cos^{-1}\left(-\frac{v^{R\star} - 2\ell_c}{2\sqrt{{e_x}^2 + {e_y}^2}}\right). \tag{19}$$

For any assumed value of δ, we can calculate the geometrical length $a > a_0 > 0$, with a_0 being the minimum allowable physical length that can be realized by assuming a physical paperboard folding (here assumes to be ~ 4 mm), by solving the equation

$$\left((e_x + a)\cos\varphi - f\sin\varphi + \frac{a\sin\delta}{\sin\beta}\right)^2 + \left((e_x + a)\sin\varphi - f\cos\varphi + \frac{a\cos\delta}{\sin\beta}\right)^2 - {e_x}^2 - {e_y}^2 = 0 \tag{20}$$

where $f = e_y - a\cot\beta$ and $\beta = \pi - \delta - 2\gamma_1$. To avoid overlap between the edges of the folding panels and the protruding panels that extend beyond the folded lens unit, we apply the following constraints that prevent

(*I*) protrusion of the waterbomb panels beyond the surface of the lens unit:

$$\delta \geq 0, \tag{21}$$

(*II*) overlap between the two folded vertices $\hat{f}(V_{13})$ and $\hat{f}(V_{14})$:

$$\ell_c \geq \frac{a}{\sin\beta}\cos\delta, \tag{22}$$

(*III*) penetration of the creases $\overline{\hat{f}(V_{13})\,\hat{f}(V_5)}$ and $\overline{\hat{f}(V_{13})\,\hat{f}(V_{15})}$ into the crease $\overline{\hat{f}(V_{13})\,\hat{f}(V_9)}$ in the lock configuration (Fig. S6c):

$$\beta \geq \gamma, \tag{23}$$

(*IV*) panel overlap between two connecting waterbomb units: $\angle V_{13}V_5V_{17} \geq \angle V_{13}V_5V_1$ that can in turn be written in terms of the other geometrical variables:

$$\gamma \geq \frac{3\pi}{4} - \frac{1}{2}(\alpha' + \beta), \tag{24}$$

where $\gamma = \sin^{-1}\left(\sqrt{\frac{{e_x}^2 + {e_y}^2}{f^2 + (a + e_x)^2}}\sin\alpha'\right)$, and

(VI) overlap between two adjacent folded lens units along their sides (y-axis) when $\varphi < 0$, i.e., $\kappa_{II} < 0$ as shown in Fig. S7a and c:

$$|\varphi| \leq \sin^{-1}\left(\frac{v^R - 2\ell_c}{2\sqrt{{e_x}^2 + {e_y}^2}}\right). \tag{25}$$

For symmetric concave units we should additionally verify $|\varphi| \leq \sin^{-1}\left(\frac{v^R - 2\ell_0}{v - 2\ell_0}\right)$. Four different lens-box tessellation patterns may arise depending on the sign of the two principal curvatures $\kappa_I = 1/R_{GI}$ and $\kappa_{II} = 1/R_{GII}$ as illustrated in Fig. S7. Generally, we find it more challenging to obtain the solution of the inverse problem introduced in sections 4 and 5 for the case $\kappa_{II} < 0$ (or in other words $\varphi < 0$, see for example, Fig. S7a and c) than for the case $\kappa_{II} > 0$ (Fig. S7b and d). One reason is the existence of the additional constraint (VI) which constrains further our search space.

The analysis above has determined the constraints we must apply to our parameters to attain flat-foldability. The following addresses the remaining property: the rigid-foldability of the crease pattern.

3 Rigid-foldability

In general, *rigid-foldability* describes the property of an origami pattern to fold through the sole rotation of its creases without facet deformation. While the theory of rigid-foldability for a single vertex exists [17] and reported in the next section, the rigid-foldability of a general multi-vertex origami crease pattern is NP-hard (non-deterministic polynomial-time hardness) [18]; there exist no theory or rule, similar to the single vertex case, to assess the rigid-foldability (or rigid-ruling foldability) of a general multi-vertex crease pattern. Here, to assess the rigid-ruling foldability of the general lens-box pattern, we first show that our individual vertices are rigid-foldable, and then, by obtaining the configurational space of the waterbomb connector unit we develop the pertinent theory and prove that a single lens-box unit is rigid-ruling foldable. Finally, we resort to rigid folding simulations to illustrate that our lens-box pattern tessellation is also rigid-ruling foldable.

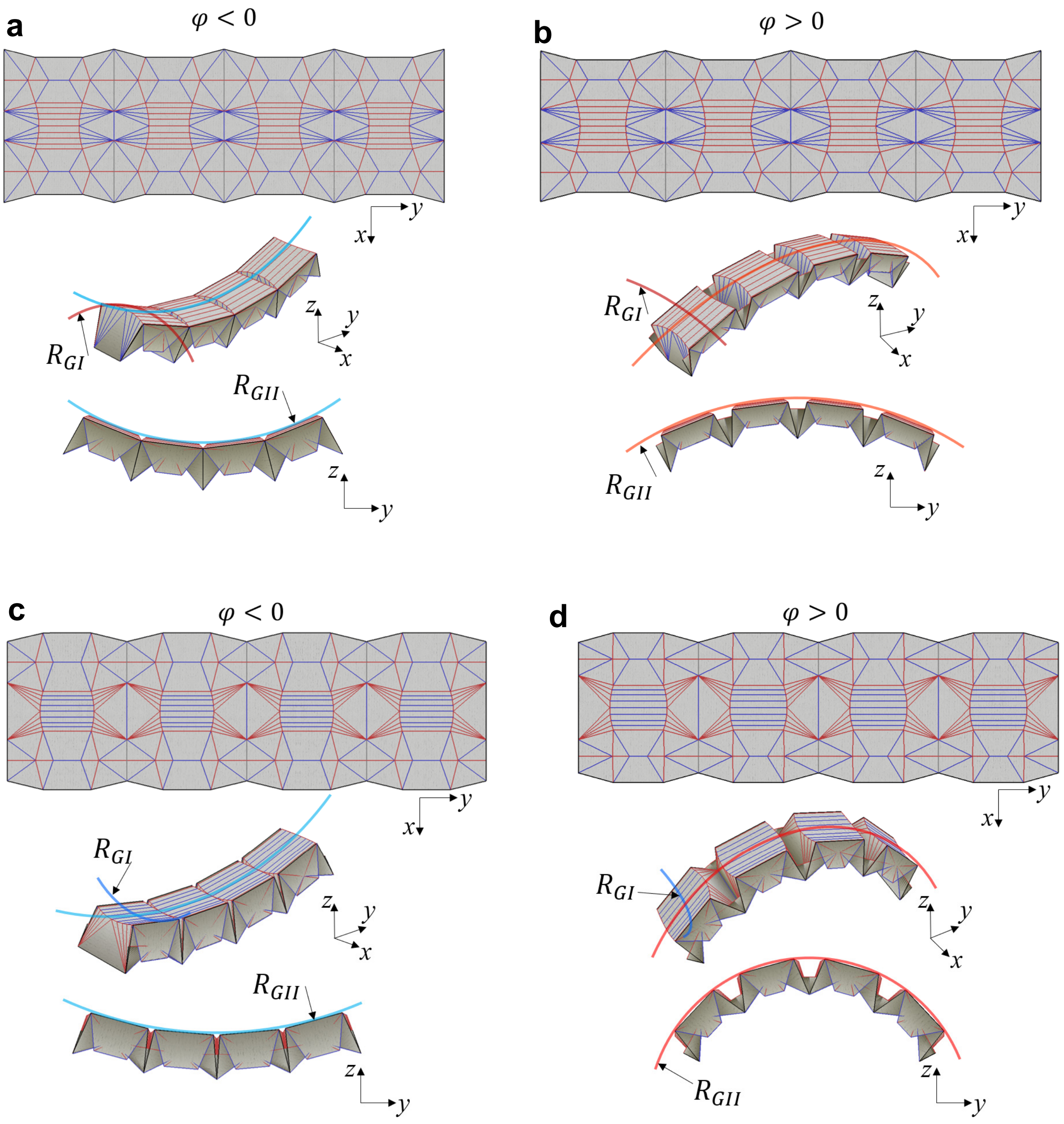

Fig. S7. Lens-box tessellation embeddings of double-curvature surfaces. Four different examples of symmetric lens-box tessellation patterns conforming four possible combinations of double curvature surfaces for $\kappa_I > 0$ & $\kappa_{II} < 0$ (a), $\kappa_I > 0$ & $\kappa_{II} < 0$ (b), $\kappa_I > 0$ & $\kappa_{II} < 0$ (c) and $\kappa_I > 0$ & $\kappa_{II} < 0$ (d) in their unfolded state (top) and locked state (bottom). The first (principal) global curvature is $\kappa_I = 1/R_{GI}$ and the second global (principal) curvature $\kappa_{II} = 1/R_{GII}$. Parameters of the examples illustrated above, $R_{GI} = R_c$, $v = 60$ and $v^R = v^L = v/2$, and the rest of the dimensions (in mm) are (a) $R_c = 50$, $u = 8, u_c = 12.48, \ell_0 = 10.94$, $\delta = 10.85°$ and $\varphi = -7°$; (b) $R_c = 50$, $u = 8, u_c = 12.48, \ell_0 = 10.94$, $\delta = 10.85°$ and $\varphi = +7°$; (c) $R_c = 40$, $u = 14.2, u_c = 9.86, \ell_0 = 13.5$, $\delta = 37°$ and $\varphi = -5°$; (d) $R_c = 40$, $u = 16, u_c = 12.92, \ell_0 = 14.3$, $\delta = 25°$ and $\varphi = +15°$. Models of rigid folding simulations are generated with Freeform software [19].

3.1 Isolated Single-Vertex Fold

We summarize first the necessary conditions ensuring the rigid-foldability of a single vertex and focus on the isolated vertices of our pattern as shown in Fig. S8. Therein are also visualized four typical vertices of the lens-box unit with their sector angles α_i, edges e_i, dihedral folding angles ρ_i and rotation transformation matrices $R_{e_i}(\rho_i)$. A crease pattern with only a single multi-valent vertex (single-vertex fold) can be rigid-foldable if and only if the pattern contains *bird's foot* [20], i.e., if it contains either a 'tripod' or a 'cross' together with another crease that has opposite *MV*-parity than the tripod/cross. A pattern has a mountain (or valley) tripod if there exist three mountain (or valley) creases e_1, e_2, e_3 (not necessarily contiguous) in counterclockwise order with the property that $0 < \angle(e_i, e_{i+1}) < \pi$ for $i = 1,2,3$ [20]. A pattern has a mountain (or valley) cross if there exist four mountain (or valley) creases e_1, e_2, e_3, e_4 (not necessarily appearing together in sequence, e.g., between two creases e_1 and e_2 there could be other creases) in counterclockwise order where e_1 and e_3 form a straight line, as do e_2 and e_4.

It is known that the DOF of a single *n*-valent vertex is $n - 3$. Therefore, V_{13} is a degree-6 rigid origami vertex with general 3-DOF; however, upon assuming the symmetry of folding, it reduces to a 2-DOF [21]. V_9 is a degree-4 vertex with a single DOF. For both vertices one may find a *bird's foot* meaning that they are rigid-foldable. Combination of the pattern made of vertex V_9 and vertex V_{13} which are linked through the edge e_1 describes the isolated waterbomb connector, a pattern that folds symmetrically with 2-DOF.

We now study the rigid-foldability of two special vertices V_5 and V_1 which share three edges with the curved crease lens unit. We first assume the regions bounded by rule segments as rigid panels comprising the lens unit. This suggests that each ruling line can be imagined as a crease. For vertex V_5, rulings are of a valley-crease type (Fig. S8 top left) as shown by grey color edges in Fig. S8 (top right) with two representative ruling creases e_{14} and e_{17} (S8 bottom). With this simplification, V_5 can be treated as an ordinary degree-*n* ($n \geq 7$) rigid origami vertex with $(n - 3)$-DOF. Next, we focus on vertex V_1. Under the assumption of isometric mapping and of an infinite number of rule segments for the cone area, the fold line e_{12} (which is part of the curved crease) becomes tangent to the curve ℓ at vertex V_1. This enables us to obtain the sector angles α_{10} and α_9 as a function of $\ell(s)$ and treat vertex V_1 as ordinary degree-5 origami vertex with 2-DOF.

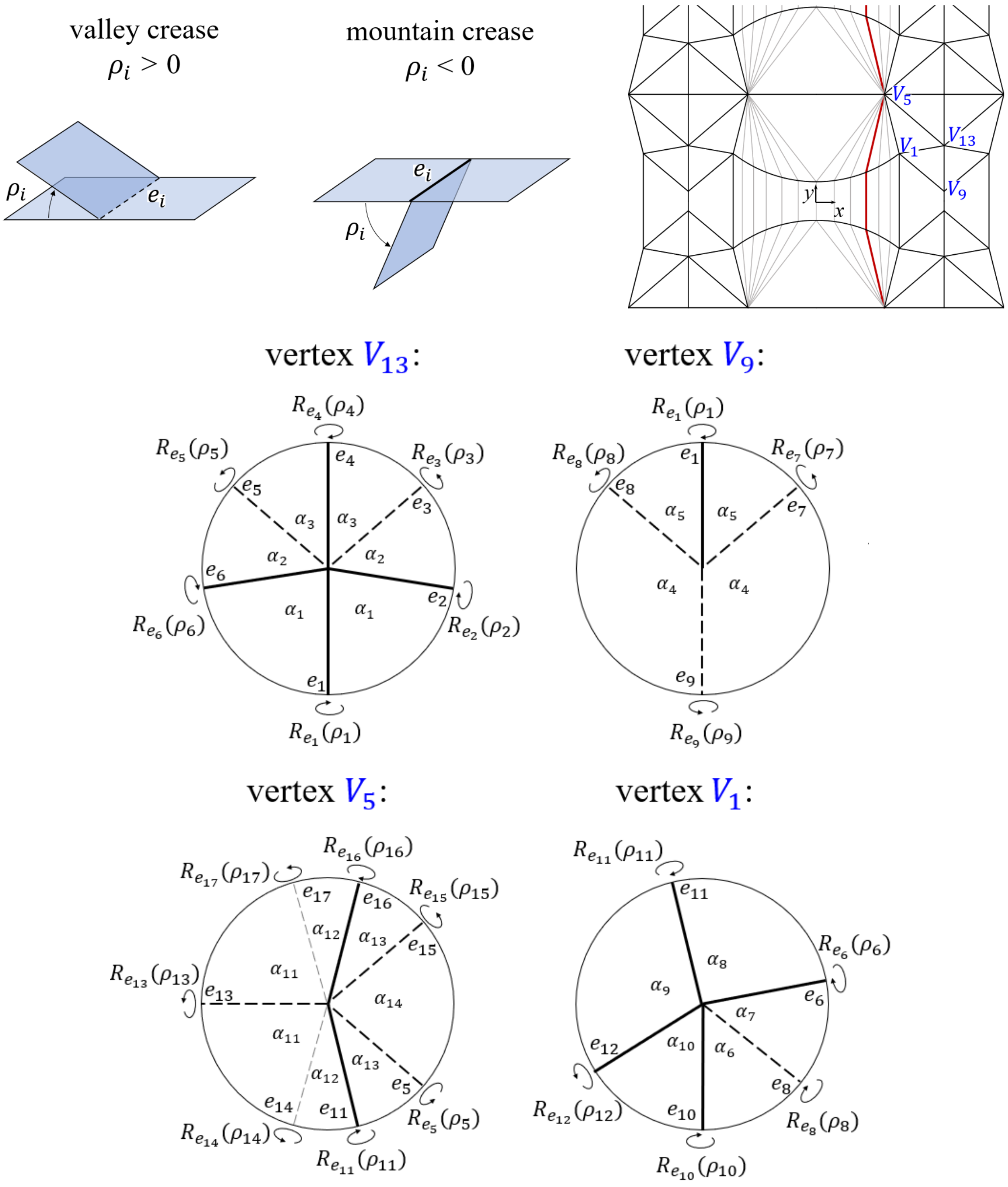

Fig. S8. Rigid-foldability of individual vertices in waterbomb connector. Isolated vertices shown with their mountain valley assignment representing the orthogonal rotational transformation matrix $R_{e_i}(\rho_i)$. All four specified vertices include a *bird's foot* signifying that they are rigid-foldable.

As can be observed in the isolated vertex representation of Fig. S8 (middle), V_5 and V_1 include a *bird's foot* which implies that they are also rigid-foldable.

While the above proves the rigid-foldability of the individual vertices, it is not sufficient to prove the rigid-foldability of a multi-vertex pattern comprised of rigid-foldable vertices. Additional conditions are required as discussed next.

3.2 Conditions for Multi-Vertex Fold Pattern

For a specific pattern, we can assess the rigid folding motion through the concept of isometric folding [17, 18] as follows. Upon denoting $R_{e_i}(\rho_i)$ as the orthogonal rotational matrix that rotates $\mathbb{R}^3$ about the line containing crease e_i by the angle ρ_i, the necessary and sufficient condition for rigid-foldability of a single vertex V_i containing crease lines $e_1, \dots, e_n$ can be written as

$$\prod_{i=1}^{n} R_{e_i}(\rho_i) = \mathcal{F}(\rho_1, \dots, \rho_n), \tag{26}$$

where $\mathcal{F}(\rho_1, \dots, \rho_n) = I_3$, is the 3×3 identity matrix, as shown by Belcastro and Hull [17]. The orthogonal matrix $R_{e_i}(\rho_i)$ in (26) can be obtained as

$$R_{e_i}(\rho_i) = \begin{bmatrix} 1 & 0 & 0 \\ 0 & \cos\rho_i & -\sin\rho_i \\ 0 & \sin\rho_i & \cos\rho_i \end{bmatrix} \begin{bmatrix} \cos\alpha_i & -\sin\alpha_i & 0 \\ \sin\alpha_i & \cos\alpha_i & 0 \\ 0 & 0 & 1 \end{bmatrix}, \tag{27}$$

where the sector angle α_i specifies the angle between two crease lines e_i and e_{i+1}. By expanding the rigid-foldability condition of a single vertex, the necessary condition for a multi-vertex rigid origami pattern Σ that is homeomorphic to a hole-free disk reduces to satisfying the constraint Eq. (26) for all interior vertices (excluding the vertices on the boundary) of the pattern Σ [18, 22]. Assuming that our folding is sufficiently small, and the panels cannot self-intersect by folding from the unfolded flat state (where $\rho_i = 0$ for all *i*), then Eq. (26) is also sufficient to ensure rigid-foldability [18].

Eq. (26) can be used to obtain the relations between the dihedral angles, describe rigid folding, and define the *configuration space C* of the rigidly foldable pattern. Eq. (26) can also be used to simulate rigid folding for example in the *Freeform* origami software [19]. Computational

simulation of a given crease pattern is currently the only option to assess the rigid-foldability of a multi-vertex crease pattern. This tool is useful only for understanding the rigid-foldability of a pattern when all sector angle parameters (i.e., α_i) are known. On the other hand, it cannot be used to assess the rigid-foldability of a general pattern with unknown sector angles. For our general lens-box pattern, therefore, we need to resort to an alternative strategy as follows. We first describe the configurational space of the flat-foldable waterbomb connector unit, and then, we prove that connecting a rigidly flat-foldable waterbomb connector to a rigid-ruling-foldable lens unit preserves rigid-foldability. With this approach, we can then resort to rigid-folding simulations to show that our general pattern is rigid-foldable with rigid-ruling foldable curved creases.

3.3 Configuration Space of the Waterbomb Connector

To describe the configuration space of our isolated waterbomb connector unit we need to study the rigid folding motion of the vertex V_{13} and V_9. The equation of motion can be derived from the relations between the dihedral angles (i.e., all ρ_i) using (26). To simplify the definition of the configuration space of our waterbomb connector unit, we assume symmetric folding with respect to a plane passing through edges e_1 and e_4 (see vertex V_{13} in Fig. S8). This assumption reduces the DOFs from 3 to 2 as previously shown [21]. For our given *MV* assignment, only one deformation mode (see Fig. S9a) exists and can be obtained using Eqs. (26) and (27) as

$$\tan\frac{\rho_1}{2} = \frac{\sin\beta\sin\gamma\sin(\beta+\gamma)\cos\rho_3 + \cos\gamma\left(\sin\beta\cos(\beta+\gamma) - \cos\beta\sin(\beta+\gamma)\cos\rho_2\cos\rho_3\right) + \cos\beta\left(\sin\gamma\cos(\beta+\gamma)\cos\rho_2 + \sin(\beta+\gamma)\sin\rho_2\sin\rho_3\right)}{\sin\gamma\cos(\beta+\gamma)\sin\rho_2 - \sin(\beta+\gamma)\left(\cos\gamma\sin\rho_2\cos\rho_3 + \cos\rho_2\sin\rho_3\right)}, \tag{28}$$

which was previously obtained [21]. Relation (28) represents the configuration space of our 2-DOF symmetric waterbomb connector units as illustrated in Fig. S9b for a given set of sector angles $\beta = 80°$ and $\gamma = 50°$. Upon assuming the face shown in Fig. S9a-top as the top side of the paper, for $\rho_3 \in [0, \pi]$, the pattern folds with *MV* assignment matching the partially folded pattern shown in Fig. S9a-bottom. However, the range of $\rho_3 \in [-\pi, 0]$ pertains to the case where the *MV* assignment is reversed. Fig. S9c visualizes one of the possible rigid folding kinematics of the shown pattern in Fig. S9a as five folding sequences beyond the one shown in Fig. S9a-bottom to its fully flat-folded state. As previously discussed [21], all rigid folding motions obey reflection

symmetry about the plane passing through e_1 and e_4 edge; Farnham et al. [21] argued that it is possible to find 1-DOF slices of the configuration space where a linear relationship exists between some folding angles. They obtained one of such relations for degree-6 rigid origami vertex with symmetric folding using the *parallel pleat transform* technique and parametrization with modified Weierstrass substitution $\tan(\rho_i/4)$ as

$$\tan\frac{\rho_1}{4} = \frac{1-\tan\left(\frac{\gamma}{2}\right)}{1+\tan\left(\frac{\gamma}{2}\right)}\tan\frac{\rho_4}{4}, \tag{29}$$

$$\tan\left(\frac{\rho_2}{2}-\frac{\rho_4}{4}\right) = -\frac{1-\tan\left(\frac{\beta}{2}\right)}{1+\tan\left(\frac{\beta}{2}\right)}\frac{1-\tan\left(\frac{\gamma}{2}\right)}{1+\tan\left(\frac{\gamma}{2}\right)}\tan\frac{\rho_4}{4}, \tag{30}$$

$$\tan\frac{\rho_3}{2} = -\frac{\sin\left(\frac{\beta}{2}\right)\sin\left(\frac{\rho_4}{2}\right)}{\cos\left(\frac{\beta}{2}\right)+\cos\left(\frac{\rho_4}{2}\right)\sin\left(\left(\frac{\beta}{2}\right)+\gamma\right)}. \tag{31}$$

Relations (25-27) denote mode-1 of kinematic motion described in [21].

We can also use relation (26) to relate the dihedral angle ρ_5 in vertex V_9 to the dihedral angle ρ_1; alternatively, we can use the existing explicit kinematic equation for degree-4 rigid origami vertex [23] given by

$$\tan\frac{\rho_5}{2} = -\frac{1}{\cos\alpha}\tan\frac{\rho_1}{2}. \tag{32}$$

The rigid folding motion obtained through Eqs. (29-31) gives a smooth 3D curve on the plotted configuration surface Fig. S9b. To assess the rigid folding motion described by this curve, we focus on the half of the curve lying on the configuration surface plot within the range of $\rho_3 \in [0,\pi]$, which corresponds to the *MV* assignment of Fig. S9a-bottom. Furthermore, we divide this curve into two parts: The initial part is represented with a blue solid line and collects all configurations between the unfolded state ($\rho_4 = 0$) and the configuration when the two triangular panels $V_{13}V_{17}V_5$ and $V_{13}V_{17}V_{15}$ come into contact ($\rho_4 = -\pi$), illustrated by a red dot (corresponds to the folding configuration ② in Fig. S9c). Thus, the initial part covers the range $\rho_4 \in [-\pi, 0]$.

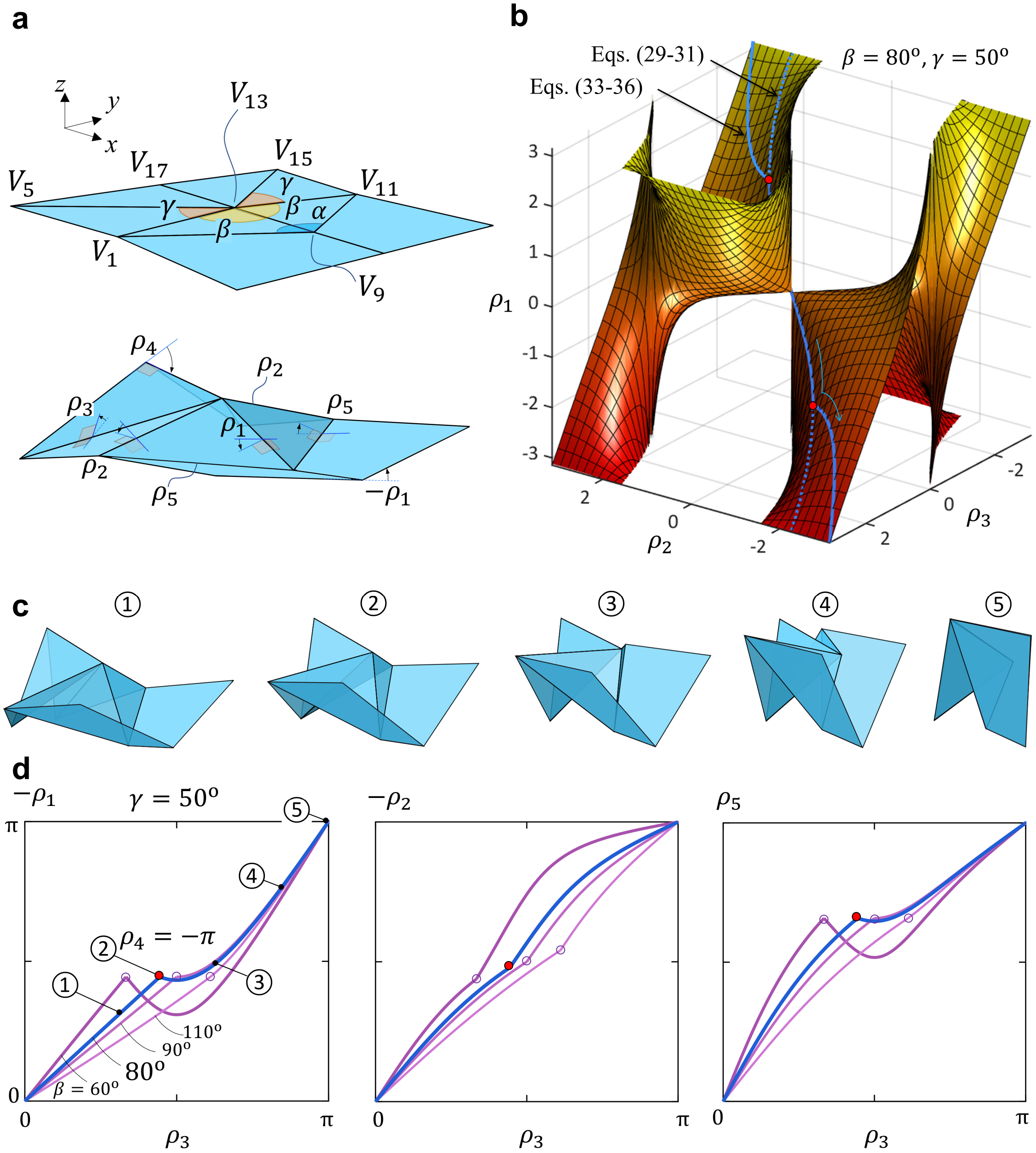

Fig. S9. Full-range configuration space of 2-DOF waterbomb connector unit. (a) Schematic of half of unfolded waterbomb connector unit showing sector angles and vertices (top) with its partially folded state showing its dihedral angles (bottom). (b) Configuration space of waterbomb connector unit for given geometry with sector angles β and γ. Smooth blue curve illustrates the 1-DOF slice of the configuration space using parametric Eqs. (29-31) for the region between the unfolded configuration and the red dot configuration (configuration ② in (c)) where the two triangular panels $V_{13}V_{17}V_5$ and $V_{13}V_{17}V_{15}$ come into contact (solid-line), and beyond the red dot configuration (dash-line) which associates with unfeasible kinematics. The second branch of the solid-line curve, beyond the red dot configuration, represents the configurations obtained using kinematic Eqs. (33) and (36). (c) Sequential reconfiguration of the half of a typical waterbomb unit with symmetric folding from partially folded state to its fully flat-folded state. (d) Planar views of similar 3D configuration curves (only for the feasible kinematic, shown by solid-line) with the relation between ρ_5 and ρ_3 for four geometric parameters $\beta = 60°, 80°, 90°, 110°$ when $\gamma = 50°$.

The second part of the curve represented with a blue dash line collects configurations beyond the red dot, i.e., when $\rho_4 < -\pi$: This part of the curve, however, cannot represent a feasible kinematic as any configuration in his range requires the penetration of the two triangular panels $V_{13}V_{17}V_5$ and $V_{13}V_{17}V_{15}$. The planar views of similar 3D curves (only for the feasible range $\rho_4 \in [-\pi, 0]$) with an additional plot revealing the relation between ρ_5 and ρ_3 is illustrated in Fig. S9d for four different connector unit geometries specified by two sector angles $\gamma = 50°$, and $\beta = 60°, 80°, 90°, 110°$. In Fig. S9d, when $\beta \neq 80°$ we use unfilled circles to show the initial panel contact configuration. Note that the illustrated curves (with solid lines) beyond the red dot in Fig. S9b and d and the folding states beyond the configuration ② in Fig. S9c, i.e., ③, ④ and ⑤, belong to a separate folding kinematic motion as we explain in the next section.

To further investigate the kinematic motions described by above-written relations (25-27) when the sector angles β and γ varies, we plot the dihedral folding angles ρ_1, ρ_2, ρ_3 and ρ_5 with respect to ρ_4 for the given set of constant sector angles in Fig. S10a and b, respectively. These results show that if $\gamma < \pi/2$ and $\gamma + \beta < \pi$ our pattern is rigidly foldable as its dihedral folding angles are monotonically increasing or decreasing. When $\gamma > \pi/2$, however, the dihedral angles ρ_1 and ρ_5 change their sign, i.e., *MV* assignment, indicating an incompatible folding motion with our pre-assigned pattern. More importantly, we observed that none of such rigid folding motions can fully flat fold the connector unit. When the dihedral angle ρ_4 folds completely ($\rho_4 = -\pi$: see configuration ② in Fig. S9d and the red dot configuration in Fig. S9b and d), still other dihedral angles fold only partially. To fully fold them, the two triangular panels $V_{13}V_{17}V_5$ and $V_{13}V_{17}V_{15}$ must intersect each other (Fig. S9a). Hence, the blue dash-line in Fig. 9b cannot associates to a feasible kinematic, and the set of Eqs. (29-31) are unable to explain the entire range of rigid folding motion of the waterbomb connector unit. We develop an additional kinematic equation to explain the rigid folding motion beyond the initial panel contact (red dot configuration) in next section.

3.3.1 Folding Kinematics upon Panel Contact

Once two triangular panels $V_{13}V_{17}V_5$ and $V_{13}V_{17}V_{15}$ come into contact, one may assume that these two panels merge together as they cannot penetrate, hence converting the partially folded unit (see Fig. S11a and b) into a non-Euclidean degree-4 origami vertex (forming a convex polyhedral

cone), where the sum of the sector angles between adjacent creases in the folded pattern respects the condition $\sum \alpha_i < 2\pi$ as shown in Fig. S11c.

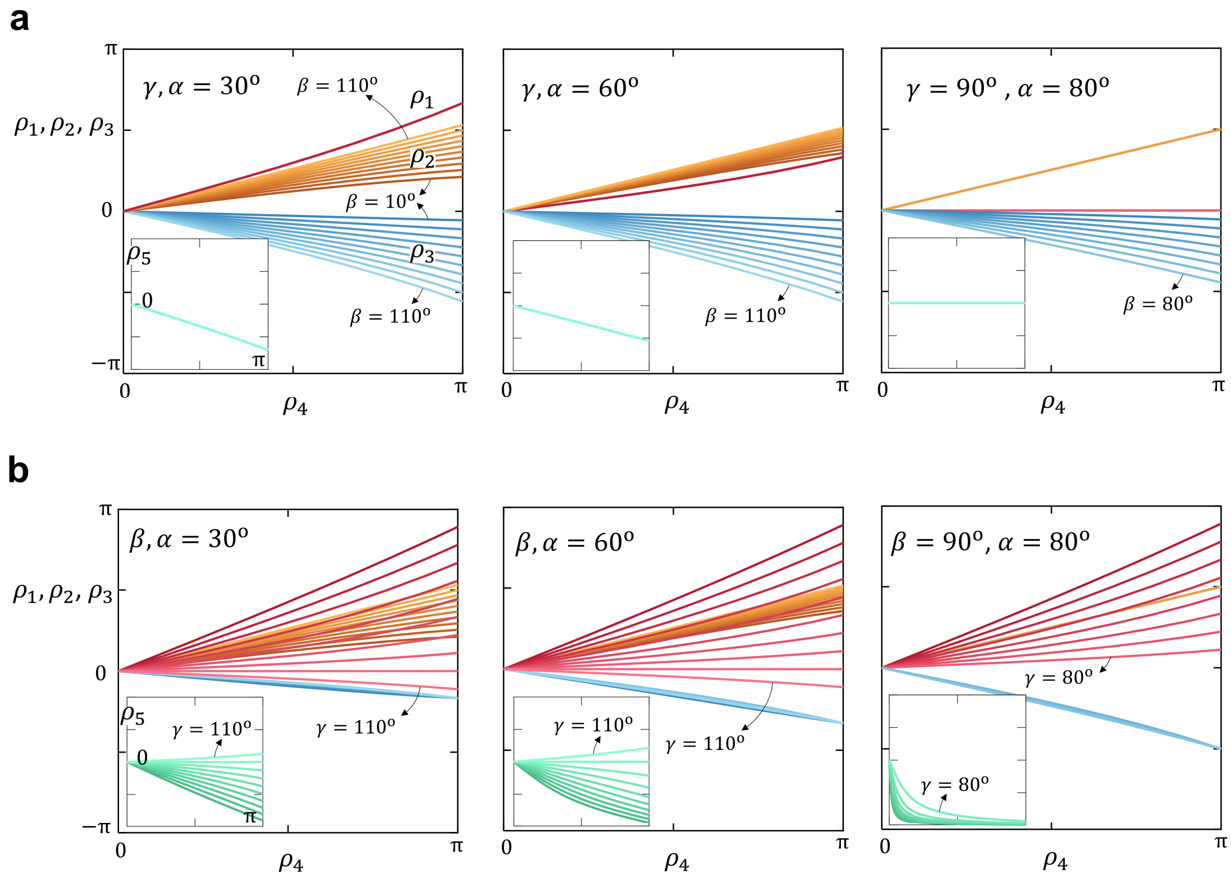

Fig. S10. Configuration space of a 1-DOF symmetric waterbomb before contact. (a) Dihedral angles ρ_1, ρ_2, ρ_3 and ρ_5 with respect to ρ_4 obtained using Eqs. (29-32) for the given set of constant sector angles γ and α when β varies from 10° to 110° in the increments of 10°. (b) Dihedral angles ρ_1, ρ_2, ρ_3 and ρ_5 with respect to ρ_4 obtained using Eqs. (29-32) for the given set of constant sector angles β and α when γ varies from 10° to 110° in the increments of 10°. A reversal of the mountain and valley assignment to obtain dihedral angles is equivalent to changing the sign of the dihedral angles.

The rigid motion of the connector unit beyond the configuration of merged panels can be formulated as follows. Consider the unfolded vertex V_{13} sketched in Fig. S11a and its symmetric folding in Fig. S11b. Upon identifying the two triangles $\triangle f(V_1)f(F)f(V_{11})$ and $\triangle f(V_1)f(G)f(V_{11})$ in the folded configuration in Fig. S11c, we can obtain the relation between $\rho_1 < 0$ (mountain fold) and $\rho_3 > 0$ (valley fold) by writing the distance $\|f(V_1)f(V_{11})\|$ using the

cosine rules as $\|f(V_1)f(V_{11})\|^2 = 2l^2\sin^2\beta(1-\cos(\pi-|\rho_1|)) = 2l^2\sin^2\gamma(1-\cos(2\rho_3))$. Since $1-\cos(2\alpha) = 2\sin^2\alpha$ for any angle α, we obtain

$$\cos\left(\frac{|\rho_1|}{2}\right) = -\frac{\sin\gamma}{\sin\beta}\sin\rho_3. \tag{33}$$

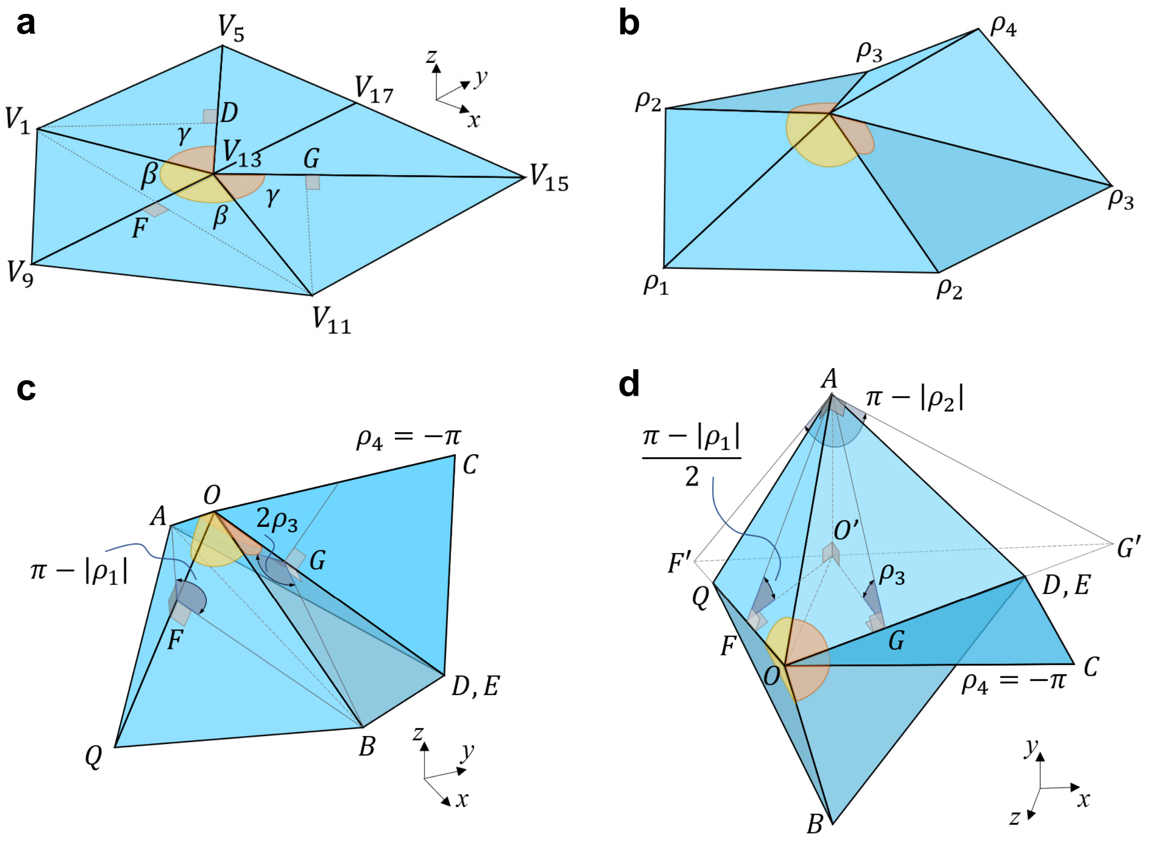

Fig. S11. Geometry of 1-DOF symmetric waterbomb connector after contact. Schematic of vertex V_{13}, of waterbomb connector unit in its unfolded (a) and partially folded (b) states. (c) Folded configuration when $\rho_4 = -\pi$; the panels $V_{13}V_{17}V_5$ and $V_{13}V_{17}V_{15}$ come into contact if $\rho_4 = -\pi$: this schematic is used to obtain the relation between the two dihedral angles $\rho_1 < 0$ (mountain crease) and $\rho_3 > 0$ (valley crease). (d) Necessary geometrical details for the calculation of the dihedral angle of $\rho_2 < 0$ as a function of ρ_3.

To find the relation between the dihedral angles $\rho_2 < 0$ (mountain fold) and ρ_3 we use another view of the folded origami vertex V_{13} sketched in Fig. S11d. Upon denoting the angle $\angle F'f(V_{13})O'$ by q_1 and the angle $\angle G'f(V_{13})O'$ by q_2, and using the cosine rule we can write

$$\|F'G'\|^2 = \|f(V_{13})F'\|^2 + \|f(V_{13})G'\|^2 - 2\|f(V_{13})F'\|\|f(V_{13})G'\|\cos(q_1 + q_2), \quad (34)$$

and

$$\|F'G'\|^2 = \|f(V_1)F'\|^2 + \|f(V_1)G'\|^2 - 2\|f(V_1)F'\|\|f(V_1)G'\|\cos(\pi - |\rho_2|). \quad (35)$$

Since $\|f(V_{13})F'\| = \|\overline{V_{13}V_1}\|/\cos\beta$, $\|f(V_{13})G'\| = \|\overline{V_{13}V_1}\|/\cos\gamma$, $\|f(V_1)F'\| = \|\overline{V_{13}V_1}\|\tan\beta$, $\|f(V_1)G'\| = \|\overline{V_{13}V_1}\|\tan\gamma$, $\cos q_1 = \cos\beta/\|f(V_{13})O'\|$, $\cos q_2 = \cos\gamma/\|f(V_{13})O'\|$, $\sin q_1 = \sin\beta\cos((\pi - |\rho_1|)/2)/\|f(V_{13})O'\|$, $\sin q_2 = \sin\gamma\cos\rho_3/\|f(V_{13})O'\|$ and $\|f(V_{13})O'\| = \sqrt{1 - \sin^2\beta\sin^2((\pi - |\rho_1|)/2)}$, equating (34) and (35), after simplification, yields

$$\cos|\rho_2| = \frac{\sin\left(\frac{|\rho_1|}{2}\right)\cos\rho_3 - \cot\gamma\cot\beta\sin^2\beta\cos^2(\frac{\rho_1}{2})}{1 - \sin^2\beta\cos^2(\frac{\rho_1}{2})}, \quad (36)$$

where ρ_1 is a function of ρ_3 through equation (33).

3.3.2 Kinematic Origin of Folding Difficulty

The two sets of Eqs. (33) and (36) trace a feasible 1-DOF rigid-folding motion of our connector waterbomb unit after the initial panel contact (red dot), corresponding to the second branch in Fig. S9b and to the curve beyond the red dot (and unfilled circles) in Fig. S9d. Our results show that the sector angles $\beta > \pi/2$ yield a strictly monotonic (increasing or decreasing) evolution of all dihedral angles along this path. However, for $\beta < \pi/2$, the dihedral angles ρ_1 and ρ_5 are no longer monotonic: they first change in one direction, then pass through an extremum and reverse.

During the manual folding process, we observed that while a single unit can be folded relatively easily for almost any β, folding a tessellated pattern becomes significantly more difficult for smaller sector angles $\beta < \pi/2$. We associate this increased difficulty with the kinematics of folding an origami pattern, where some creases exhibit a non-monotonic evolution of their dihedral angles, i.e., the folding rate $d\rho_i/d\rho_3$ (with $i = 1, 2, 5$) changes its sign along the motion (see Fig. S9d). At these turning points, ρ_i can no longer be treated as a smooth, globally monotonic function of ρ_3; the configuration corresponding to the extremum is a kinematic singularity when the motion is parameterized by ρ_3. In practice, this means that as ρ_3 continues to change, certain creases must

momentarily “stall” and then reverse rotation, which is difficult to realize through simple manual actuation and tends to promote panel bending or apparent locking.

In a tessellated lens-box pattern, many connector units share panels and must fold compatibly. For $\beta < \pi/2$, every connector must pass through its non-monotonic turning point, meaning that several creases across the tessellation must stall and reverse rotation in a coordinated way. Small mismatches between neighboring units then accumulate, forcing additional panel bending and local deviations from the ideal rigid-folding path, which manifest as apparent locking or “fussiness” during manual folding. We now turn our attention to assessing the rigid-foldability of a full lens-box pattern.

3.4 A Single Lens-Box Unit

To assess the rigid-foldability of the lens-box tessellation, we first study the rigid-ruling foldability of an individual lens-box unit. We combine our understanding about the rigid folding motion of the isolated waterbomb connector unit and that of the lens unit. We assume the ruling lines as creases, and the regions separating them as rigid panels. We start by proving a useful theorem.

Theorem 3.4.1. *If both right and left waterbomb connectors and the lens unit in the lens-box unit $\mathcal{D}$ are rigid-foldable within a given range of folding angles, then the lens-box unit $\mathcal{D}$ is rigid-ruling foldable over the same range of folding angles.*

Proof. We prove the rigid-ruling foldability of the right half of the pattern, acknowledging the left half can be proved similarly. Let us first separate the two waterbomb connectors from the lens unit in the lens-box pattern and assume that the individual units fold symmetrically relative to the symmetry planes of the unfolded unit. We consider the slightly folded right connector and the lens unit independently as shown in Fig. S12, and use the superscript ^{l} to refer to the (detached) vertices if they are on the lens unit and superscript ^{c} if they are on the connector.

Since the connector is rigid-foldable, we can rigidly fold its initially unfolded state by strictly increasing or decreasing the dihedral angle of each crease to an intermediate state where the distance between the two folded vertices V_5^c and V_6^c (or V_{15}^c and V_{16}^c) is $v^R = v^{R*}$, i.e., $\|f(V_5^c)f(V_6^c)\| = v^{R*}$ (see state-I or II in Fig. S12). Similarly, the rigid-ruling foldability of the lens unit implies that it can rigidly fold by monotonically increasing its dihedral folding angles

where the distance between its two folded vertices V_5^l and V_6^l (or V_{15}^l and V_{16}^l) becomes $v^R = v^{R*}$, i.e., $\left\|f\left(V_5^l\right)f\left(V_6^l\right)\right\| = v^{R*}$. Recall we proved the rigid-ruling foldability of the lens unit by showing the existence of the folded geometry via isometric mapping.

Now, consider the partially folded construct of both units in any given configuration where $v^R = v^{R*}$ as shown in Fig. S12. The right edge of the folded lens unit (depicted by red lines) with the line connecting the two folded vertices $f\left(V_5^l\right)$ and $f\left(V_6^l\right)$, and the left edge of the waterbomb connector (depicted by red lines) with the line connecting the two folded vertices $f(V_5^c)$ and $f(V_6^c)$ form respectively two planar isosceles trapezoids of similar geometry and base length v^{R*}. We can connect them from the corresponding side of the trapezoids by rigid rotation of the partially folded units around the y-axis and translation so as to make the folded geometry construct of the lens-box unit $\mathcal{D}$. Since the vertices V_1 and V_2 are origami vertex degree-5 with two DOFs, the newly formed dihedral angles of the V_5V_1 and V_1V_2 edges (and equivalently V_1V_2 and V_2V_6 edges) can attain independent values for any given set of folding angles in the other creases. As the pattern is initially in a flat configuration, it also satisfies the developability constraints.

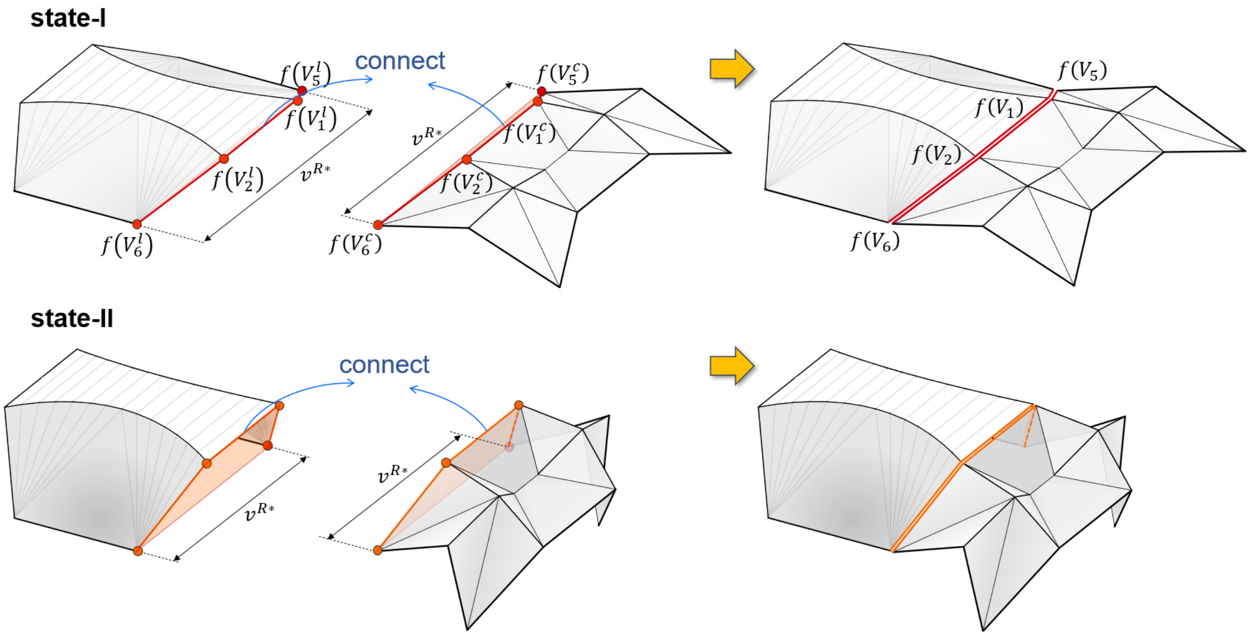

Fig. S12. Connecting a partially folded lens unit to the waterbomb connector unit. Schematic of the rigid-ruling foldable lens unit separated from the symmetric waterbomb connector unit in a slightly folded state, immediately after the unfolded configuration, (state-I), and that of an intermediate partially folded state (state-II). When the distance between its two folded vertices $f(V_5)$ and $f(V_6)$ is v^{R*}, i.e., $\|f(V_5)f(V_6)\| = v^{R*}$, the two units can connect to construct the folded state of a pattern made of both units.

Upon representing the newly formed edge V_5V_1 with vector $\mathbf{e}_{11}$ and the edge V_1V_2 with $\mathbf{e}_{10}$, the angle between the two coincident edge vectors $\mathbf{e}_{10}$ and $\mathbf{e}_{11}$ (see Fig. S8) can be obtained through the inner product $\mathbf{e}_{10}.\mathbf{e}_{11}$ and the relation $\mathbf{e}_{11} = R_{e_{10}}(\rho_{10})R_{e_8}(\rho_8)R_{e_6}(\rho_6)\mathbf{e}_{10}$.Using the trapezoidal shown in Fig. S12, the base length v^{R*} can be obtained as $v^{R*} = \|\mathbf{e}_{10}\| + 2\|\mathbf{e}_{11}\| \sin\left(\cos^{-1}\left(\frac{\mathbf{e}_{10}\cdot\mathbf{e}_{11}}{\|\mathbf{e}_{10}\|\|\mathbf{e}_{11}\|}\right)\right)$. We note that some of the dihedral angles, such as ρ_1 and ρ_{12} (see vertices V_9 and V_1 in Fig. S8), i.e., the folding angle of the curved crease, may not strictly increase or decrease for a certain choice of the sector angles, see for example the results in Fig. S9d for $\beta < \pi/2$.

3.5 Tessellated Lens-Box Crease Pattern

Here, we first assess the rigid-ruling foldability of our one-dimensional (1D) tessellated crease pattern, and then discuss that of the 2D planar tessellation. In the limit of a small folding angle close to the initial unfolded state, the rigid-ruling foldability of the tessellated crease pattern along the lens panel length, i.e., the *x*-direction in Fig. S4, can be easily proven by using the theorem S3.4.1. However, its rigid-foldability for large folding angles cannot be guaranteed by solely using this theorem, as configurations with a large folding angle may yield the intersection of the two end units of a one-dimensionally tessellated pattern. The condition to avoid this in a pattern tessellated by *n* lens-box units along the *x*-axis (see Fig. S13a and b) when it folds from flat to lock configuration is $n|\rho_1(\rho_4 = -\pi)| < 2\pi$. Using relation (29), this condition can be rewritten as

$$n\left|\tan^{-1}\left(-\frac{1-\tan\left(\frac{\gamma}{2}\right)}{1+\tan\left(\frac{\gamma}{2}\right)}\right)\right| < \frac{\pi}{2}. \quad (37)$$

Relation (37) provides the maximum number of units *n* to attain the full range of rigid-ruling foldability as a function of one geometric parameter only, i.e., the sector angle γ.

In contrast to the 1D tessellation along the lens panel length, i.e., *x*-direction, understanding the rigid-ruling foldability of the tessellated crease pattern along the width, i.e., *y*-direction, is not straightforward. To do so, we assume again that our individual lens-box units are rigid-ruling foldable within their full range of motion from the unfolded to the lock state. This implies that for the tessellated pattern we have two states (the unfolded pattern and its lock state)

to inversely design our connector units to flat-fold. The geometrical parameters are computed in the lock configuration by preserving the developability constraint. Although the existence of an intermediate configuration has been attributed to the sufficient condition for the rigid-foldability of the quadrivalent rigid origami vertex [24], unfortunately this is not proved for a general case of a degree-n ($n > 4$) rigid origami vertex. The presence of the lock state in our patterns might be an indication of their rigid-ruling foldability as we experimentally realized that our tessellated pattern in x-direction (see Fig. S4) can be easily folded. This is confirmed by rigid folding simulations of several double-curvature lens-box pattern using the *Freeform* origami software [19, 22] as shown for example in Fig. S13c and Fig. S7. Figure S13c shows three different folding states of a tessellated pattern: a flat pattern, a partially folded state and the lock state.

We now focus on the rigid-foldability of a pattern that tessellates the plane. Proving the rigid-ruling foldability of the general lens-box crease pattern cannot be done through the existing theories as discussed earlier. However, we can do so by resorting on numerical approaches, e.g., *Freeform* origami software [19], upon satisfying the constraint Eq. (26) for all the interior vertices. Figure S13 demonstrates two distinctive examples of our rigid folding simulations of the in-plane tessellation pattern in three different states (unfolded, partially folded and final lock configuration). Figure S13*A* illustrates an inversely designed pattern that upon locking conform to a smooth convex cylinder, while the pattern shown in Fig. S13b conforms into convex cylinder upon locking. These simulation results show that the curved creases remain smooth, i.e., C^2 continuous, during the entire process of folding, and precisely approximating our folded paperboard prototypes shown in Fig. 2 (main manuscript). From these observations, we may conclude that the existence of any non-trivial configuration state (for example, its lock configuration) can prove the rigid-ruling foldability of our tessellated lens-box pattern, although we currently do not have a mathematical proof for our general lens-box tessellation, still an open question to answer.

Previous sections primarily establish the necessary geometrical relations to construct the folded lens unit and the waterbomb connector, addressing the forward problem. The following section tackles a more intriguing question: the inverse design.

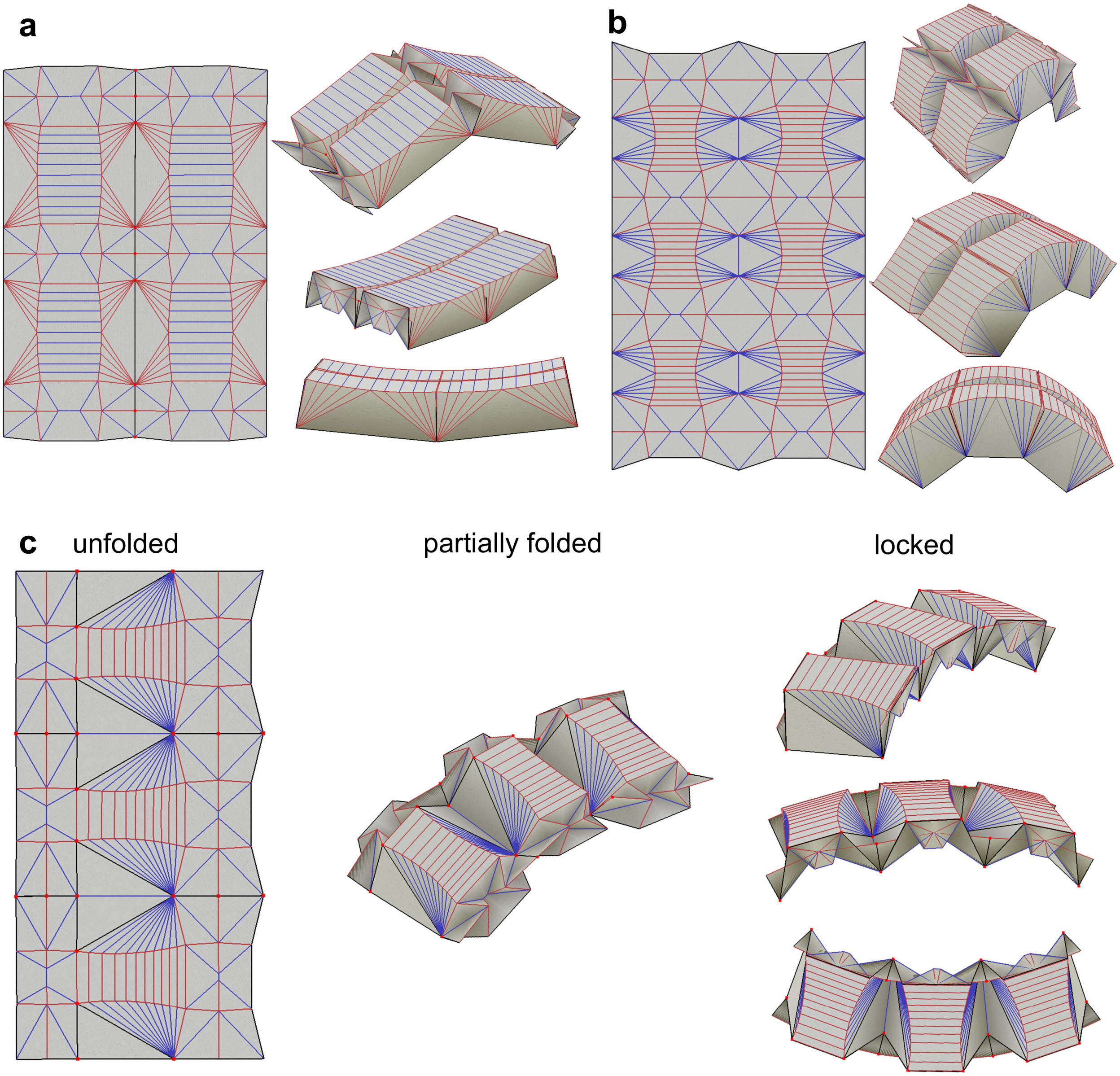

Fig. S13. Rigid folding simulations of lens-box pattern. (a) Inversely designed pattern that in its lock state conforms to a concave arc with constant curvature. (b) Inversely designed pattern that in its lock state conforms to a constant curvature curve, as shown by the paperboard model in Fig. 2a. (c) Inversely designed crease pattern that upon locking conforms to a portion of a sphere hence folding in a surface with double curvature. This model represents the third row in the paperboard sample shown in Fig. 2d. Models created using *Freeform* origami software [19]. Mountain creases in the pattern are shown in blue and valley creases in red.

4 Inverse Problem

With a focus on the geometry our lens unit can take on upon locking, our goal here is to find the projection of $f(M)$ onto the *zx*-plane that conforms to a predefined (on-target) curve $g(s)$ (Fig. S4b) with s being the arc-length parameter. This objective translates into the search of $h(s)$ (bottom of Fig. S4b), the distance between the vertex $f(V_5)$ and the crease point s on the *zx*-plane projection of the locked unit. Here, we do not prove the existence of the solution for a general prescribed curve $g(s)$ with curvature, although our method may be used for any given $g(s)$ that is a planar not-intersecting smooth concave or convex curve. Rather, we solve the inverse problem for a specific concave or convex curve $g(s)$ with a predefined constant curvature $\kappa_1 = 1/R_c$.

The above serves as a basis for the design of the lens-box in-plane tessellation that upon folding can smoothly conform to a predefined curved surface on target. For surfaces with intrinsic curvature, we can attain curvature smoothness along one of the principal directions, and piecewise linear approximation along the other, as our unit is assumed to consist of inextensible developable membranes. We achieve curvature smoothness along one of the principal directions by smoothly connecting locked lens box units along a prescribed curve g, and we program the piecewise approximation of the curve in the second principal direction, by tailoring the waterbomb connector geometry, as explained in the next section.

Given the similarity of the two portions of the lens-unit, only one side, the right, is here examined. The corresponding relations can be analogously derived for the left side. With reference to the right portion of the lens unit in Fig. S14a, we assume a given constant value of curvature $\kappa_1 = 1/R_c$ for the *zx*-plane projection of the folded lens panel M (Fig. S14b). We use non-parametric cartesian coordinates, as $(x - x_c)^2 + (z - z_c)^2 = R_c^2$ with $C(x_c, z_c)$ being center coordinate (focus point) of the constant curvature arc (which remains to be determined), to write the general equation of the curved crease projection onto *xz*-plane as

$$g(s) = \mathcal{G}(\lambda(s)) = \pm\sqrt{R_c^2 - (\lambda(s) - x_c)^2} + z_c. \tag{38}$$

where the curve g is convex for $z_c < 0$ and concave if $z_c > 0$. Upon denoting the angle between the -*x* axis and the line connecting the focus point $C(x_c, z_c)$ to the *xz*-plane projection of the $f(A^-)$ by ψ_0, and the angle between the *xz*-plane projection of the two lines connecting the focus point

$C(x_c, z_c)$ to the $f(A^-)$ and the folded crease point s by ψ, we can write the relations $\cos(\psi + \psi_0) = (x_c - \lambda)/R_c$ and $\psi = s/R_c$ (see Fig. S14b) and obtain $\lambda(s)$ as

$$\lambda(s) = x_c + \sqrt{R_c^2 - {x_c}^2}\sin\left(\frac{s}{R_c}\right) - x_c\cos\left(\frac{s}{R_c}\right), \tag{39}$$

where $x_c = \sqrt{R_c^2 - (h_0 - z_c)^2}$ and the thickness of the folded lens-box h_0 (Fig. S14b) is the third component of the vector $\overrightarrow{f(B^-)f(A^-)}$ (see Fig. S5) and is given by

$$h_0 = b_3 = \sqrt{\left(\frac{v}{2} - \ell_o\right)^2 - \frac{\left(\frac{v^R}{2} - u\sin\mu - \ell_o\right)^2}{\cos^2\mu}}. \tag{40}$$

In addition, we can obtain $u = \frac{1}{2}(v^M - v^R)/\sin\mu$ (see Fig. S4b).

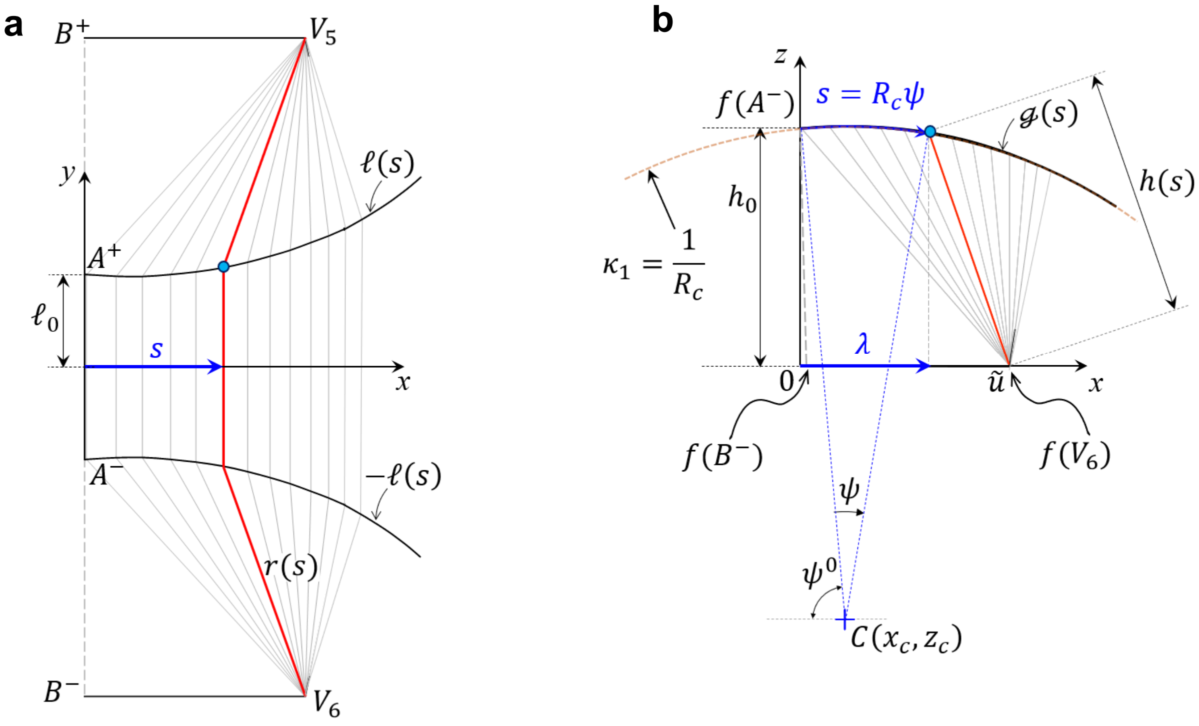

Fig. S14. Right portion of the lens unit. (a) Schematic of lens unit in the unfolded configuration showing rulings. (b) *xz*-plane projection of the folded lens unit shown in (a) with associated geometrical parameters used for the inverse problem.

The distance from the crease point s and cone ruling vertex $f(V_6)$ (or $f(V_5)$) in the orthogonal *xz*-plane projection of the folded lens unit equals $h(s)$ and can be calculated as

$$h(s) = \sqrt{(\lambda(s) - \tilde{u})^2 + \left(g(s)\right)^2}, \tag{41}$$

where $\tilde{u} = u\cos\mu + (\frac{v^R}{2} - u\sin\mu - \ell_o)\tan\mu$ denotes the *x*-coordinate of the folded vertex $f(V_6)$ (and $f(V_5)$), and $g(s)$ and $\lambda(s)$ can be obtained from relations (38) and (39), respectively. By equating Eq. (41) and (5) we obtain the initial unfolded curved crease $\ell(s)$. Then, for any given u, ℓ_0, v, and R_c and the two folding parameters v^R and μ we can find the coordinate z_c and so x_c by imposing the smooth folding boundary condition previously obtained for $\ell'(0)$, i.e., relation (3).

Since the lens unit only covers a small part of the prescribed smoothly curved surface, to cover a larger area, we need to tessellate the lens unit along the curve, a step explained in the next section.

4.1 Smooth Tessellation Condition

To tessellate a collection of lens-units for conformal folding into a prescribed curve g with constant curvature, we need to determine the geometry of the unfolded leg panels and the length of the unfolded lens panel, u_c, of adjacent units, or more precisely to determine the coordinate of V_8 and V_2 as shown in Fig. S15a. For the following analysis and without losing generality, we examine only a portion of the lens unit pattern (the right in Fig. S4a) and assume that the coordinates of the vertices V_5 and V_6, and the crease points A^- and A^+ are known from our previous analysis. We also denote the crease points A^- and A^+ as vertices V_3 and V_4, and include the effect of the panel thickness in our formulation by introducing the parameter w_t as a measure of flat-folded waterbomb connector unit. Since the flat-folded connector units has 8 layers panel overlapping, $w_t = 8t$. However, when we distribute this effect between the right and the left lens units, we should use $w_t = 4t$ for each side units. In continue, we formulate a general case with the panel thickness contribution of w_t, and study the lens unit in its locked state (Fig. S15a and b) with the goal of conforming the tessellation of locked units to an arc of prescribed curvature radius R_c.

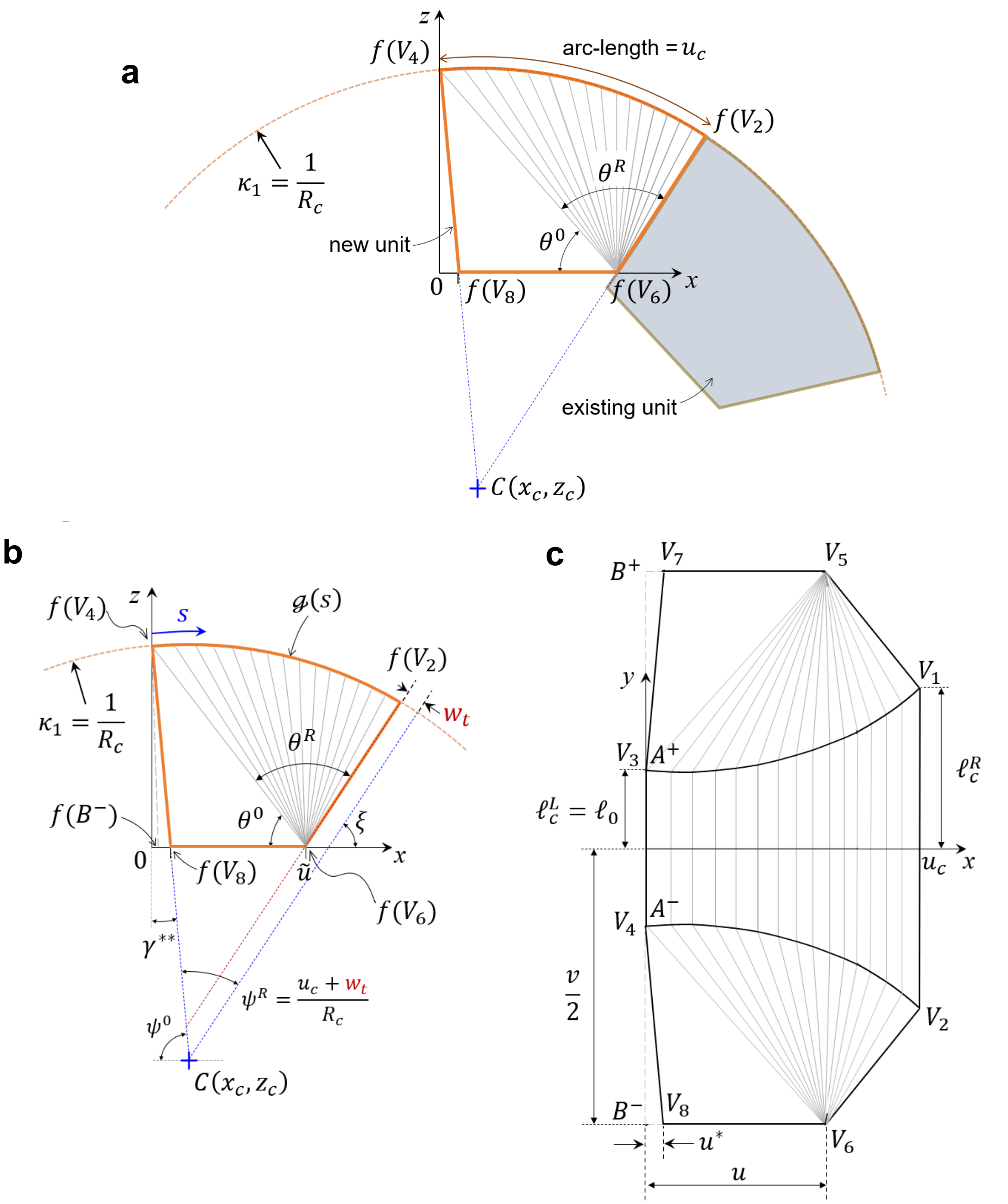

Fig. S15. Representative lens unit in locked (orange) and unfolded state (black) for smooth tessellation conforming to a predefined curve. (a) Schematic of connecting a locked lens unit to the left-side of its adjacent (existing) lens unit. (b) Isolated locked lens unit with geometrical parameters and vertices guaranteeing smooth connection in the lock state. (c) Lens unit (b) in its unfolded state with x-coordinate of the vertices V_7 and V_8.

One way to tackle the above is to consider the *xz*-plane projection of the lens unit as a portion of a slice with angle $\psi = \psi^R = (u_c + w_t)/R_c$ taken from a disc with radius R_c (Fig. S15a). If ξ is the angle that the right side of the *xz*-plane orthogonal projection of the folded leg panel forms with the x-axis (Fig. S15b), and we can obtain

$$\sin\xi = \frac{g(u_c)}{h(u_c)} = \frac{|z_c|}{R_c \mp h(u_c) + w_t \tan(\psi^R - \gamma^{**})}, \tag{42}$$

which upon replacing $\gamma^{**} = \sin^{-1}(x_c/R_c)$ and $\psi^R = (u_c + w_t)/R_c$ can be rearranged as

$$g(u_c)\left(R_c \mp h(u_c) + w_t \tan\left(\frac{u_c + w_t}{R_c} - \sin^{-1}\left(\frac{x_c}{R_c}\right)\right)\right) - |z_c| h(u_c) = 0, \tag{43}$$

whose solution gives the arc-length u_c (Fig. S15a) for folding the curved crease into a smooth curve with given curvature. In Fig. S15a and Fig. S15b, the overlaid orange lines show the vertices, creases and edges that correspond to the geometry of the lens unit obtained through relation (43) for the locked lens unit.

Given the equation above pertains to the right-hand side of the lens unit, i.e., the coordinates of V_1 and V_2, we now derive the relation that gives the coordinate of V_7 and V_8 on the left-hand-side of the *U* and *L* panels in Fig. S15c. Also here, we observe that the folded vertices $f(V_7)$ and $f(V_8)$ must lie on the line connecting the curved crease point $s = 0$ to the focus point of the prescribed arc g, i.e., $C(x_c, z_c)$. We can obtain the horizontal distance u^* between B^- and V_8 on the bottom of *L*, and B^+ and V_7 on the bottom of *U* defining the *x*-coordinate of the vertices on the lower and upper left corners with

$$u^* = \frac{\mathcal{G}'(0)h_o - (\frac{v^M}{2} - \ell_o)\tan\mu}{\cos\mu}. \tag{44}$$

Relations (43) and (44) serve to obtain only the necessary conditions for the tessellation of the lens units into a smoothly curved surface of constant curvature. These, however, do not describe the connection between the waterbomb connector unit and the lens unit necessary to generate the tessellation for a prescribed surface mapping. The following section tackles this complementary problem.

5 Tessellation Generation for Target Surface Mapping

So far, we have shown the necessary conditions and constraints for the smooth folding of the lens unit, the flat foldability of the waterbomb connector units, the attainment of a smooth connection

of the lens-box units upon tessellation, and the general rigid-ruling foldability of the entire crease pattern. Our relations for the individual lens unit and waterbomb connector suggests that the design space of each unit is very rich; an infinite number of lens units exist that conform into a given surface with constant curvature, and an infinite number of connector units exist that can flat fold for a given geometry of the lens unit. It is therefore plausible that we can find at least one tessellation that exactly matches the first (global) principal curvature κ_I of the target surface along the lens panel, i.e., arc-length s, and well-approximates its second principal (global) curvature κ_{II}. In the following, we first explicitly formulate the construction of generalized cylindrical surfaces with $\kappa_{II} = 0$, and then, propose a numerical optimization algorithm to search for locked lens-box embeddings for a target surface that is intrinsically (non-zero Gaussian curvature) curved. In our analysis, we recall that the radius of the first principal global curvature is denoted with R_{GI} and that of the second one with R_{GII}; we reserve the notation R_c to refer to the local radius of the curvature of the lens panel *M*.

5.1 Generalized Cylindrical Surfaces

A cylindrical surface can be generated by extruding a 2D curve along the axis perpendicular to the plane of the curve. For a general cylindrical surface, the lens unit folds with $\mu = 0$ which in turn leads to $\tilde{u} = u$, and $x_c = 0$, conditions that simplify our relations to $\lambda(s) = R_c \sin\left(\frac{s}{R_c}\right)$, $g(s) = \pm R_c\left(\cos\left(\frac{s}{R_c}\right) - 1\right) + h_0$ and $h_0 = \sqrt{\left(\frac{v}{2} - \ell_o\right)^2 - \left(\frac{v^R}{2} - \ell_o\right)^2}$. Moreover, the waterbomb connector unit flat folds into a geometry with angle $\varphi = 0$ (see Fig. S6c and the relation (20)). While our lens-box tessellation is capable of approximating smooth cylindrical surfaces of almost all combinations of curvature values (both concave and convex) as we discuss next, we can show that our locked pattern can also tessellate into a non-smooth surface with periodic smooth unit cells or dents suggesting the possibility of creating foldable gear sets or other complex mechanisms.

5.1.1 Constant-Curvature Tessellations

To find tessellation patterns which upon locking conform to a cylindrical surface ($R_{GI} = R_c$) with smooth curvature of constant-value, we assume the unfolded lens box unit and its folded

embedding symmetric with respect to the y-axis, i.e., the shorter diagonal of the lens panel M (Fig. S8 top-right). This assumption implies that the geometry of the right and the left waterbomb connectors are identical with $\ell_c^R = \ell_c^L$ and $v^R = v^L$. For any given set of parameters v, u and ℓ_0 defining the initial 2D crease pattern and prescribed lock parameters v^R and R_c, we determine the geometric parameter u_c by solving the constraint Eq. (43), which guarantees smooth connection of the adjacent lens units. Then, using the initial parameters of the lens unit with an initial guess for the angle $\alpha \geq 0$ in the waterbomb connector pattern (Fig. S6b), we first calculate the length a by solving Eq. (20). If a satisfies the constraint Eqs. (21-25), this value a is a solution; else, we incrementally increase the angle α until we might find an admissible solution. If the search is not successful, we can vary one of the other initial geometric parameters v, u and ℓ_0, or folded parameter v^R which also influences the outcome of the trial-and-error process. Fig. S13b illustrates one example for a pattern that can lock into a surface with convex constant-curvature. From constant curvature we now move to the analysis of tessellation conforming to a surface with variable curvature.

5.1.2 Smooth Variable-Curvature Tessellations

To address the problem of conforming to a smooth cylindrical surface with variable curvature, we first approximate the directrix of the curved surface Γ using i constant-curvature partition $\Gamma_1, \Gamma_2, \dots, \Gamma_i$ with radius of $R_{c1}, R_{c2}, \dots, R_{ci}$. Then, we design the first locked unit such that its isometric embedding matches the curvature radius of the first partition R_{c1} using the approach explained above for constant-curvature tessellations. This solution yields the initial geometric parameters $v, v^R = v^L, u, u_c$ and $\ell_c^R = \ell_c^L$ for the lens unit, which can in turn be used to obtain the crease geometry for the waterbomb connector unit, where $e_x = u_c - u$ and $e_y = \frac{v}{2} - \ell_c^R$ (see Fig. S6b). For any other partition curve Γ_j $(j > 1)$, an additional geometrical constraint must be imposed since the compatibility between two connected lens-box units requires that the parameters v, v^R, ℓ_c^R and e_x^R do not vary. This leads to a slightly modified version of the smooth tessellation constraint Eq. (43). In addition to this, we need to formulate another constraint to hold the parameters v, v^R, ℓ_c^R and e_x^R constant in our lens-box units as explained below.

To formulate our new constraint, we first introduce the additional subscript j to denote any attributes related to the jth partition (with constant curvature radius of R_{cj}) for $j \neq 1$. For example,

we use $h_3(s)$ to refer to the function $h(s)$ for the partition Γ_3 but simply use R_c to refer to the curvature radius of the first partition Γ_1. With this notation, the height of the trapezoid $h_j(s)$ is given by $h_j(s) = \sqrt{(u_j - s)^2 + (v - v^R)(\frac{v+v^R}{4} - \ell_j(s))}$. At the boundary crease point $s = u_{cj}$, i.e., the end point of the curved crease on vertex V_1, the curved crease function $\ell_j(u_{cj}) = \ell_c^R$, and $u_j - u_{cj} = e_x$, as the connector units for the right and left lens units are identical. Since the geometry of the first unit is determined in the initial step, $h_j(u_{cj})$ can be expressed in terms of the geometrical parameters of the first unit as

$$h_j(u_{cj}) = \sqrt{(e_x)^2 + (v - v^R)(\frac{v + v^R}{4} - \ell_c^R)}. \tag{45}$$

The right-hand-side of relation (45) is constant as it can be determined for given geometric values of the first unit. As a result, relation (45) serves as the additional constraint for the subset of lens-box units that can match the partition curves Γ_j $(j \neq 1)$.

We now replace $s = u_{cj}$ and $u_j = e_x + u_{cj}$ in relation (41), and introduce the constant $C = h_j(u_{cj})$ obtained from (45), to express the thickness h_{0j} of the folded lens-box as a function of u_{cj} and R_{cj} as

$$\begin{aligned} h_{0j}(u_{cj}, R_{cj}) = {} & \mp R_{cj}\left(\cos\left(\frac{u_{cj}}{R_{cj}}\right) - 1\right) \\ & \pm \sqrt{R_{cj}\sin\left(\frac{u_{cj}}{R_{cj}}\right)\left(2(e_x + u_{cj}) - R_{cj}\sin\left(\frac{u_{cj}}{R_{cj}}\right)\right) - (e_x + u_{cj})^2 + C^2}. \end{aligned} \tag{46}$$

Similarly, by replacing (45) in the smooth tessellation constraint Eq. (43) and rearranging, we can obtain another relation for the folded lens-box thickness h_{0j} as a function of u_{cj} and R_{cj}, in the form of

$$h_{0j}(u_{cj}, R_{cj}) = \frac{R_{cj}C \mp R_{cj}\left(\cos\left(\frac{u_{cj}}{R_{cj}}\right) - 1\right)\left(R_{cj} + w_t \tan\left(\frac{u_c + w_t}{R_c}\right) \mp C\right)}{R_{cj} + w_t \tan\left(\frac{u_c + w_t}{R_c}\right)}. \tag{47}$$

Since the thickness of the folded lens-box is $h_{0j} = \sqrt{\left(\frac{v}{2} - \ell_{oj}\right)^2 - \left(\frac{v^R}{2} - \ell_{oj}\right)^2}$, we can also obtain ℓ_{oj} as a function of the two parameters of u_{cj} and R_{cj} as

$$\ell_{oj}(u_{cj}, R_{cj}) = \frac{h_{0j}(u_{cj}, R_{cj})^2}{v^R - v} + \frac{1}{4}(v^R + v). \tag{48}$$

To obtain the solution space of admissible u_{cj}, the right-hand-side of the two relations (46) and (47) can be equated and solved for a given R_{cj}. Alternatively, one may seek the intersection of the two functions $\ell_{oj}(u_{cj}, R_{cj})$ obtained by substituting the two relations (46) and (47) in (48). To visualize the solution space of the all lens-box units compatible with a predetermined connector unit (here obtained for the first lens-box unit using the parameters (in mm) $u = 15, v = 60, \ell_0 = 10$, $v^R = v/2$, $e_x = 5.16$, $R_c = 70$, $w_t = 0$ and $\delta = 0°$), we plot the function (48) using the two input functions obtained for $h_{0j}(u_{cj}, R_{cj})$ in (47) (brown solid line) and (46) (blue dash line) for a given set of R_{cj} in Fig. S16a and b. The admissible solution for u_{cj}, which is the intersection of these two plots, is depicted by red circles. Inadmissible regions highlighted in both plots pertain to the cases when $u < 0$ (shown by blue shades). Interestingly, we realized that the locus of all solution points (red circles) approximately lays over a line with equation $\ell_{0j} = mu_{cj} + b$ where the slope m and the *y*-axis interception *b* (here interception with ℓ_{0j}) can be obtained as follows.

To determine the linear equation $\ell_{0j} = mu_{cj} + b$ we need to know at least the coordinates of the two points on this line. One of these points can be the interception of this line with the *y*-axis (here ℓ_{0j}) which can be easily determined by setting $u_{cj} \to 0$ and $\ell_{0j} \to \ell_c^R$. Next point can be obtained by determining an intersection of the two Eqs. (46) and (47) that occurs on the *x*-axis (here u_{cj} axis). At such point $h_{0j}(\ell_{0j} = 0) = \sqrt{\left(\frac{v}{2}\right)^2 - \left(\frac{v^R}{2}\right)^2}$ becomes a constant. Upon replacing this in (46) we can obtain u_{cj} as a function of R_{cj}, i.e., $u_{cj}(R_{cj})$, which substituted in (47) yields to the coordinates of the second point. If the effect of the panel thickness is neglected, $w_t = 0$, the *x*-axis intercept can be obtained as

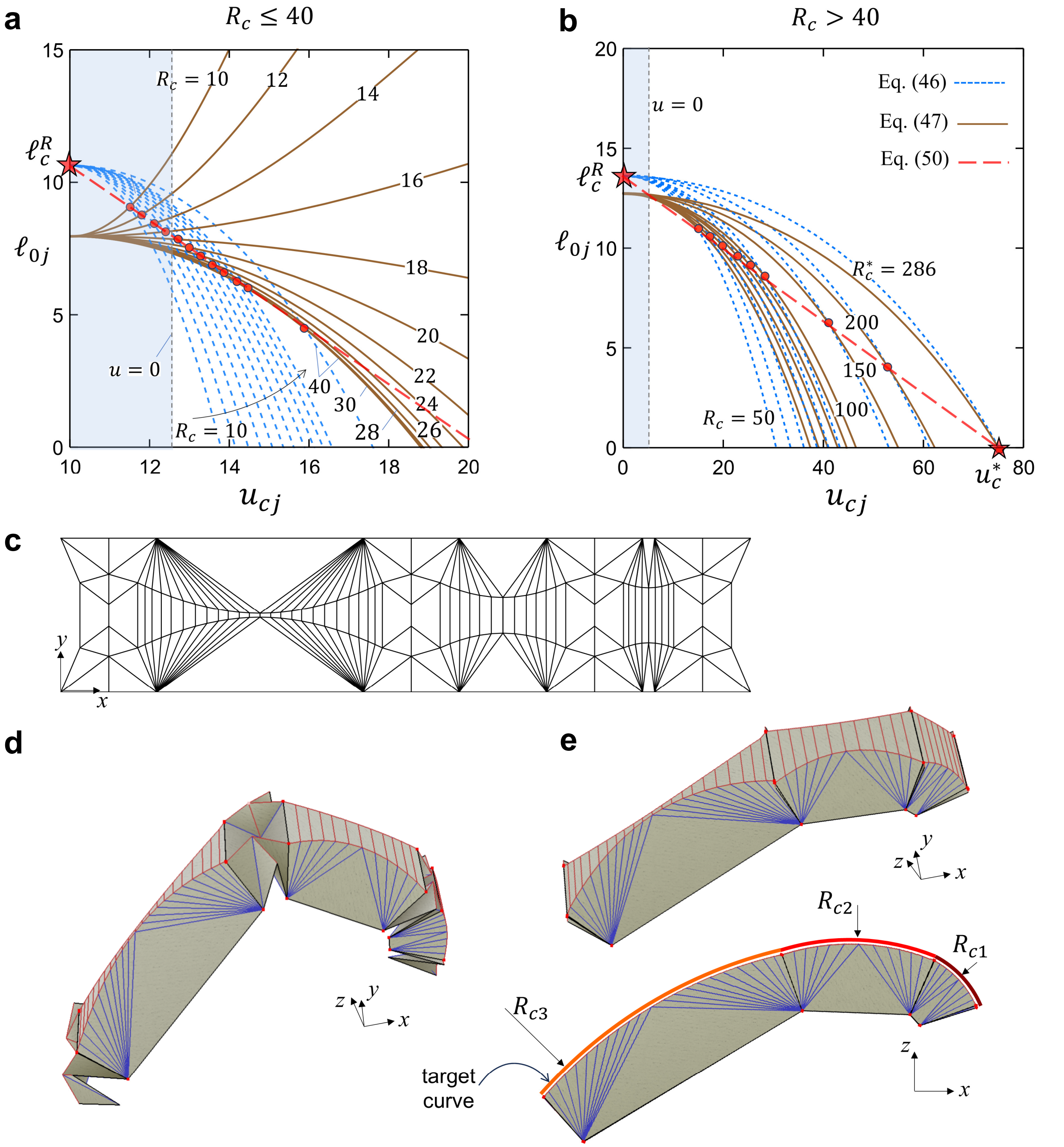

Fig. S16. Folding into a smooth surface with variable-curvature using a unique connector unit. Parametric plot of the function $\ell_{oj}(u_{cj}, R_{cj})$ obtained by substituting the two relations (46) and (47) in (48) when $R_{c1} \leq 40$ (a), and when $R_{c1} > 40$ (b) for a predetermined connector unit designed for the initial lens-box unit with parameters $u = 15, v = 60, \ell_0 = 10$, $v^R = v/2$, $e_x = 5.16$, $R_c = 70$, $w_t = 0$ and $\delta = 0°$. (c) A 1D tessellation pattern that upon locking conforms into a variable-curvature curve comprising three partitions with curvature radius of 40, 60 and 140. Design variables of different partitions in (c) are: $R_{c1} = 40$: $u = 12, \delta = 27°$; $R_{c2} = 60$: $u = 14, \delta = 26°$; $R_{c3} = 140$: $u = 22.6, \delta = 27.2°$. The rigid folding simulation of the pattern (c) in its partially folded state (d) and in its lock state (e). Simulations (c) and (d) are performed using *Freeform* software [19]. All dimensions are in mm units.

$$u_{cj} = u_c^* = R_{cj} \cos^{-1}\left(\frac{R_{cj} \mp \sqrt{\left(\frac{v}{2}\right)^2 - \left(\frac{v^R}{2}\right)^2}}{R_{cj} \mp C}\right). \tag{49}$$

By replacing (49) in relation (46) and solving for R_{cj} we can obtain $R_{cj} = R_c^*$, a point at which the two curves intersect on the x-axis. Thus, the equation of the loci of all solution points for u_{cj} becomes

$$\ell_{0j}(u_{cj}) = -\frac{\ell_c^R}{R_c^* \cos^{-1}\left(\frac{R_c^* \mp \sqrt{\left(\frac{v}{2}\right)^2 - \left(\frac{v^R}{2}\right)^2}}{R_c^* \mp C}\right)} u_{cj} + \ell_c^R. \tag{50}$$

Note that for $w_t \neq 0$ obtaining a similar relation for u_c^* can become challenging. An alternative way to find Eq. (50) is by obtaining two design points (red dots), each given by the intersection of the two lines expressed by Eqs. (46) and (47)) in Fig. S16a and b.

While in this section we show how to connect the two lens-box units of dissimilar curvatures, the limitation of using a unique connector geometry over the entire tessellation pattern can pose a challenge in connecting two lens-box units with different curvature signs: The geometry of the connector units in the lens-box patterns that conform into convex surfaces are fundamentally different than those that conform to concave surfaces: In the convex case, we always have $\overline{V_5V_{15}} > \overline{V_1V_{11}}$ while in the concave case, $\overline{V_5V_{15}} < \overline{V_1V_{11}}$ (see Fig. S6); compare, for example, the two illustrated patterns in Fig. S13b and a. To address this shortfall, we construct a single lens-box unit cell that, by locking its right and left lens panels, conforms into surfaces with curvatures of opposite signs while maintaining a smooth connection. In the following section, we show that such design allows us to vary the connector unit geometry and thus enables to connect a concave lens-boxes to a convex one smoothly.

5.1.3 A Single Unit for Smooth Curvature Transition

To construct a single lens-box unit which upon locking conforms into a surface – comprising two curvature of opposite signs, we examine a lens panel with two dissimilar functions ℓ_c^R and ℓ_c^L and $\ell_c^R(s=0) = \ell_c^L(s=0) = \ell_0$ respectively for the right and left curved crease. Although with this assumption we forgo the symmetry assumption with respect to the *y*-axis, we maintain the symmetry along the other axis for both the unfolded and folded unit along the *x*-axis. This assumption has an important implication. The tangent to the curved crease at $s = 0$ (the triangular region apices A^+ in U and A^- in L) always stays parallel to the *x*-axis, hence satisfying the condition of smooth folding. In other words, we can show that $\frac{dg(s)}{ds} = \frac{d\ell(s)}{ds} = 0$ as one may realize from the relation of the tangent vector at A^- in Eq. (1). Additionally, the orthogonal projection of the imaginary line $f(A^-B^-)$ onto *xz*-plane always stays on the *z*-axis. Therefore, the right and the left side of opposite curvature lens-box units can join while preserving the overall rigid-ruling foldability and smoothness as long as the three parameters "v, $v^R = v^L$ and ℓ_0" are identical for both sides: These parameters govern the geometry of the connection region in the *yz*-plane.

With the understanding above, we can now create a single lens unit pattern which by locking its right-side conforms into a curve with curvature radius R_c^R, and its left-side into its counterpart with R_c^L. Given the three parameters "v, $v^R = v^L$ and ℓ_0" remain unchanged in the geometrical transition, the parameters u^R, u_c^R and ℓ_c^R can be used to design the right-hand side of the pattern independent from the parameters u^L, u_c^L and ℓ_c^L used for its left-hand side. Unlike the previous case, here the geometry of the right and left waterbomb connectors may be dissimilar. An advantage of the strategy pursued here is to enable the design of units which can lock into an inflection point of a curved surface, where the curvature transitions from concave to convex occurs. Furthermore, this approach enables to connect half-units (half-units are the half of the double-symmetric units divided by the *zy*-plane) using the connector units. In this case, the dimensions of the waterbomb geometry can be obtained through v, $v^R = v^L$, $\ell_c^R = \ell_c^L = \ell_0$ with $e_x = 0$ and $e_y = v/2 - \ell_0$ (see Fig. S6b and c). Fig. S17 shows two distinct examples where multiple lens-box units with curvature of opposite signs approximate a curved surface with varying curvature. In this example, the target curve is shown with a thick solid line slightly above the *xz*-plane projection of the locked pattern. Each shade and color denote a region with given curvature value and sign.

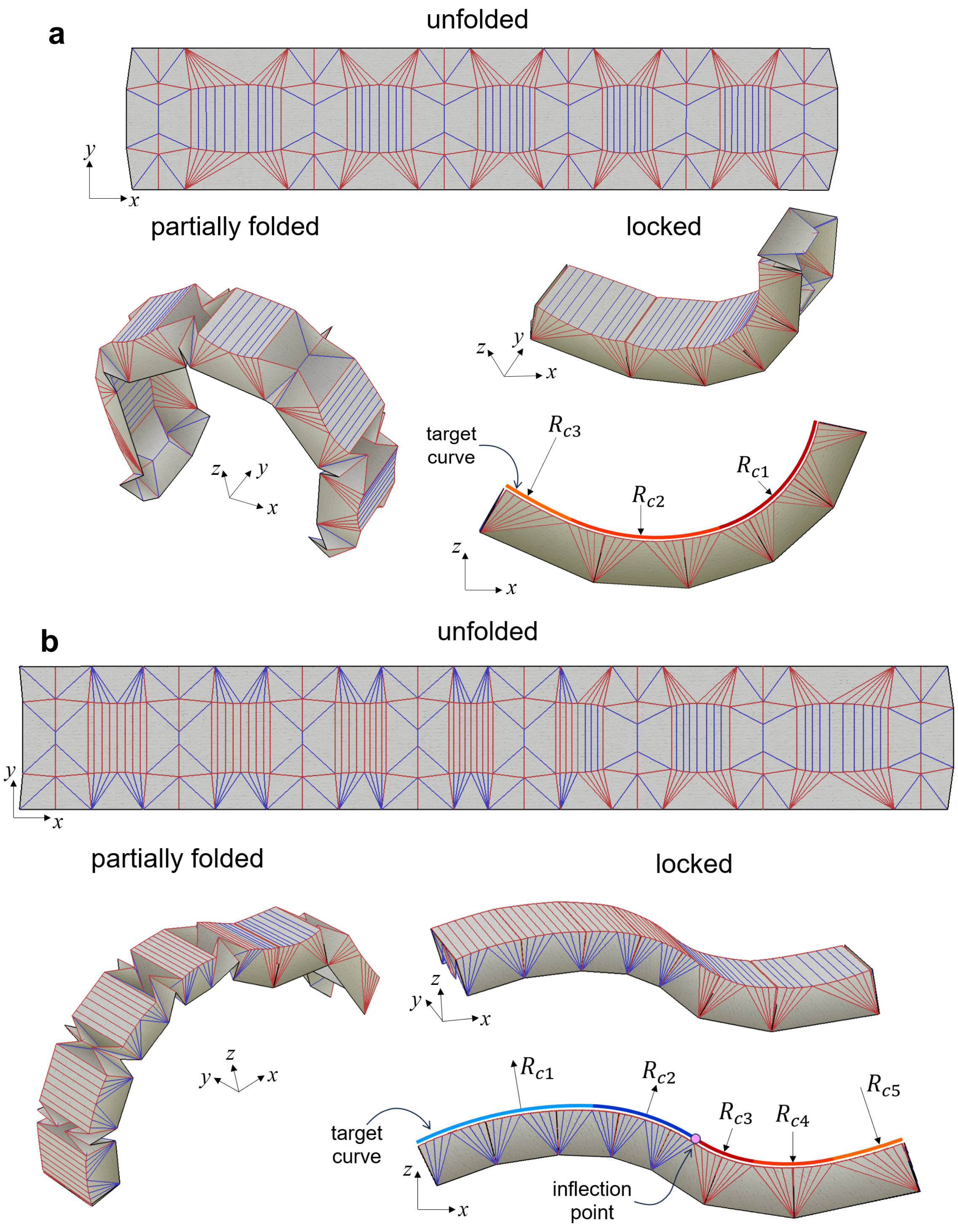

Fig. S17. Lens-box pattern showing folding into smooth surfaces with variable-curvature. Two examples of crease pattern inversely designed to conform upon locking to a portion of a smooth concave surface with variable-curvature (a) and a surface with a combination of convex variable-curvature and concave variable-curvature (b) shown in the unfolded, partially folded and lock state. For all units values prescribed are $v = 50, \ell_0 = 12$ and $v^R = v^L = v/2$. Design variables of the partitions in example (a) are: $R_{c1} = 40$: $u = 12, \delta = 27°$; $R_{c2} = 60$: $u = 14, \delta = 26°$; $R_{c3} = 140$: $u = 22.6, \delta = 27.2°$. Design parameters of the partitions in (b) are: $R_{c1} = 110$: $u = 9, \delta = 0°$; $R_{c2} = 60$: $u = 6, \delta = 0°$; $R_{c3} = 40$: $u = 12, \delta = 27°$; $R_{c4} = 60$: $u = 14, \delta = 26°$; $R_{c5} = 140$: $u = 22.6, \delta = 27.2°$. All dimensions are in mm units. Simulations are performed using *Freeform* software [19].

The above has demonstrated the realizations of patterns folding into smoothly curved cylindrical surfaces. A complementary problem is to forego smoothness and intentionally design periodic alternation of concave or convex units curved with potential applications for deployable gear-like mechanisms realized from thin flat sheets as examined next.

5.1.4 Not-C^1 Periodic Tessellations of Smooth Unit-Cells

To achieve a tessellation which upon folding conforms into a not-C^1 surface with global curvature radius of R_{GI} with constant-curvature smooth cells of convex or concave type, we need to modify the constraint Eq. (43). Consider the locked lens-box unit sketched in Fig. S18a. The angle between the xz-plane projection of the right-most ruling line and the positive x-axis, i.e., ξ illustrated in Fig. S18b, can be obtained by the relation $\sin\xi = \frac{g(u_c)}{h(u_c)}$. If this unit is a slice of a general surface with curvature radius of R_{GI}, we can also obtain ξ as a function of R_{GI} using the relation $\cos\xi = \mp u/(h(u_c) \mp R_{GI} + w_t \tan\xi)$ which upon replacing ξ from the relation $\sin\xi = \frac{g(u_c)}{h(u_c)}$ yields the constraint equation

$$\left(h(u_c) \mp R_{GI} + w_t \frac{\frac{g(u_c)}{h(u_c)}}{\sqrt{1-\left(\frac{g(u_c)}{h(u_c)}\right)^2}} \right) \sqrt{1-\left(\frac{g(u_c)}{h(u_c)}\right)^2} \pm u = 0. \tag{51}$$

The solutions of Eq. (51) give the arc-length u_c (Fig S15a) for folding the curved crease into a curve of given local curvature radius R_c and global curvature radius R_{GI}. Also here, the upper sign in ($\mp$ or $\pm$) refers to the convex surface with global positive curvature, while the lower sign refers to the concave surface with global negative curvature. When $R_{GI} \to \infty$ (for generally flat surfaces with zero curvature) the equation above reduces to $h(u_c) - g(u_c) = 0$.

Figure S19 illustrates three examples of different types of not-C^1 periodic surfaces by varying the global curvature radius of the surface and its local curvature radius. Here we show a combination of global not-C^1 convex surface with concave or convex unit-cells, and global not-C^1 concave surface with convex unit-cells; we avoid showing the case with global not-C^1 concave surfaces with local concave units for the sake of brevity.

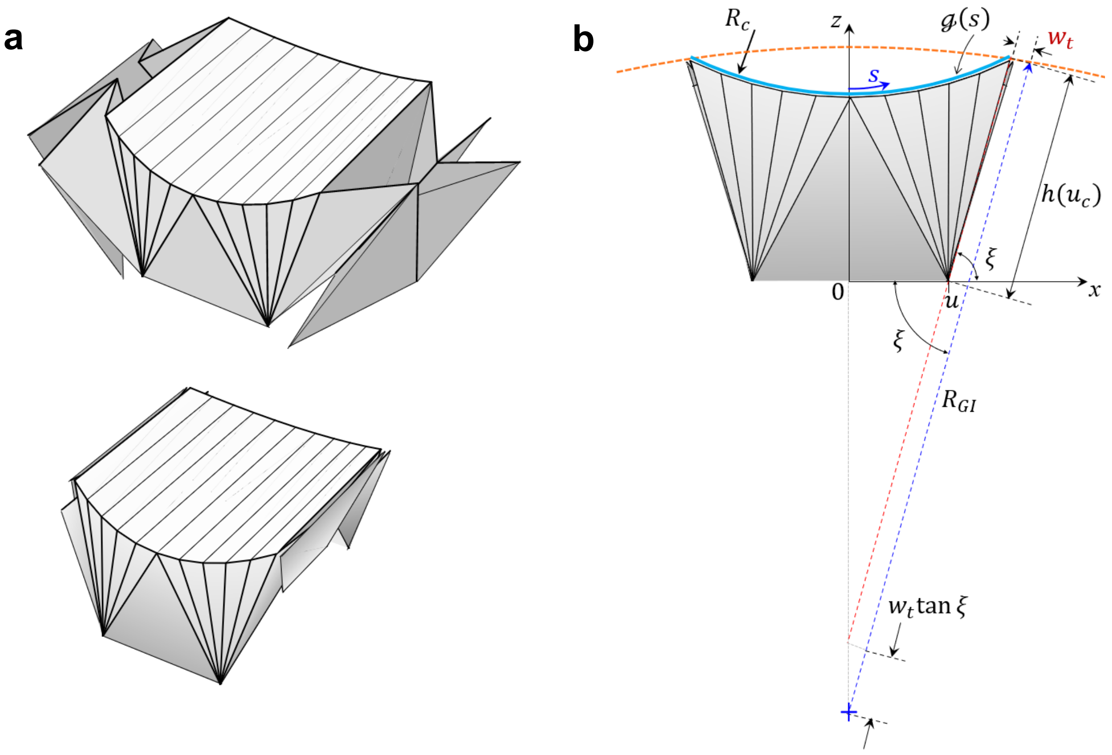

Fig. S18. Geometry of the lens-box unit for not-C^1 tessellation conforming to a predefined curve. (a) A lens-box unit with concave curvature in partially folded (top) and locked (bottom) states. (b) *xz*-plane projection of the locked unit representing the geometrical details for obtaining the not-C^1 periodic tessellation constraints. This example represents the global convex curve.

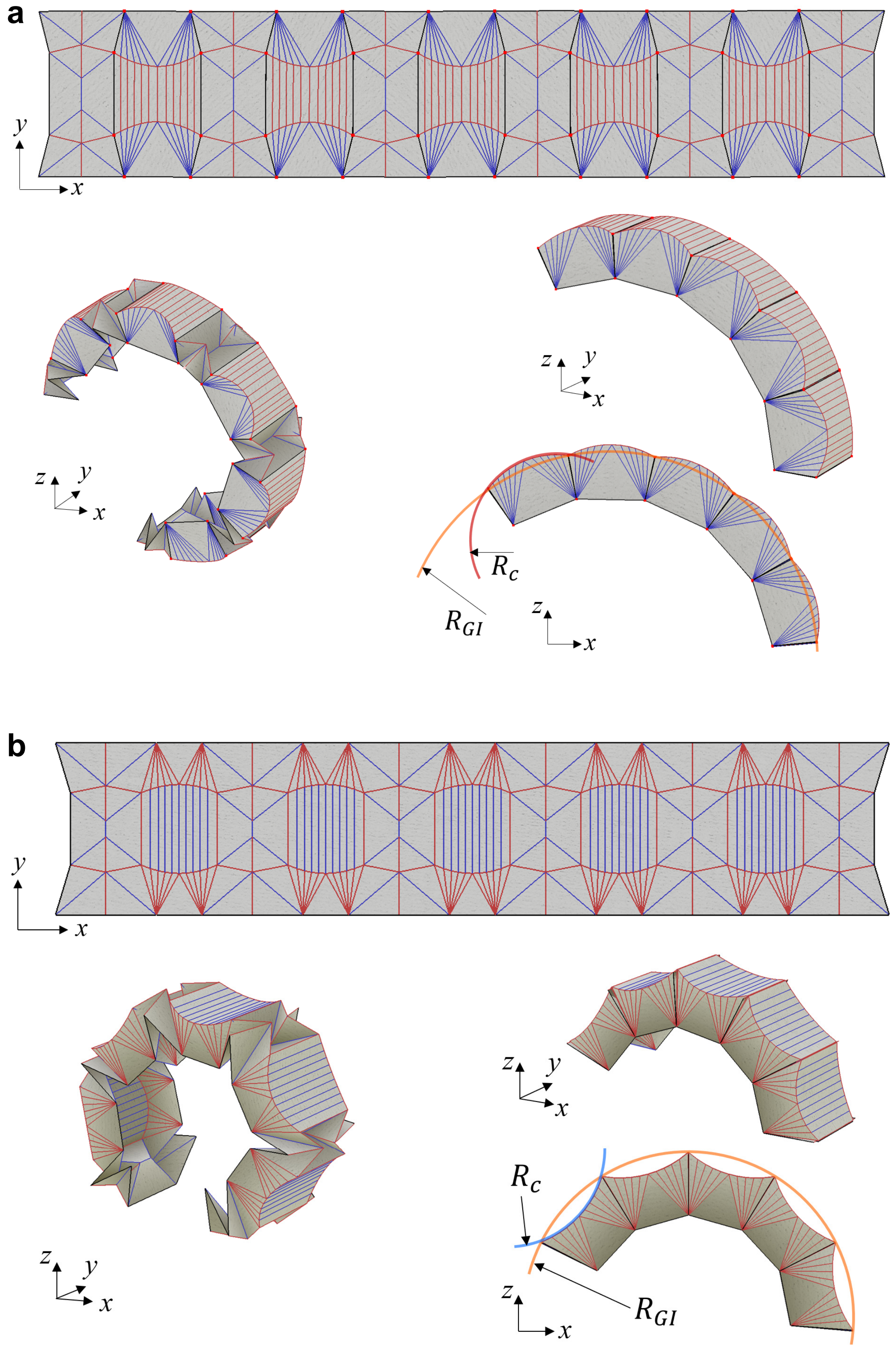

(This figure continues on the next page)

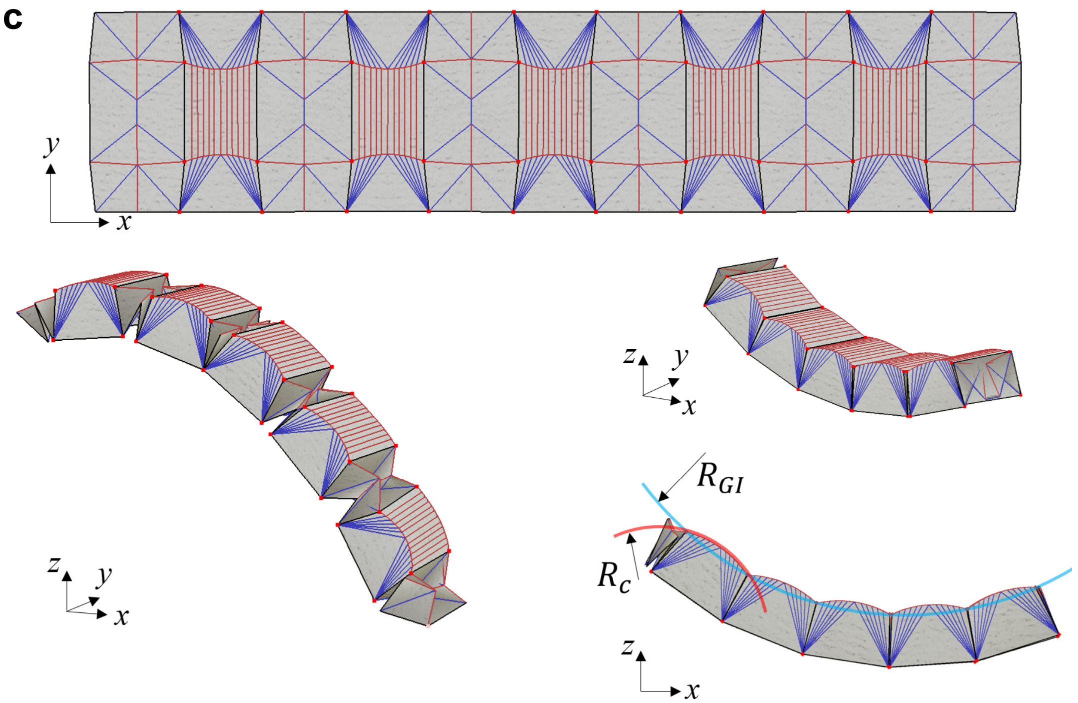

Fig. S19. The lens-box pattern for folding into not-C^1 curved surfaces. Three examples of a designed crease pattern that upon locking conform to a part of a not-C^1 surface with periodic features. (a) A general convex surface with local convex unit cells. (b) A general convex surface with concave unit cells. (c) A general concave surface with convex unit cells. Geometric parameters $v = 60$ mm and folding parameter $v^R = v^L = v/2$ is used for all these designs, and the rest of the parameters (in mm) are: (a) $\ell_0 = 10, u = 12, \delta = 0°$, $R_c = 30$ and $R_{GI} = 73$. (b) $\ell_0 = 15.5, u = 8, \delta = 2°$, $R_c = -30$ and $R_{GI} = 48$. (c) $\ell_0 = 12.8$, $u = 12.5, \delta = 10°$, $R_c = 30$ and $R_{GI} = -90$. The negative sign for the radius of curvature denotes its concavity as opposed to the case of a convex surface where it is shown with positive sign. Results obtained via rigid folding simulation using *Freeform* software [19].

The analysis above reveals the richness of the design space for generating cylindrical surfaces using lens-box pattern. In the following section, we study the tessellation embeddings for the general case of double-curvature surfaces.

5.2 Double-Curvature Surfaces

For surfaces with intrinsic curvature (negative or positive Gaussian curvature), we develop an explicit construction method and adopt an additive tessellation approach. Here, we focus only on target surfaces with axially symmetric geometries. While using our lens-box, folding into more complex and non-symmetric curved surfaces is achievable, it is beyond the scope of this work and the subject of follow-up research. In our additive tessellation approach, resembling a layer-by-layer 3D printing process, we first construct the first row (layer) of the structure that matches the

two global principal curvatures κ_I and κ_{II} but the height of the folded construct, i.e., u, is not pre-defined and is obtained during the optimization process. Upon obtaining the folded lens-box for the first row of structure, we compute the design parameters for the second row (layer) such that its fully lock configuration can rigidly connect to that of the first row (connection along the curvature κ_I) without panel bending or leaving any gap at the interface of the connecting facets. This condition necessitates that the two exterior side-edges of the left flat-folded connector of the bottom lens-box unit (see for example, $\hat{f}(V_{17})\hat{f}(V_5)$ and $\hat{f}(V_{18})\hat{f}(V_6)$ edges in Fig. S6c) lie on the exterior side-edges of the right flat-folded connector of the top lens-box unit, although their connecting edge length might be dissimilar. Additionally, this connection must preserve the C^1 smoothness along the principal curvature κ_I.

As a result of the above-mentioned tessellation approach, our unfolded tessellation pattern is connected along the second principal curvature κ_{II} while remains disjoined along κ_I (see for example Figs. S7 and S13*A*). Constructed layers can then be connected by gluing their interface (i.e., trapezoidal panel $V_9V_{11}V_{12}V_{10}$ shown in Fig. S6b) at their lock configuration. The advantage of this strategy is two-folded. First, it streamlines the folding process as our primary experiments reveal that folding a bidirectionally tessellated pattern might be a challenging task, and sometimes impractical. Second, it significantly simplifies the calculation of the unit cell parameters. Note that assuming the connection of the locked units along κ_I in the design of an unfolded tessellation pattern may have two contrasting implications: either (*i*) the waterbomb connector units must be the same for both rows, or (*ii*) the waterbomb connector units are dissimilar while their flat-folded configurations have similar φ (see this parameter in Fig. S6c). The former reduces the number of free variables hence significantly shrinking our design space, and the latter requires a new pattern design. Below, we explain the process using an example.

Let Ω be the given target surface which is created by revolving the planar curve Γ around *x*-axis as shown in Fig. S20a. Then, discretize the surface Ω along the κ_{II} direction, i.e., in the azimuthal direction assuming a spherical coordinate, into *n* units. In the azimuthal direction, each folded lens-box unit must occupy only a portion of the curved surface limited between the two intersecting planes perpendicular to the *zy*-plane and including the *x*-axis with an acute angle $\eta = 2\pi/n$ (see Fig. S20b). We begin our construction from the first-row units by formulating its geometry in the lock state. First, we fit a constant-curvature arc onto the bottom-end portion of the planar curve Γ, which gives the coordinate of the arc center, as shown in Fig. S20 by C1 for the

unit-1. Next, we can solve an optimization problem for a given set of initial geometric parameters with an initial guess for the locking parameter $v^{M\star} = v_0^{M\star}$ (see Fig. S5a) to obtain

$$v_0^{L\star} = v_0^{M\star} - 2u^* \sin\mu. \tag{52}$$

where u^* is given by relation (44) (see Fig. S15c).

Eq. (52) gives the initial lock parameter $v_0^{L\star}$ for a unit cell matching the curvature of the planar curve Γ in the meridional direction, i.e., the first principal curvature κ_I, while it does not guarantee that the tessellation in the azimuthal direction fits the second principal curvature κ_{II} for a given surface. To match the curvature of the tessellated pattern in the azimuthal direction we develop relations based on the geometry of the general 3D surface Ω.

Let us consider again the orthogonal projection of the two locked (inversely designed) units onto the *XZ*-plane in Fig. S20a and the global cartesian coordinate *XYZ*. Let us denote the distance between the focus point of the folded curved crease arc in the first partition, i.e., C1, and the rotation axis of the surface Ω with Z_{c1}. Let us also introduce the two parameters ω and ω_0 to measure the angle between the orthogonal projection of the left flat-folded connector onto *XZ*-plane (shown using the letter L on the locked unit-1) and the *X*-axis, and that of the right flat-folded connector (shown using the letter R on the locked unit-1) and the *X*-axis, respectively. Then, on the *XZ*-plane projection of the lens-box unit (Fig. S20a), for the given value of ω_0, the radial distance between the right-bottom edge of the folded leg panel, i.e., $f(V_6)$ and $f(V_5)$ (see these vertices in Fig. S15c), can be obtained as

$$r_1 = \mathrm{sign}(\kappa_I \kappa_{II})(R_c \mp h(u_c)) \sin\omega_0 + Z_{c1}, \tag{53}$$

where $\mathrm{sign}(\kappa_I \kappa_{II})$ is the sign of the Gaussian curvature $(\kappa_I \kappa_{II})$, and

$$\omega = \omega_0 + \frac{u_c}{R_c}. \tag{54}$$

The upper (lower) sign in $\mp$ corresponds to units with $\kappa_I > 0$ ($\kappa_I < 0$). We also obtain the length of the orthogonal projection of the bottom of the folded leg panel onto the *XZ*-plane as

$$U_1 = \tilde{u} - \mathcal{G}'(0) h_0, \tag{55}$$

which allows us to determine the radial distance between the left-bottom edge of the folded leg panel, i.e., $f(V_7)$ and $f(V_8)$ (see these vertices in Fig. S15c), as

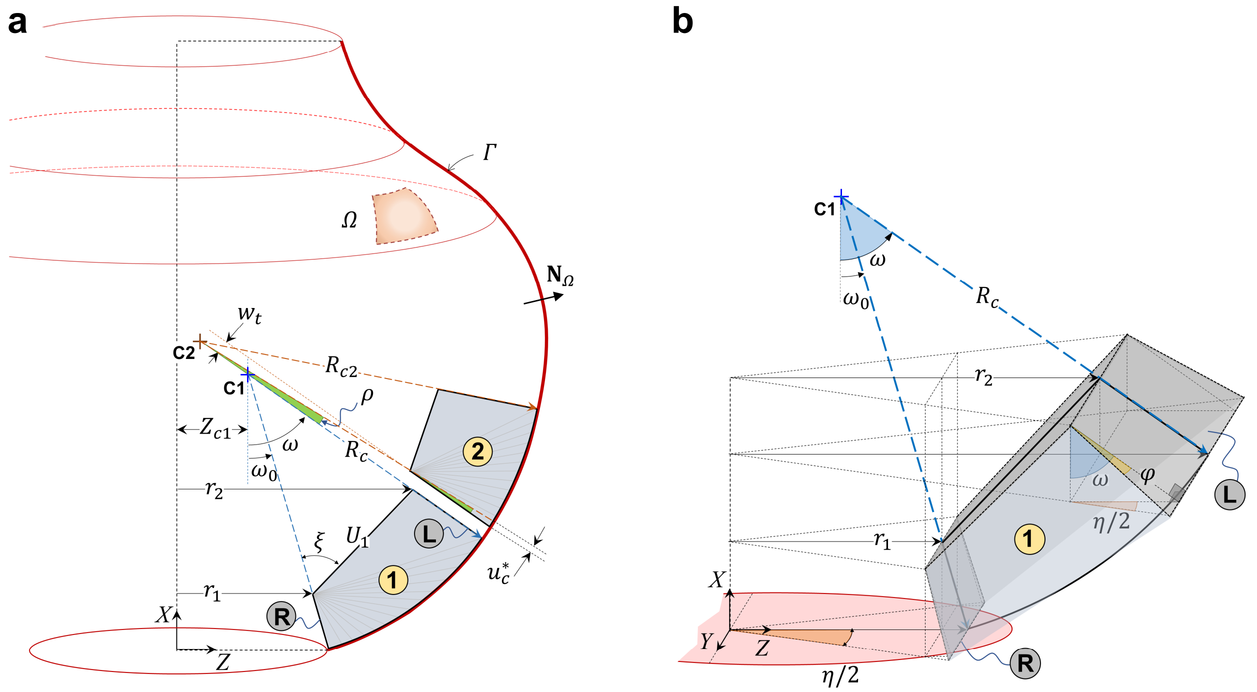

Fig. S20. Geometrical parameters for a double-curvature geometry. (a) Geometric construction and parameters of two representative lens units matching the prescribed double-curvature surface *Ω* created by revolving the planar curve *Γ* around the *X*-axis. The gap between two units in the orthogonal views highlights the role of panel thickness in the connection. (b) Magnified picture of lens-box unit-1 illustrating the geometrical parameters of our formulation in 3D.

$$r_2 = r_1 + \mathrm{sign}(\kappa_I \kappa_{II}) U_1 \sin(\xi \mp \omega_0), \tag{56}$$

where $\xi = \tan^{-1}\left(\frac{z_c}{\tilde{u}-x_c}\right)$ for convex units and $\xi = \pi - \tan^{-1}\left(\frac{z_c}{\tilde{u}-x_c}\right)$ for concave units (see Fig. S15). Using the above relations, we find

$$v^{R\star} = 2r_1 \tan(\eta/2) \tag{57}$$

and

$$v^{L\star} = \frac{r_2}{r_1} v^{R\star} \tag{58}$$

as the predicted lock parameters for the given set of the initial geometrical parameters. The geometric parameter φ for the right and left connector units can be calculated as

$$\tan\varphi^R = \mathrm{sign}(\kappa_{II})\sin\omega_0\tan\left(\frac{\eta}{2}\right) \tag{59}$$

and

$$\tan\varphi^L = \mathrm{sign}(\kappa_{II})\sin\omega\tan\left(\frac{\eta}{2}\right). \tag{60}$$

To simplify the geometric construction process for the double-curvature surfaces, we first exclude the panel thickness effect when writing the smoothness condition Eq. (43) by applying $w_t = 0$. We then can include the panel thickness t, by denoting the effective thickness of the waterbomb connector unit as $w_t = 16t$ (for two joined connector units), and obtaining the thickness-influence angle ρ shown in Fig. S20a as $\rho = \sin^{-1}\left(w_t/\left(R_c - \mathrm{sign}(\kappa_I)h(u_c)\right)\right)$, by simply replacing ω_0 with $\omega_0 + \rho$ in relations (53), (54), (56) and (59). In this case, the obtained value of u_c must also be replaced by the updated value $(u_c + \mathrm{sign}(\kappa_I)u_c^*) = u_c + \mathrm{sign}(\kappa_I)h(u_c)\tan\rho$ (see Fig. S20a) to determine the unfolded crease pattern parameters. Note that construction of the other units beyond the first partition can be treated similarly to the first unit.

The above sets the necessary relations as constraints for attaining a compatible locked lens unit for the double-curvature surfaces construction. Next, we explain our optimization approach to determine the parameters for both the lens-unit and the waterbomb connector units of each layer.

6 Numerical Optimization

The geometry of our unfolded patterns, assuming a given set of initial parameters, can be determined using the relations obtained in the previous sections. In this context, we first define a developable lens box pattern with a variable vector $p^i = \{v, v^R, v^L, u_c, x_c, \ell_c^L, a^R, a^L, \delta^R, \delta^L, \mu\}$. Subsequently, to ensure that these patterns lock into the exact position of the given curved surfaces

discretized into constant-curvature partitions, we formulate our problem as a constrained optimization problem

$$\min_{p^i} \Lambda\left(p^i, p_0^i\right), \quad p^i \in \mathbb{R}_{>0}, \quad s.t.$$

$$\begin{cases} \Phi^j\left(p^i\right) = 0 \\ \Psi^k\left(p^i\right) \leq 0 \end{cases}, \text{ and}$$

$$p_a^q \leq p_q^i \leq p_b^q,$$

where Φ^j and Ψ^k represent nonlinear equality and inequality constraints, and i, j, k, and q denote the total number of the unknown variables, the nonlinear equality constraints, then nonlinear non-equality constraints, and then linear constraints, respectively. The constraint functions are described in more details below.

6.1 Constraints

In our minimization problem, constraints can be categorized into two types: linear and nonlinear. The first type specifies the range of all admissible geometrical parameters, such as the length of the unit, which cannot be smaller than a certain value given the initial set of geometrical parameters as our input. The second type is either a solution for the equality equation to ensure the rigid-ruling foldability, smoothness and lockability of the lens-box pattern, or in the form of inequality that must be satisfied to avoid overlap in the connector unit. More specifically, the list of applied constraints are as follows.

Lens unit:

l1) Smooth folding: Eq. (3) gives the admissible value of μ for the given set of the parameters and input folding parameter v^R to ensure smooth folding (with smoothness level of C^2) conditions for the lens unit.

l2) Rigid-ruling foldability: Eq. (14) ensures the existence of a continuous solution for the folding of the lens unit.

I3) Smooth connection: Eq. (43) ensures that the connection between the two units is smooth with a smoothness level of C^1.

<u>Waterbomb connector unit:</u>

I4) Flat-foldability ignoring the panel or edge overlap: satisfying Eq. (20) gives geometric parameter 'a'

I5) Panel protrusion beyond the target surface: Eq. (21)

I6) Overlap of the two folded vertices $\hat{f}(V_{13})$ and $\hat{f}(V_{14})$: Eq. (22)

I7) Penetration of the two creases $\overline{\hat{f}(V_{13})\,\hat{f}(V_5)}$ and $\overline{\hat{f}(V_{13})\,\hat{f}(V_{15})}$ into the crease $\overline{\hat{f}(V_{13})\,\hat{f}(V_9)}$: Eq. (23)

I8) Overlap between to connecting lens box unit: Eq. (24)

I9) Overlap between two adjacent folded lens units along their sides when $\kappa_{II} < 0$: Eq. (25)

The other linear parameters are only needed for the physical realization of the lens-box unit.

6.2 Objective Function

The objective function minimizes the difference between the leg distance $v^{R\star}$ that is calculated using all geometrical parameters satisfying folding onto a prescribed curved surface and our initially assumed value of $v^{R\star}$ denoted here with $v_0^{R\star}$, i.e.,

$$E_i = \left(v^{R\star}(p^i) - v_0^{R\star}\right)^2. \tag{61}$$

The objective function is formulated to preserve the initial vertex positions as close as possible to the $v^{R\star}$ during optimization processes; it has no other physical significance.

6.3 Numerical Optimization Approach

We implement the numerical optimization algorithm in Mathematica using the interior-point method (FindMinimum). The initial guesses for the design variables are chosen based on our

understanding of the problem and the geometric attributes of the lens box unit cell; they are then refined iteratively to approach a feasible solution. The optimization is considered successful if the algorithm returns a solution that satisfies all constraints within a given numerical tolerance (i.e., the maximum residual among all constraints is smaller than 10^{-8}) over several hundred iterations.

After convergence, we evaluate the numerical quality of each solution in two ways:

(i) *Feasibility*. We substitute the optimized design variables p^{i*} back into all constraints and measure any violation. For each equality constraint $\Phi^j(p^i) = 0$, we compute the residual $|\Phi^j(p^{i*})|$ across all unit-cell designs. The maximum equality residual is below 10^{-12}. All inequality constraints $\Psi^k(p^i) \leq 0$ (e.g., geometric bounds, angle limits, non-penetration, no-overlap, and rigid-ruling foldability) and all simple variable box bounds ($p_a^q \leq p_q^i \leq p_b^q$) are satisfied with zero violation within numerical precision.

(ii) *First-order optimality*. We restart the interior-point solver from the optimized solution and record the solver diagnostics. The solver-reported feasibility residuals are on the order of $10^{-15} - 10^{-16}$, the KKT (Karush-Kuhn-Tucker) (stationarity) residuals are on the order of 10^{-8}, and the complementarity residuals are on the order of 10^{-20} or smaller.

These values indicate that our designs are numerically feasible to machine precision and satisfy the KKT optimality conditions at the $\mathcal{O}(10^{-8})$ level.

7 Rigidity Analysis

We study the rigidity of our origami shell structure through a set of assumptions. First, we convert our curved-crease unit into its equivalent rigid origami model identified by its rulings pattern (Fig. S21a-left). We also add a diagonal crease line to the quad panels in the lens panel to include its twisting deformation modes. Second, assume all panels as infinitely rigid and the rulings and crease lines as frictionless rotational hinges as shown in Fig. S12. Third, to simplify the formulation, we replace the rigid origami structure with a triangulated network of inextensible struts connected through pin joints (Fig. S21a-right). Now, assuming a bar-and-joint assembly with n_b bars, n_j joints, and rigidity matrix of the replaced bar-and-joint structure with rank ϖ, the

number of infinitesimal mechanisms and self-stress can be obtained using the equilibrium matrix of the structure and noting the relation $m = 3n_j - \varpi - 6$ and the number of self-stress states as $n_s = n_b - \varpi$ [25]. To study the rigidity of our lens box unit in the lock mode when the unit is under compression, we also assume the overlapping vertices as a single vertex. Therefore, we model flat-folded connectors with two crossing bars as shown in Fig. S21.

Our rigidity analysis shows that the rigidity of the lens-box is insensitive to the discretization of the curved-crease by ruling lines. Therefore, for simplicity, we utilize the lowest-level discretization model (Fig. S21a-right) with the geometric parameters used to construct the pattern shown in Fig. 3. Our results show that the locked waterbomb lens-box has one infinitesimal mechanism $m = 1$ with two states of self-stress (related to two cross-type bars used for the simulation of the flat-folded connectors) (Fig. 21b). This infinitesimal deformation mode corresponds to the twisting deformation of the unit, as can be observed in our rigid folding origami simulation using *Freeform* origami software [19]. We note that eliminating one of the bars in each set of the cross-type bars (equivalent to adding flexibility to connector units) does not change the number of infinitesimal mechanisms, while the states of self-stress reduce to zero. Adding the bar b1 between joint 2 and 3 (see Fig. S21c) reduces the number of infinitesimal modes to zero (i.e., $m = 0$). Of course, this is not the only way to limit the infinitesimal DOFs of our system; for example, we can achieve the same outcome by adding a bar connecting joint 5 to 2. Next, we explain the implication of this result.

While our simplified truss-based model qualitatively demonstrates the rigidity of our tendon-origami structure, it is not suitable for analyzing its mechanical response. A more advanced reduced truss-based model – accounting for panel bending stiffness and interfacial contacts between connector panels – would be required for such an analysis, which is beyond the scope of this work.

7.1 Using Tendons to Impart Rigidity

While the integration of the additional rigid bar discussed in the previous section can effectively result in zero infinitesimal mechanisms, fixing the unit spatially converts it into a non-reconfigurable unit. Alternatively, we can search for states of self-stress in a system and add non-rigid members, such as tendons, because the first-order infinitesimal mechanisms can be stabilized

by states of self-stress [26, 27]. A simple approach is to add another rigid bar, b2, to the bar-and-joint system shown in Fig. S21d. Our results show that in this case, we have one state of self-stress and zero infinitesimal mechanisms. Therefore, to impart rigidity to our locked unit, it is sufficient to implement two stretched tendons: one from joint 3 to 2 and another from joint 1 to 4 (Fig. S21a-right). Because these tendons are located below, they cannot fully constrain the DOFs of the waterbomb connector units. To fully confine the connectors, we need another tendon on the top of the unit, as illustrated in Fig. 3 (not shown in Fig. S21a). Next, we discuss an important implication of utilizing tendons that is its capacity for tuning the stiffness of the structure.

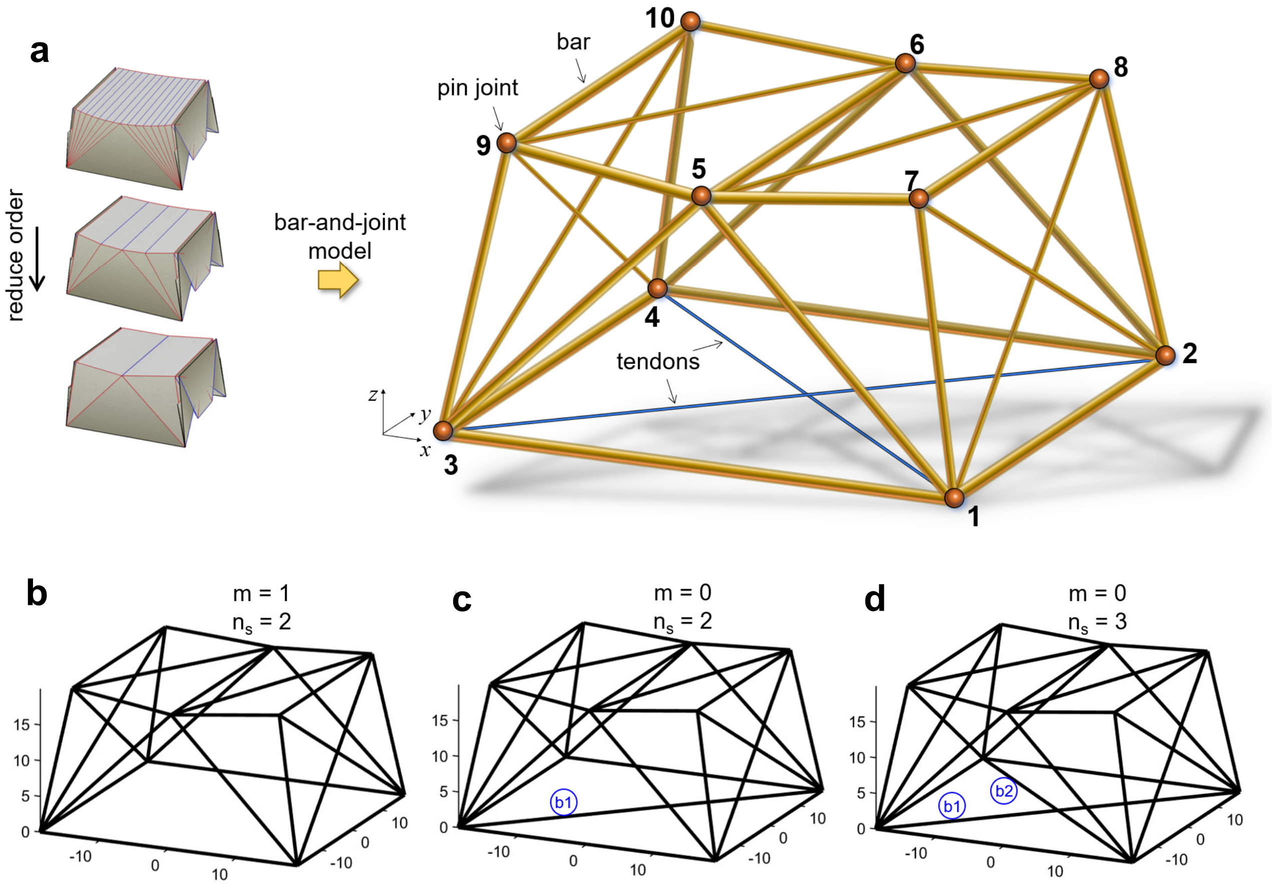

Fig. S21. Bar-and-joint assembly of a waterbomb lens-box unit in the lock state and rigidity simulation. (a) Simplification of the ruling discretization of the lens-box unit (left) and schematic of the bar and joint model (right) for the lowest-order ruling discretization. It also includes two cross-type tendons as we obtained as the condition of the multiaxial rigidity in our final design. Bars unrelated to real crease lines are represented by slim bars. Rigidity simulation of the equivalent bar-and-joint model of the lens-box, illustrating its infinitesimal mechanisms m and states of self-stress n_s for three cases: (b) when the lens-box is only locked without additional constraints, (c) after adding bar b1 to the bottom of the unit, and (d) after adding two cross-type bars b1 and b2 to introduce additional self-stress sates related to these bars. The two self-stress states in (b) relate to the two cross-type bars modeling the locked connector units. Allowing the flexibility of the connector units (by removing both cross-type bars) reveals $n_s = 0$ and $m = 3$. Geometric parameters for the shown unit (in mm): $v = 60, \ell_0 = 14.3, u = 16, \delta = 25°, R_c = R_{GI} = 70$, and $u_c = 12.92$ with folding parameter $v^R = v^L = v/2$.

7.2 Tensegrity Notions to Tune Stiffness

To understand how tensegrity notions can be used to tune the stiffness of our origami shell structures with tendons of pre-tension T, we refer to the example discussed in [26]. Fig. S22 shows this example which is the simplest tensegrity frame, with bar AB and two tendons AC and AB, having an infinitesimal mode. When there is a state of self-stress, i.e., the two tendons are prestressed with tension T_0, an external load with a magnitude of $P = 2T_0\Delta/a$ can be supported. As discussed in [26], in the presence of the self-stress state in these members, the stiffness of the joint depends on the prestress level and the kinematic of the infinitesimal mode but it remains independent of both the cross-sectional area of the members and their modulus of elasticity. The dependency of tensegrity structures' stiffness on their level of prestress and the proper discussion thereof is reported in [26-28]. We emphasize that while this simple example can to certain extent explain our experimental observation, formulating our problem which involves, deformable elastic panels, contact, curved panels, and tendons that pass through the panels, is far more complex and challenging, the subject of ongoing investigation.

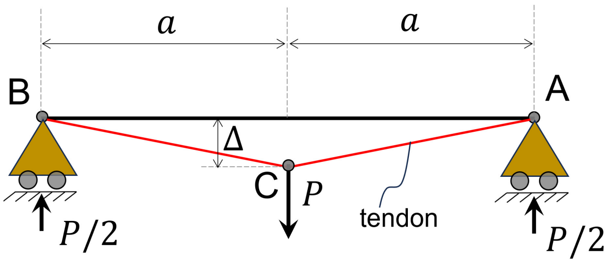

Fig. S22. The simplest tensegrity frame showing an infinitesimal mode of deformation. The stiffness of the system in response to external force P depends on the magnitude of the pre-stress T_0 in the tendon in unloaded state of the frame. In the undeformed configuration AB = 2a, AC = BC = a.

8 Experiments

8.1 Fabrication and Testing Methods

All the origami prototypes and test specimens are made from cellulose paperboard material (200 gm^{-2} Fabriano Craft paper) with a dry thickness t = 0.21 mm. To fabricate single-layer shell structures, each flat sheet is first perforated via laser cutting (CM1290 laser cutter, SignCut Inc.) along prescribed crease patterns, followed by manual folding. Fold lines are obtained with cuts of

1.2 mm length spaced uniformly at 1 mm intervals. To fabricate the rigid shell structures, the panels of the waterbomb connector units are glued (using commercial Polyvinyl acetate).

Experiments are performed with a STEP-lab electrodynamic mechanical testing machine (STEP ENGINEERING S.R.L., Resana, Treviso, Italy) and with load-cells (AEP Transducers, Cognento, Italy) with load capacity of 1kN (Type TSC3), under displacement-control and quasi-static ramp loading conditions with a strain rate of 10^{-3} s^{-1}. Manufacturing and testing samples are conducted at ambient temperature (22 °C) with a relative humidity of around 30-40%. In the three-point bending experiment, the specimens are located on two cylindrical beds with a diameter of 30 mm, and loading starts by lowering an indenter with a cylindrical head of the same diameter made of smooth steel. The displacement data are collected from the crosshead displacement readings as they provide an acceptable level of accuracy for our purpose as discussed in [29]. Cyclic compression-decompression experiments are carried out using a displacement-control module at the same strain rate. The tensile Young's modulus and strength of our paperboard sheets in the machine direction (MD direction) is about 7.9 GPa and 57 MPa, respectively, roughly two times higher than the values obtained in the cross direction (CD direction) as obtained in [29]. All specimens and prototypes are fabricated such that the long diagonal of their unfolded lens panels (i.e., the symmetry axis of the lens panel) stays parallel to the MD direction of the paper sheet. To tune the prestress in the tendons, we fabricated a gear-based tuning mechanism as described below.

8.2 A Gear-Based Mechanism for Adjustment of Pre-Tension in Tendons

To precisely adjust the stiffness of our locked lens-box shells, we design a simple gear-based tuning mechanism (Fig. S23a, b, c). This mechanism allows us not only to create controlled pre-tension in the tendons of our tensegrity-inspired lens-box system but also to lock after applying a given stretch to the tendon, thereby maintaining the integrity of both the tendons and the origami shell. Fig. S23 illustrates the gear-based tuning mechanism with its components. Fig. S23d shows an orthographic view of the assembled mechanism, while Fig. S23e displays the opposite side of the mechanism, featuring an additional panel (internal fixing panel) located on the internal side of the origami shell (see also Fig. S23c). Three small screws (not shown in schematic) are used to fasten the tuning mechanism to the origami shell in its locked configuration, as depicted in Fig. S23c and a-bottom. Fig. S23f provides a detailed view of the internal components of the tuning mechanism. As depicted, the gears can only rotate in one direction, with any opposite motion

counteracted by the stoppers. A rubber band is used to pull the stopper towards the gear shaft, thereby conditioning the stopper's motion. Gears and stoppers are glued onto their shafts, allowing the shafts to rotate freely within their respective holes. Additionally, a tiny hole drilled through the thickness of the gear shafts is used to secure the end of the tendons.

The tuning mechanism is made of high-density wood fiberboard (Masonite Panel) with a thickness of 3 mm for all planar parts, such as gears, stoppers, and panels, along with hardwood dowel rods with a diameter of 5 mm and a length of 20 mm for the shafts. Tendons are made of monofilament nylon fishing line (Red Wolf, Canadian Tire Corporation, Canada) with a nominal load capacity of 3.6 kg and a diameter of about 0.1 mm. While we did not perform experiments for measuring the properties of the fishing line, its Young's modulus varies between 1.5-2.4 GPa, and its ultimate strength between 0.25-0.9 GPa [30, 31].

The assembly of tendons and the tuning mechanism is as follows. Tendons are embedded in a parallel array (Fig. S23a-bottom), differing from cross-type arrays that can also restrict twisting deformation mode, as discussed in Section 7. The reason for this choice is that our sample is subjected to pure bending deformation, experiencing no twisting deflection. To control the pre-tension in tendons array, two separate tuning mechanisms are installed on the right-hand-side and left-hand-side of the origami structure, as illustrated in Fig. S23a. Each top tendon is linked to a gear on the right-hand-side tuning mechanism, while each pair of tendons below, within each row, is connected to a gear on the left-hand-side tuning mechanism. To adjust the stiffness of our lens-box shell structure, we modify the pre-tension in each individual tendon by gradually turning their connected gear. Since these tendons wrap around the gear shaft, the circular displacement of the gear shaft can determine the amount of stretch in each tendon with each rotation step. Consequently, within each step of rotation (gear tooth movement), the individual tendons can be stretched by $l_G = \pi d_s / n_G$, where d_s represents the gear shaft diameter and n_G the number of gear teeth. Upon using the design parameters $d_s = 5$ mm and $n_G = 10$, we obtain $l_G = \pi/2$ mm. To enhance the resolution of the tuning, i.e., the pre-tension in the tendons, the number of gear teeth should be increased.

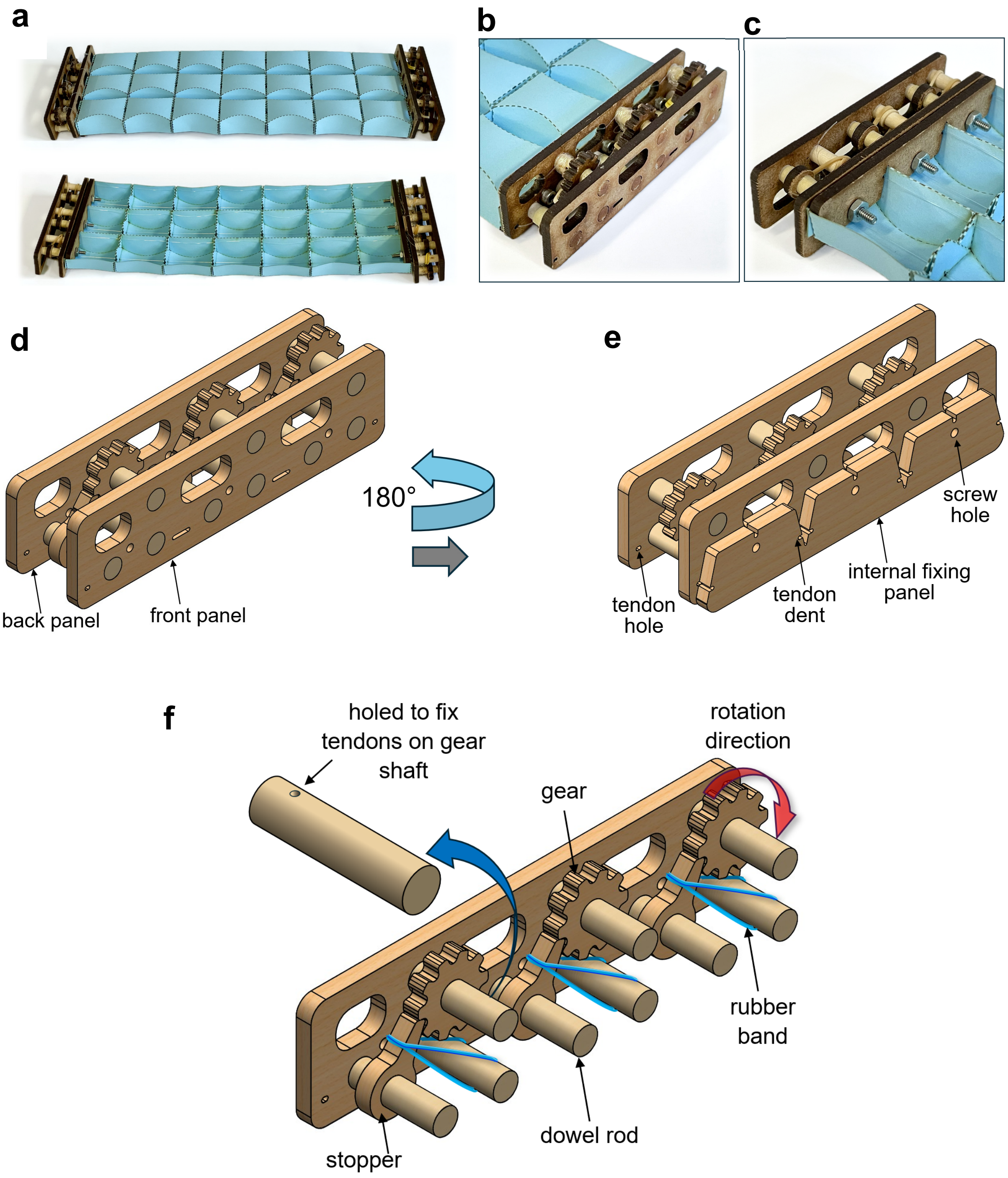

Fig. S23. Construction details of the tuning mechanism. (a) Paperboard prototype from front and behind (below) view along with the zoomed view of the tuning mechanism from front side (b) and back side (c). (d) Drawing of the tuning mechanism installed on the external side of the sample. (e) Illustration of the internal fixing panel and its relative position within the tuning mechanism. This panel secures the tuning mechanism shown in (d) in place. (f) Drawing depicting the internal details and components of the tuning mechanism. It also schematically illustrates the allowable direction of the gear rotation; the rotation along the opposite direction is impossible as it is blocked by the stopper. A rubber band is used to create a small compressive force towards the gear shaft, preventing the stopper's free motion. Gears and stoppers are glued to their shafts, allowing rotation within their holes. A tiny through-the-thickness hole is drilled in the gear shafts to fix the tendon.

8.3 Three-Point Bending Experiment

To assess the role of the tendons' pre-tension on the flexural rigidity of folded shells made of paperboard, we inversely design a specimen with convex lens-box units to lock into zero global curvature, i.e., to be flat in the lock configuration. The specimen design consists of 7 units along the length and 3 units along the width. Initially, the tendons are loose, but not slack. Prior to the start of the test, we repeatedly tighten each set of the upper and lower tendons equally and measure the force that ensures there is a sufficient pre-tension to observe noticeable initial stiffness. Furthermore, by adjusting the pre-tension in the upper tendons we ensure that the geometry of the unloaded specimen remains approximately flat in each experiment. From this point onward, we start to increase the prestress in tendons discreetly, and at each increment, we perform three-point loading-unloading bending experiments for 4 cycles. The pre-tension is applied equally in all lower tendons. We also apply equal pre-tenson in the upper tendons whenever we need to flatten the geometry of the unloaded tendon-origami shell after applying the pre-tension in the lower tendons. As a result, we only apply one stretch increment at steps 4, 7, 9 and 10 (shown with circled numbers of darker shades in Fig. 4b and c) in the upper tendons.

The results in Fig. 4c show that the load-displacement response of the specimen increases by raising the pretension in the tendons. The tighter the pretension, the stiffer the response of the system and the larger the magnitude of the maximum load at a given displacement. This behavior is consistent over the four cycles of repetition. Some hysteresis can be observed, which may result from the viscoelastic-plastic response of our base paperboard material and/or the initiation or propagation of damage in the fold lines cumulated at each cycle.

Supplementary References

1. Mirzajanzadeh, M. & Pasini, D. Reprogrammable curved-straight origami: multimorphability and volumetric tunability. *Sci. Adv.* **11**, eadu4678 (2025).
2. Demaine, E. D., Demaine, M. L., Huffman, D. A., Koschitz, D. & Tachi, T. Characterization of curved creases and rulings: design and analysis of lens tessellations. In *Origami 6*, Miura, K. & Kawasaki, T. (eds.) (A. K. Peters/CRC Press, 2015), 209–230.
3. Fuks, D. B. & Tabachnikov, S. Developable surfaces. In *Mathematical Omnibus: Thirty Lectures on Classic Mathematics* (American Mathematical Society, 2007).
4. Demaine, E. D. & O'Rourke, J. *Geometric Folding Algorithms: Linkages, Origami, Polyhedra* (Cambridge University Press, 2007).
5. Fuchs, D. & Tabachnikov, S. More on paperfolding. *Am. Math. Mon.* **106**, 27–35 (1999).
6. Demaine, E. D., Demaine, M. L., Hart, V., Price, G. N. & Tachi, T. (Non)existence of pleated folds: how paper folds between creases. *Graphs Comb.* **27**, 377–397 (2011).
7. Spivak, M. D. *A Comprehensive Introduction to Differential Geometry* (Publish or Perish, 1970).
8. do Carmo, M. P. *Differential Geometry of Curves and Surfaces: Revised and Updated Second Edition* (Courier Dover Publications, 2016).
9. Pottmann, H. & Wallner, J. *Computational Line Geometry* (Springer, Berlin, 2010).
10. Gauss, K. F. *General Investigations of Curved Surfaces* (BoD–Books on Demand, 2022).
11. Mundilova, K. Gluing and creasing paper along curves: computational methods for analysis and design. PhD thesis, Massachusetts Institute of Technology (2024).
12. Tachi, T. Composite rigid-foldable curved origami structure. In *Proceedings of Transformables* (2013), 18–20.
13. Chen, Y., Feng, H., Ma, J., Peng, R. & You, Z. Symmetric waterbomb origami. *Proc. R. Soc. A* **472**, 20150846 (2016).
14. Kawasaki, T. On the relation between mountain-creases and valley-creases of a flat origami. In *Proceedings of the First International Meeting of Origami Science and Technology*, Sugihara, K. & Lang, R. J. (eds.) (Origami Society of Japan, Tokyo, 1991), 229–237.
15. Hull, T. On the mathematics of flat origamis. *Congr. Numer.* **100**, 215–224 (1994).
16. Kasahara, K. & Maekawa, J. *Viva! Origami* (Sanrio, Tokyo, 1983).
17. Hull, T. C. Modelling the folding of paper into three dimensions using affine transformations. *Linear Algebra Appl.* **348**, 273–282 (2002).
18. Akitaya, H., Demaine, E. D., Horiyama, T., Hull, T. C., Ku, J. S. & Tachi, T. Rigid foldability is NP-hard. Preprint at https://arxiv.org/abs/1812.01160 (2018).
19. Tachi, T. Freeform variations of origami. *J. Geom. Graph.* **14**, 203–215 (2010).

20. Abel, Z., Cantarella, J., Demaine, E. D., Eppstein, D., Hull, T. C., Ku, J. S., Lang, R. J. & Tachi, T. Rigid origami vertices: conditions and forcing sets. Preprint at https://arxiv.org/abs/1507.01644 (2015).

21. Farnham, J., Hull, T. C. & Rumbolt, A. Rigid folding equations of degree-6 origami vertices. *Proc. R. Soc. A* **478**, 20220051 (2022).

22. Tachi, T. Design of infinitesimally and finitely flexible origami based on reciprocal figures. *J. Geom. Graph.* **16**, 223–234 (2012).

23. Foschi, R., Hull, T. C. & Ku, J. S. Explicit kinematic equations for degree-4 rigid origami vertices, Euclidean and non-Euclidean. *Phys. Rev. E* **106**, 055001 (2022).

24. Tachi, T. Generalization of rigid-foldable quadrilateral-mesh origami. *J. Int. Assoc. Shell Spatial Struct.* **50**, 173–179 (2009).

25. Pellegrino, S. & Calladine, C. R. Matrix analysis of statically and kinematically indeterminate frameworks. *Int. J. Solids Struct.* **22**, 409–428 (1986).

26. Calladine, C. R. Buckminster Fuller's "tensegrity" structures and Clerk Maxwell's rules for the construction of stiff frames. *Int. J. Solids Struct.* **14**, 161–172 (1978).

27. Guest, S. The stiffness of prestressed frameworks: a unifying approach. *Int. J. Solids Struct.* **43**, 842–854 (2006).

28. Guest, S. D. The stiffness of tensegrity structures. *IMA J. Appl. Math.* **76**, 57–66 (2011).

29. Jamalimehr, A., Mirzajanzadeh, M., Akbarzadeh, A. & Pasini, D. Rigidly flat-foldable class of lockable origami-inspired metamaterials with topological stiff states. *Nat. Commun.* **13**, 1816 (2022).

30. Haines, C. S. et al. Artificial muscles from fishing line and sewing thread. *Science* **343**, 868–872 (2014).

31. Cherubini, A., Moretti, G., Vertechy, R. & Fontana, M. Experimental characterization of thermally activated artificial muscles based on coiled nylon fishing lines. *AIP Adv.* **5**, 067158 (2015).
