# Supplementary material for: Smooth doubly curved origami shells with reprogrammable rigidity
Source: Nat Commun. 2026 Feb 13;17:2729. doi: 10.1038/s41467-026-69562-2 (PMC13013821; doi:10.1038/s41467-026-69562-2)
Supplement: Supplementary file 2 — Description of Additional Supplementary Files [file 41467_2026_69562_MOESM2_ESM.pdf]

Description of Additional Supplementary Files:

Supplementary Movie 1: Demonstration of a flat tendon-driven origami metamaterial showcasing its stiffness reprogrammability. The video highlights the transition from soft to stiff configurations and illustrates how tendons dynamically control the material's mechanical response.

Supplementary Movie 2: Demonstration of a tendon-driven origami shell transitioning from a floppy, formless state to a smoothly curved, load-bearing structure. The video highlights how the embedded tendon mechanism enables controlled and precise deployment into the programmed curvature.
